# Supplementary material for: Trade-offs in land-based carbon removal measures under 1.5 °C and 2 °C futures
Source: Nat Commun. 2024 Mar 14;15:2297. doi: 10.1038/s41467-024-46575-3 (PMC10940641; doi:10.1038/s41467-024-46575-3)
Supplement: Supplementary file 1 — Supplementary Information [file 41467_2024_46575_MOESM1_ESM.pdf]

Article

Trade-Offs in Land-Based Carbon Removal Measures under 1.5°C and 2°C Futures

Supplementary Information

Contents

S1. IPCC AR6 Scenario Database 2
S2. Supplementary description of the methodology 7
S2.1. Socioeconomic and agricultural productivity drivers 10
S2.2. Logit land allocation and parameters 12
S2.3. Land rental profit and shadow profit 13
S2.4. Land system mitigation policy 14
S2.5. Land carbon density 15
S2.6. Agricultural demand responses 18
S2.7. Residual biomass supply 19
S2.8. Bioenergy carbon flows, price transmission, and budget limits on crediting BECCS 20
S3. Supplementary results and discussion 22
S3.1. GCAM reference scenario 22
S3.2. Supplementary discussion of results in mitigation pathways 24
S3.3. Unraveling the complexity of land carbon removal intensity 27
S3.4. Agricultural prices vs. land carbon removal intensity 28
S3.5. Limitations and future work 28
Supplementary references 64

List of Supplementary Tables and Figures

Table S1 | Regional mapping between GCAM 32 regions and IPCC R10 regions 8
Table S2 | Agricultural commodity mapping between GCAM commodity and FAO. 9
Table S3 | Shadow price of carbon by sector in GCAM scenarios (2010$ per tCO2). 31
Table S4 | Distribution of carbon prices in AR6 pathways (2010$ per tCO_2). 32
Table S5 | Connecting land use to land-based carbon removal. 33
Table S6 | Deriving land carbon removal intensity. 34
Table S7 | Regression results for agricultural price models. 37
Table S8 | Relationship between agricultural prices and land removal intensity in mitigation pathways. 38

Fig. S1 | Histogram of the pathways in the IPCC Sixth Assessment Report (AR6) Scenario Database by model (A) and temperature (B), and cumulative CO_2 emissions (2020 – 2100) (C). 3
Fig. S2 | Projections of cumulative carbon dioxide emissions and removals by sectors in IPCC AR6 pathways. 4
Fig. S3 | Relationship between cumulative (2020 – 2100) afforestation carbon removal (Land: Afforestation) and LULUCF in IPCC AR6 pathways (n = 264). 5
Fig. S4 | Relationship of the cumulative (2020 - 2100) carbon dioxide removal between LULUCF and BECCS in AR6 pathways by modeling framework. 6
Fig. S5 | Macroeconomic and agricultural productivity drivers in GCAM. 11
Fig. S6 | Land nesting structure in GCAM. 13
Fig. S7 | Land carbon density distributions. 16
Fig. S8 | Land carbon density maps. 17
Fig. S9 | Difference of land carbon density relative to cropland. 18
Fig. S10 | Food demand nesting structure in GCAM v6. 19
Fig. S11 | Schematic of biomass flows in GCAM v6. 22
Fig. S12 | Summary of key results in the reference projection. 23
Fig. S13 | Shadow price of carbon (Panel A) and carbon mitigation relative to the reference scenario (Panel B) by sector in 2025 to 2100. 39
Fig. S14 | Decomposition of global 2020 – 2100 cumulative (Panel A) and annual (Panel B) carbon dioxide emissions. 40
Fig. S15 | Decomposition of global (Panel A) and regional (Panel B) cumulative carbon dioxide emissions. ... 41
Fig. S16 | Projections of global 2020 – 2100 cumulative (A) and annual (B) non-carbon dioxide GHG emissions. 42
Fig. S17 | Projections of global 2020 – 2100 cumulative (A) and annual (B) primary bioenergy supply and demand. 43
Fig. S18 | Projections of 2020 – 2100 regional (IPCC R10) cumulative primary bioenergy supply and demand. 44
Fig. S19 | Projections of global 2020 – 2100 cumulative (A) and annual (B) primary bioenergy by demand sectors and CCS technology deployment. 45
Fig. S20 | Projections of global 2020 – 2100 cumulative primary bioenergy by demand sectors, CCS technology deployment, and supply sectors. 46
Fig. S21 | Projections of global 2020 – 2100 cumulative (A) and annual (B) primary biogenic carbon flow in CCS sectors by capture. 47
Fig. S22 | Projections of purpose-grown bioenergy crop yield at the world (Panel A) and regional (Panel B) levels. 48
Fig. S23 | Global land use and land use change. 49
Fig. S24 | Global and regional mean (2020 – 2100) changes in land use. 50
Fig. S25 | Net LULUCF CO_2 emissions. 51

Fig. S26 | Land carbon removal intensity. 52
Fig. S27 | Land carbon removal intensity (alternative method for LULUCF removal intensity). 53
Fig. S28 | Relationship between 2020 – 2100 mean land-based carbon dioxide removal (CDR) and its corresponding land implication. 54
Fig. S29 | Relationship between 2020 – 2050 mean land-based carbon dioxide removal (CDR) and its corresponding land implication. 55
Fig. S30 | BECCS land carbon removal intensity decomposition. 56
Fig. S31 | Agricultural price index distributions across GCAM sector-region combinations. 57
Fig. S32 | Relationship between shadow price of carbon and land use impacts (reference = 0 Mha).............. 58
Fig. S33 | Relationship between staple crop price impact (reference = 1) and non-energy cropland impacts (reference = 0 Mha) at the world level (Panel A) and by AR6 R10 regions (Panel B)...................................... 59
Fig. S34 | Changes in staple crop production, land area, and yield relative to the reference scenario. 60
Fig. S35 | Changes in staple crop demand by source relative to the reference scenario................................ 61
Fig. S36 | Projections of dietary energy availability at the world (Panel A) and regional (Panel B) levels. 62
Fig. S37 | Relationship between agricultural prices and land carbon removal intensity. 63

S1. IPCC AR6 Scenario Database

The Intergovernmental Panel on Climate Change (IPCC) recently released a Scenario Database[1] as part of its 6th Assessment Report (AR6)[2], containing global pathways submitted by modeling teams worldwide. Each pathway describes a future of socioeconomic development, energy and land systems, greenhouse gas emissions, and climate, projected to the end of the century based on a set of socioeconomic drivers and technological and policy assumptions. The Scenario Database has a vetting process to ensure key indicators are reasonable in baseline periods, and pathways that pass the vetting and include projection to the end of the century may receive a climate classification[3]. Out of the 1202 pathways that passed the vetting. 700 pathways were classified as 2 °C or lower pathways (in C1 – C4 categories).

However, the IPCC vetting process mainly focuses on energy and emissions outcomes, whereas our study concentrates on land-based carbon dioxide removal (CDR) measures. Consequently, we removed 34 pathways (30 GEM-E3_V2021 pathways and 4 EPPA pathways) that did not report bioenergy with carbon capture and storage (BECCS) results. In addition, we added one EPPA pathway back (EPPA 6-Paris2C_OptTax) based on results provided by Fajardy et al. (2021)[4], given the relevance and importance of the study. We also further subset pathways based on cumulative carbon dioxide emissions in 2020 – 2100, i.e., in [175, 1475] $GtCO_2$, to ensure comparability with our pathways, resulting in 604 pathways that fit the criteria for our study. Out of these pathways, 101 were included in the IPCC Special Report on Global Warming of 1.5 °C (SR15)[5]. Also, there are 29 GCAM pathways, which consist of 24 pathways from Ou et al. (2021) that use GCAM v5.3, 4 NGFS pathways that use GCAM v5.2 and v5.3, and 1 GCAM v4.2 pathway from the SSP & SR15 database. More details about the AR6 pathways can be found in Annex III of the IPCC AR6 WGIII report[3].

Fig. S1 displays a histogram of the 604 pathways categorized by model and the distribution of temperature change and cumulative emission outcomes. Meanwhile, **Fig. S2** presents the cumulative carbon dioxide emissions and removals projections by sectors and technologies. BECCS is expected to be the largest CDR measure. While most pathways reported results for BECCS and LULUCF, only a subset of them permitted and reported other CDRs, with 241 pathways including DACCS and 113 pathways involving enhanced weathering. That is, not all models represented all CDR technologies. The reliance on BECCS and A/R removals and energy system mitigation could be less pronounced if higher levels of new CDRs are deployed. It is also worth noting that only a subset of the pathways (n = 264) reported afforestation/reforestation (A/R), or "Land: Afforestation," with the definition and accounting method remains unclear due to the high sensitivity of A/R modeling to factors such as model resolution, gross vs. net accounting, and land transition assumptions. In addition, carbon removals from land use, land-use change, and forestry (LULUCF) are largely driven by A/R in models reporting both results, as seen in **Fig. S3**.

In **Fig. 1** of the main paper, the relationship between LULUCF and BECCS (left panel) and between LULUCF and Energy and Industrial Process (EIP) (right panel) is estimated using quantile regression since the AR6 database is not a statistical sample. However, using mean regression would result in similar results to the median regression. When estimating the relationship between BECCS and LULUCF, we controlled for the carbon budget (CB), resulting in a median regression estimate of $LULUCF = -268 - 0.16 \cdot BECCS + 0.11 \cdot CB + \varepsilon$. That is, for a median pathway, LULUCF would contribute 11% of the removal from a marginal decrease in the carbon budget, while a marginal increase in BECCS could lead to a leakage of 16% due to LULUCF emissions. All coefficients are statistically significant (p-values < 0.01). The mean regression (OLS) result would be $LULUCF = -251 - 0.17 \cdot BECCS + 0.12 \cdot CB + \varepsilon'$ (all p-values < 0.01).

The AR6 pathways serve as a valuable literature reference for comparison to our results. It is important to underscore that the role of land-system mitigation policy has not been thoroughly examined in the AR6 pathways (e.g., limited intra-model LULUCF variation as seen in **Fig. S4**) or recent studies. Therefore, the pathways developed in our study provide an additional policy dimension that is of high importance, complementing the AR6 Scenario Database.

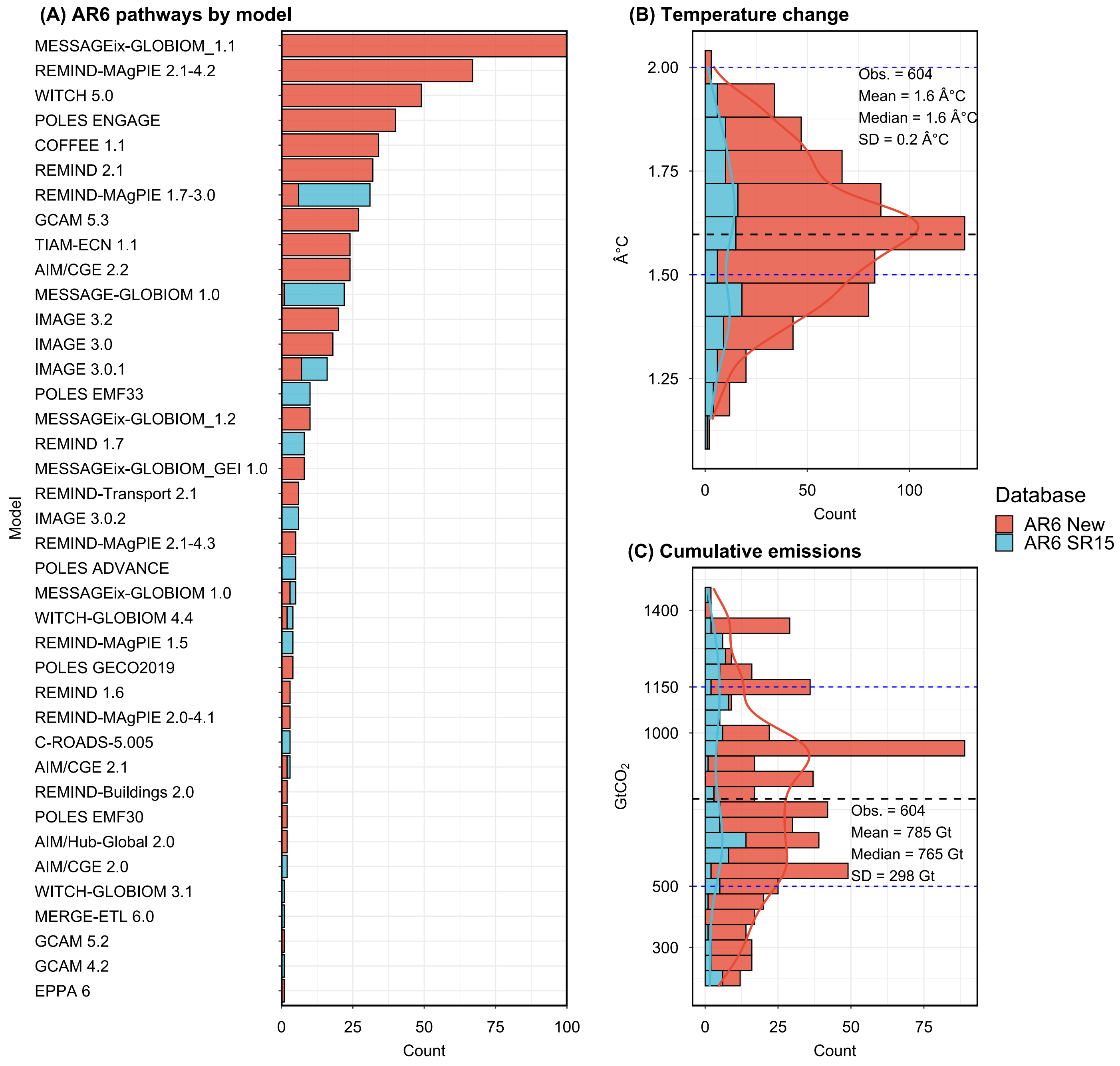

Fig. S1 | Histogram of the pathways in the IPCC Sixth Assessment Report (AR6) Scenario Database by model (A) and temperature (B), and cumulative CO_2 emissions (2020 – 2100) (C). Note that only scenarios with cumulative CO_2 emissions in [175, 1475] $GtCO_2$ are included. There are 604 scenarios, 101 of which were included in the Special Report on Global Warming of 1.5 °C (SR15), as distinguished by filled color. The temperature is the median global mean surface temperature change by 2100 computed by the MAGICC6 model. Data source: https://data.ene.iiasa.ac.at/ar6

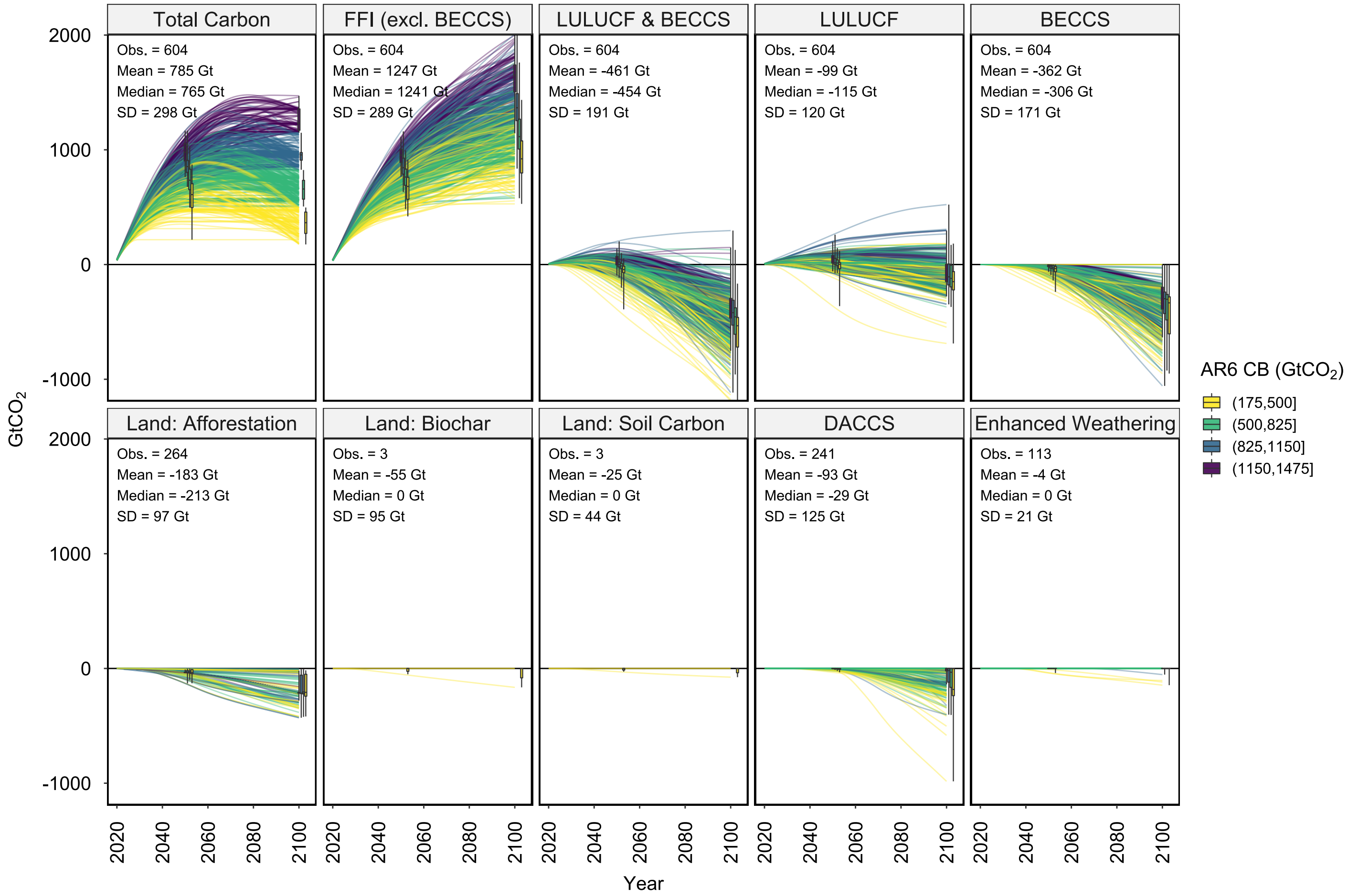

Fig. S2 | Projections of cumulative carbon dioxide emissions and removals by sectors in IPCC AR6 pathways. Lines show cumulative emission projections from IPCC AR6 pathways in C1 – C4 categories with a Carbon Budget (CB) in [175, 1475] $GtCO_2$, distinguished by CB subranges (filled color). The boxplots show the median values (line), the 1st and 3rd quartiles (boxes), and the 0–100 percentile ranges (whiskers) of the available pathways. The summary statistics of the cumulative emissions/removals in 2020 – 2100 are annotated in each panel.

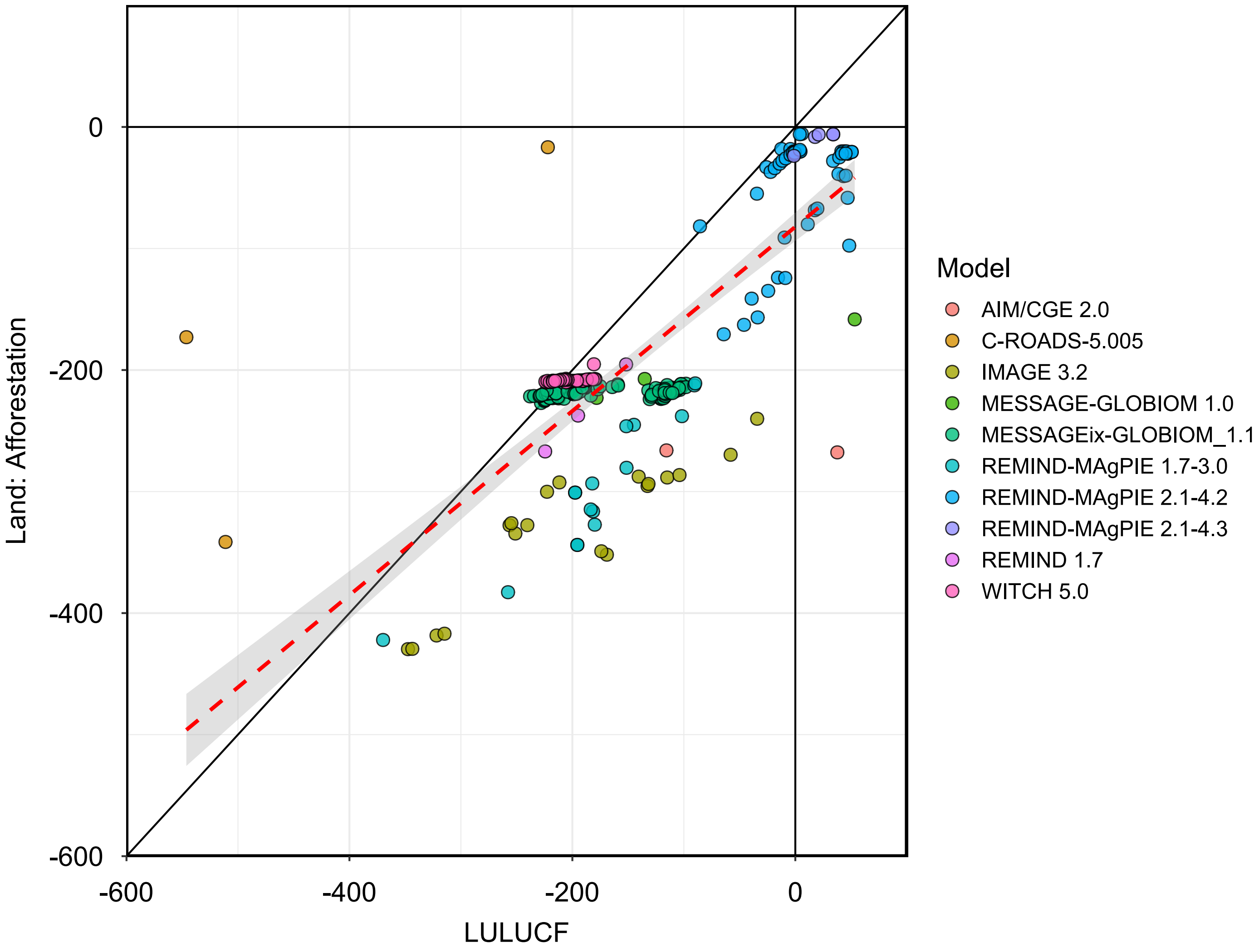

Fig. S3 | Relationship between cumulative (2020 – 2100) afforestation carbon removal (Land: Afforestation) and LULUCF in IPCC AR6 pathways (n = 264). Each dot represents projections of one pathway, distinguished by model (color). See **Fig. S2** for summary statistics of the variables. Linear trend lines (red dotted lines) and 95% CI (grey ribbons) are added.

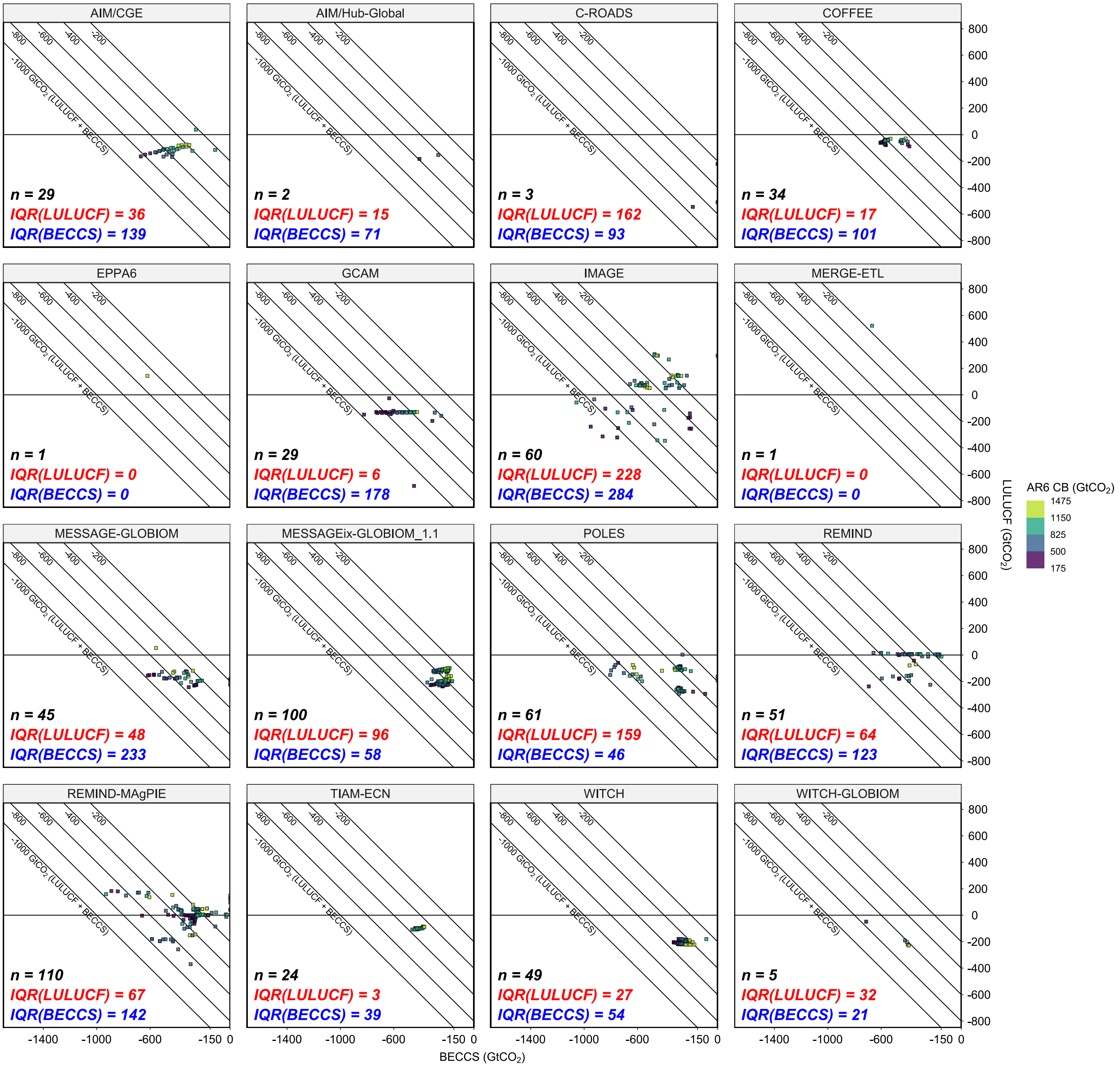

Fig. S4 | Relationship of the cumulative (2020 - 2100) carbon dioxide removal between LULUCF and BECCS in AR6 pathways by modeling framework. Note that this figure facets the left panel of **Fig. 1** in the main paper by modeling framework. See the caption of **Fig. 1** in the main paper for a detailed description. Model version identifiers may be omitted to differentiate the pathways by modeling frameworks. The number of observations (n) and the interquartile range (IQR) of LULUCF and BECCS (in $GtCO_2$) removals are shown in each panel. Data source: AR6 Scenario Database.

S2. Supplementary description of the methodology

An overview of GCAM and scenario design for the present study is provided in the **Method** section in the main paper. Recent developments related to the agriculture and land use (AgLU) module and BECCS modeling in GCAM are documented and tested in a series of studies, e.g., land allocation[6–8], land policy[9], Bioenergy and CCS[10,11], fertilizer[12], water market[13], food demand[14], and trade[15]. More detailed GCAM information can be found in the online documentation (http://jgcri.github.io/gcam-doc/) and recent GCAM applications[16–23]. In this section, we provide more details for key modeling methods and discuss the modifications made to improve the GCAM modeling with a focus on land competition, BECCS, and land carbon policy. Note that, where applicable, GCAM 32 regions are aggregated to IPCC R10 regions for communicating results. The regional mapping is provided in **Table S1**. Note that, where applicable, the carbon dioxide emission/removal values described in the main paper may have been rounded to the nearest 5 $GtCO_2$. The GCAM agricultural sector mapping from FAO commodities is provided in **Table S2**. Where applicable, we utilize the Global Warming Potentials (GWPs) based on a 100-year timeframe (GWP-100) from the IPCC Fourth Assessment Report (AR4)[24] to calculate the CO_2-equivalent (CO_2-eq) emissions for non-CO_2 Greenhouse Gases (GHGs).

Table S1 | Regional mapping between GCAM 32 regions and IPCC R10 regions

GCAM region	IPCC R10 region	IPCC R10 region name
Africa_Eastern<br>Africa_Northern<br>Africa_Southern<br>Africa_Western<br>South Africa	AFRICA	Countries of Sub-Saharan Africa
China<br>Taiwan	CHINA+	Countries of centrally-planned Asia;<br>primarily China
EU-12<br>EU-15<br>Europe_Eastern<br>Europe_Non_EU<br>European Free Trade Association	EUROPE	Eastern and Western Europe (i.e., the EU28)
India<br>South Asia	INDIA+	Countries of South Asia;<br>primarily India
Argentina<br>Brazil<br>Central America and Caribbean<br>Colombia<br>Mexico<br>South America_Northern<br>South America_Southern	LATIN_AM	Countries of Latin America and the Caribbean
Middle East	MIDDLE_EAST	Countries of the Middle East;<br>Iran, Iraq, Israel, Saudi Arabia, Qatar, etc.
Canada<br>USA	NORTH_AM	North America;<br>primarily the United States of America and Canada
Australia_NZ<br>Japan<br>South Korea	PAC_OECD	Pacific OECD
Central Asia<br>Pakistan<br>Russia	REF_ECON	Reforming Economies of Eastern Europe and<br>the Former Soviet Union; primarily Russia
Indonesia<br>Southeast Asia	REST_ASIA	Other countries of Asia

Table S2 | Agricultural commodity mapping between GCAM commodity and FAO. GCAM staple crops include Wheat, Rice, Corn, OtherGrain, and RootTuber. GCAM also uses the primary roundwood data from FAOSTAT for forestry representation.

GCAM commodity	FAO primary item (240)
**Wheat**	Wheat
**Rice**	Rice, paddy
**Corn**	Maize
**OtherGrain**	Barley; Buckwheat; Canary seed; Cereals nes; Fonio; Grain, mixed; Oats; Quinoa; Rye; Triticale; Millet; Sorghum
**Soybean**	Soybeans
**OilPalm (Tree)**	Oil palm fruit
**OilCrop**	Castor oil seed; Hempseed; Jojoba seed; Linseed; Melonseed; Mustard seed; Oilseeds nes; Poppy seed; Rapeseed; Safflower seed; Sesame seed; Sunflower seed
**OilCrop (Tree)**	Coconuts; Kapok fruit; Karite nuts (sheanuts); Olives; Tallowtree seed; Tung nuts
**RootTuber**	Cassava; Potatoes; Roots and tubers nes; Sweet potatoes; Taro (cocoyam); Yams; Yautia (cocoyam);
**FiberCrop**	Fibre crops nes; Flax fibre and tow; Hemp tow waste; Jute; Manila fibre (abaca); Ramie; Seed cotton; Sisal
**NutsSeeds**	Groundnuts, with shell
**NutsSeeds (Tree)**	Almonds, with shell; Areca nuts; Brazil nuts, with shell; Cashew nuts, with shell; Chestnut; Hazelnuts, with shell; Kola nuts; Nuts nes; Pistachios; Walnuts, with shell
**Legumes**	Bambara beans; Beans, dry; Broad beans, horse beans, dry; Chick peas; Cow peas, dry; Lentils; Lupins; Peas, dry; Pigeon peas; Pulses nes; Vetches
**SugarCrop**	Sugar cane; Sugar beet; Sugar crops nes
**Vegetables**	Artichokes; Asparagus; Beans, green; Cabbages and other brassicas; Carobs; Carrots and turnips; Cassava leaves; Cauliflowers and broccoli; Chicory roots; Chillies and peppers, dry; Chillies and peppers, green; Cucumbers and gherkins; Eggplants (aubergines); Garlic; Leeks, other alliaceous vegetables; Lettuce and chicory; Maize, green; Melons, other (inc.cantaloupes); Mushrooms and truffles; Okra; Onions, dry; Onions, shallots, green; Peas, green; Pumpkins, squash and gourds; Spinach; String beans; Tomatoes; Vegetables, fresh nes; Vegetables, leguminous nes; Watermelons
**Fruits**	Berries nes; Blueberries; Cranberries; Currants; Gooseberries; Grapes; Pineapples; Raspberries; Strawberries
**Fruits (Tree)**	Apples; Apricots; Avocados; Bananas; Cashewapple; Cherries; Cherries, sour; Dates; Figs; Fruit, citrus nes; Fruit, fresh nes; Fruit, pome nes; Fruit, stone nes; Fruit, tropical fresh nes; Grapefruit (inc. pomelos); Kiwi fruit; Lemons and limes; Mangoes, mangosteens, guavas; Oranges; Papayas; Peaches and nectarines; Pears; Persimmons; Plantains and others; Plums and sloes; Quinces; Tangerines, mandarins, clementines, satsumas
**MiscCrop**	Anise, badian, fennel, coriander; Ginger; Hops; Pepper (piper spp.); Peppermint; Pyrethrum, dried; Spices nes; Tea; Tobacco, unmanufactured; Vanilla
**MiscCrop (Tree)**	Cinnamon (cannella); Cloves; Cocoa, beans; Coffee, green; Mate; Nutmeg, mace and cardamoms; Rubber, natural;
**FodderGrass**	forage Products; Grasses Nes for forage; Grasses Nes for forage; Rye grass for forage & silage
**FodderHerb**	Alfalfa for forage and silage; Beets for Fodder; Cabbage for Fodder; Carrots for Fodder; Clover for forage and silage; Green Oilseeds for Silage; Leguminous for Silage; Swedes for Fodder; Turnips for Fodder; Vegetables Roots Fodder; Maize for forage and silage; Sorghum for forage and silage
**Beef**	Meat, cattle; Meat, buffalo
**Dairy**	Milk, whole fresh cow; Milk, whole fresh camel; Milk, whole fresh buffalo; Milk, whole fresh goat; Milk, whole fresh sheep
**Pork**	Meat, pig
**Poultry**	Meat, duck; Meat, goose and guinea fowl; Meat, turkey; Meat, chicken; Meat, chicken; Eggs, other bird, in shell; Eggs, hen, in shell;
**SheepGoat**	Meat, sheep; Meat, goat
**OtherMeat &Fish**	Freshwater Fish; Fish, Body Oil; Fish, Liver Oil; Demersal Fish; Pelagic Fish; Marine Fish, Other; Crustaceans; Cephalopods; Molluscs, Other; Aquatic Animals, Others; Aquatic Plants; Meat, Aquatic Mammals; Meat, rabbit; Meat, camel; Meat, other camelids; Meat, horse; Meat, ass; Meat, mule; Meat, other rodents; Meat, bird nes; Meat, game; Meat nes; Offals, edible, cattle; Offals, edible, buffaloes; Offals, pigs, edible; Offals, sheep,edible; Offals, edible, goats; Offals, horses; Offals, edible, camels; Offals, liver chicken; Offals, liver geese; Offals, liver duck; Offals, liver turkeys; Offals nes; Liver prep.; Fat, cattle; Fat, buffaloes; Fat, sheep; Fat, goats; Fat, pigs; Fat, camels; Fat, other camelids; Oils, fats of animal nes; Degras; Grease incl. lanolin wool; Fat nes, prepared; Honey, natural; Snails, not sea

S2.1. Socioeconomic and agricultural productivity drivers

We employ socioeconomic drivers from the SSP2 scenario, in which the global population peaks in 2070 with about 9.58 billion people (+23.6% from 2020) and the world average GDP per capita increases by about 280%, with high regional heterogeneity (**Fig. S5A**).

For crop productivity growth in the GCAM reference scenario, a linear interpolation is applied to the Food and Agriculture Organization (FAO) projections[25,26] to 2030 and 2050, representing a trend of total factor productivity (TFP) improvement. The productivity growth after 2050 is assumed to be slower, e.g., 0.1%-0.3% per year (**Fig. S5B**). For each region, the median growth rate of non-energy crops is used for purpose-grown energy crops. In GCAM v6, the livestock input-output ratio (i.e., the weight of feedstuff intake divided by the weight of livestock products) is calibrated to the base data (e.g., FAOSTAT[27] and IMAGE[28] data) but assumed to be constant in future periods. In this study, we incorporated productivity growth for livestock sectors based on data in IMAGE v3.2 to consider higher future feed conversion efficiency. That is, in addition to the endogenous price-induced productivity responses, exogenous productivity growth driven by technological progress is also considered in both the crop and livestock sectors. The same socioeconomic and agricultural productivity drivers are applied to the reference and alternative scenarios in this study.

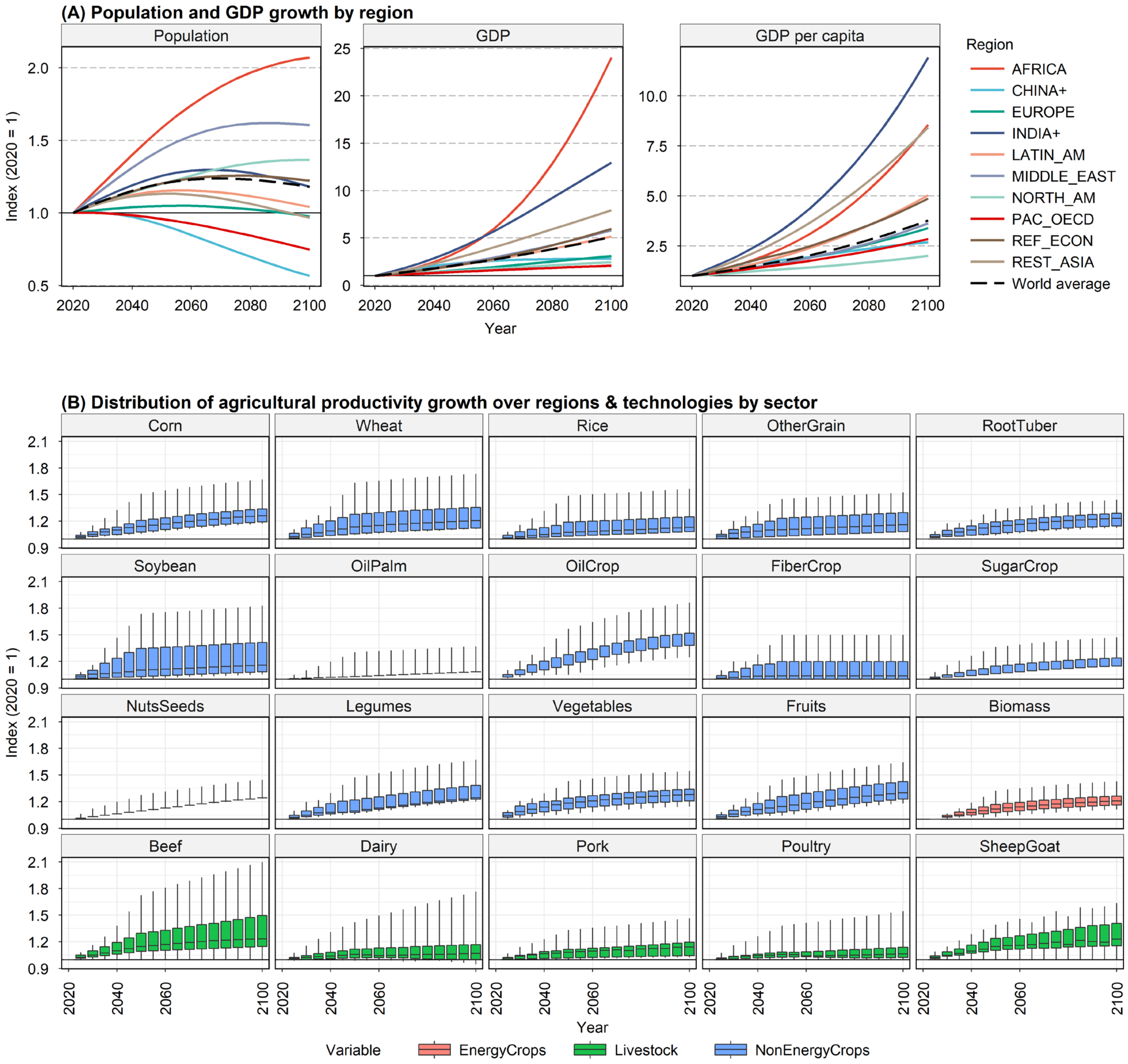

Fig. S5 | Macroeconomic and agricultural productivity drivers in GCAM. Panel A shows the reference projections of population, GDP, and GDP per capita (SSP2). Solid lines show indexes of future changes relative to 2020 of a variable by IPCC R10 regions. See **Table S1** for region mappings. The world average index is shown in dotted lines. Panel B shows the distribution of agricultural productivity changes by sector (2020 = 1). The boxplots show the median values (line), the 1st and 3rd quartiles (boxes), and the 5 – 95 percentile ranges (whiskers) of the index across regions & technologies.

S2.2. Logit land allocation and parameters

Land allocation in GCAM is modeled using the nested logit approach[7], in which landowners in a water-economy region allocate land across uses to maximize a preference-adjusted rental profit[6], subject to physical area constraints and unconditional rental profit distribution that is determined by cost and land productivity information. The derived logit land share in a nest for land i is S_i, which is a function of logit share-weights (α_i), logit exponent (θ), and rental profits (r_i).

$$S_i = \frac{\alpha_i r_i^\theta}{\sum_j \alpha_j r_j^\theta} \quad \text{(S1)}$$

The land sharing maintains physical land balance in a nest, i.e., $\sum_j S_i = 1$, and land competes grossly based on rental profits. While transition-based land use modeling approaches were tested in previous studies, they either relied heavily on assumptions or data not widely exist, e.g., transition function in Ferreira Filho et al. (2015)[29], or did not consider economic responses, e.g., the Markov model in Li and Wu (2022)[30]. The logit land allocation method is parsimonious, flexible in nesting structure, and connects biophysical information to economic behavior[6,7,31–33]. The land nesting structure used in GCAM is shown in **Fig. S6**. Note that purpose-grown energy croplands are introduced in 2025 in GCAM by gradually phasing in their share-weights in the logit nesting structure.

GCAM includes all land data in the base year, as compiled and processed by its designated open-source Moirai land data system[34]. Prior to GCAM v6, the default land data assumption in GCAM was that 90% of natural ecosystems are protected[35]. This implies that 90% of unmanaged forest, unmanaged pasture, grassland, and shrubland were assumed to remain undisturbed in future land reallocation. The assumption results in a rigid natural land supply elasticity because the logit approach depends on both the logit exponent parameters and historical land allocation to determine land supply elasticity. Recently, in GCAM v6, the natural land availability assumption was updated based on regional land availability data compiled in Moirai[36]. The new data indicates that about 30% of the natural land globally is unavailable due to land conservation policies[37] or unsuitability[38]. As tested and demonstrated in Di Vittorio et al. (2023)[23], land availability assumptions could have critical implications on regional land projections. In this study, we recalibrated the land logit exponent parameters to ensure that the own-price natural land supply elasticity remains comparable to the original GCAM assumptions. The recalibration resulted in more rigid land supply elasticities, more consistent with the broader literature[39]. The key land logit exponent parameters are now $\theta^{Arable} = 1.75$, $\theta^{Pas} = 2$, $\theta^{NonPas} = 0.75$, and $\theta^{For} = 1.25$ (**Fig. S6**). These values are lower than the previous ones used in GCAM, which were $\theta^{Arable} = 2$, $\theta^{Pas} = 3$, $\theta^{NonPas} = 1.25$, and $\theta^{For} = 1.75$.

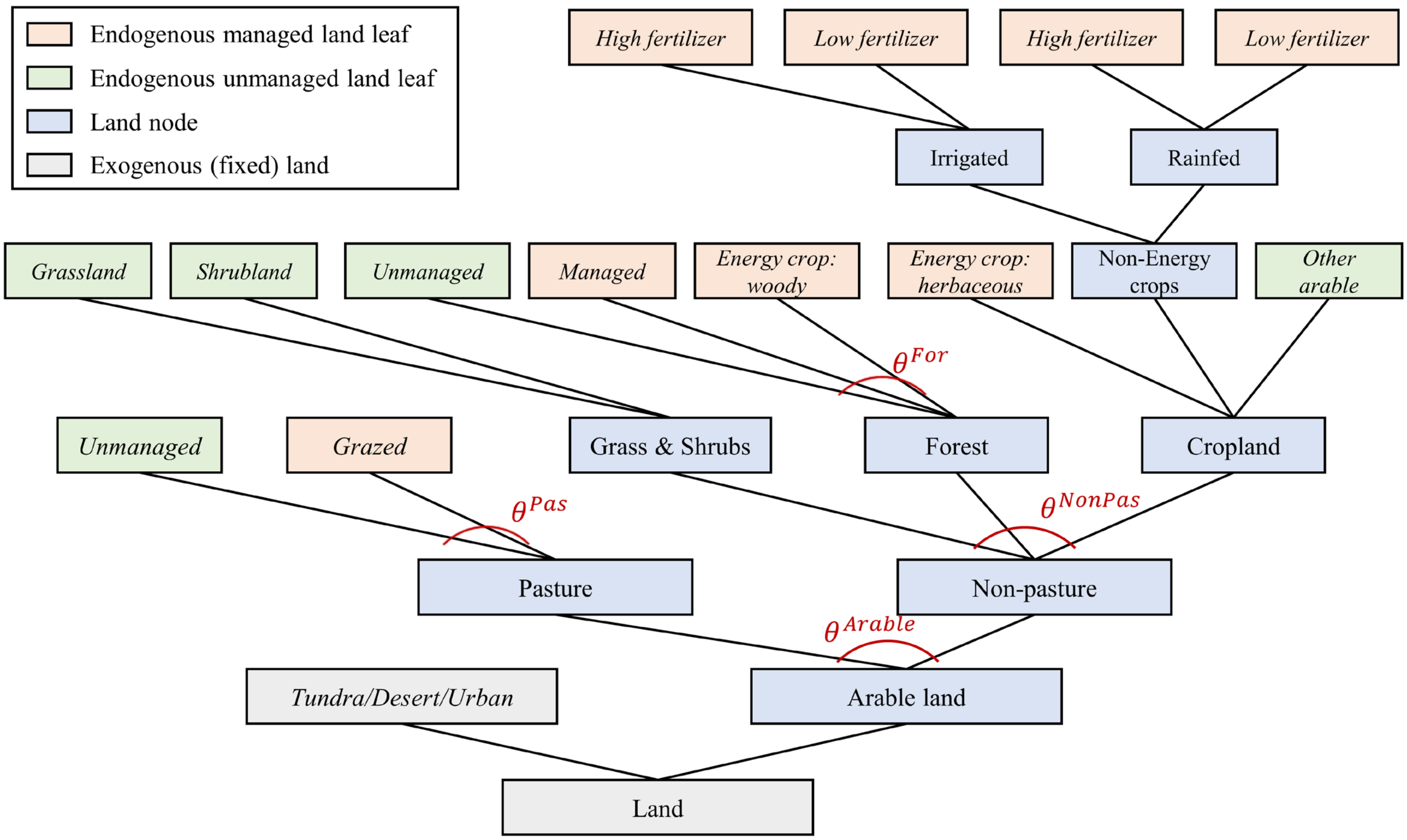

Fig. S6 | Land nesting structure in GCAM. The set of key logit exponent parameters used in this study is $\theta^{Arable} = 1.75$, $\theta^{Pas} = 2$, $\theta^{NonPas} = 0.75$, and $\theta^{For} = 1.25$. Note that managed (grazed) pasture is used in the Beef, Dairy, and Sheep & Goat sectors in GCAM. In addition, like non-energy crops, energy crop production technologies in GCAM are also differentiated by irrigation and fertilizer use. The other arable land corresponds to "land with temporary fallow" in FAOSTAT land data. Source: adapted based on Fig. S16 in Zhao et al. (2021)[16].

S2.3. Land rental profit and shadow profit

Agricultural production is modeled using the Leontief production function, and endogenous yield responses in crop production are realized via technology switching, e.g., fertilizer and irrigation options. Given the profit-maximizing agricultural producers and constant-return-to-scale (CRTS) production functions, the rental profit, r_i, can be derived for managed land, i.e., land with economic activities (**Eq. S2**).

$$r_i = (p_i - NLC_i) \cdot g_i \quad \text{(S2)}$$

Note that p_i is market prices, g_i is yield, and NLC_i is nonland costs (e.g., water, fertilizer, and others) per unit of output. And the shadow profit of unmanaged land is determined by the derived rental profit of managed land in base data. The production of forestry products in GCAM also uses the Leontief production function with the productivity coefficients connecting the primary roundwood production and managed forest land cover. Forestry data from FAOSTAT is used, and the cost share data from the GTAP v10 database is used. The forestry rental profit can also be calculated using **Eq. S2**.

There is no observed rental profit for unmanaged land since it is not currently being used for economic activities. However, the shadow price of unmanaged land, e.g., implying a marginal cost of land conversion or

an ecosystem service value, is an important factor determining the supply curve of unmanaged land. GCAM relies on a land dataset compiled by the Center for Sustainability and the Global Environment (SAGE) and the Global Trade and Analysis Project (GTAP)[40] for estimating the shadow price of unmanaged land. It is calculated as the average land rental profit across managed land in a region (water basin). For example, the global mean value is $140 per ha. In contrast, this value calculated using GCAM data would be about 30% higher ($190 per ha). In this study, we update the unmanaged land value to use the ones directly computed based on GCAM data since it is more consistent and avoids potential aggregation uncertainties in processing SAGE-GTAP data. In the new data, the median value across all regions is $143 per ha, and the 5th to 95th percentile range is $38 to $475 per ha. This update has a more significant impact on the distribution's shape and tails. In general, unmanaged land supply (e.g., afforestation/reforestation and deforestation) becomes less sensitive to land policies due to higher overall shadow prices of unmanaged land.

S2.4. Land system mitigation policy

The land system mitigation policy in GCAM is implemented as a carbon rent, i.e., land subsidy, to credit landowners for holding carbon stocks. The approach is consistent with the nested logit land allocation method used in GCAM. As shown in **Sections S2.2** and **S2.3**, land allocation is initially calibrated to historical data, and the relative land use (or land share) is responsive to relative land rental profit. With land system mitigation policies, carbon stored in land will be valued. That is, the land rental profit is increased to r_i' when factoring in an annualized land carbon storage rent, d_i (**Eq. S3**). And d_i is a product of the social discount rate (η) and the net present value (NPV) of carbon storage in both vegetation (NPV_i^{veg}) and soil (NPV_i^{soil}) (**Eq. S4**). In our study, $\eta = 3\%$, which is consistent with the Hotelling rate used to determine the carbon price escalation rate.

$$r_i' = r_i + d_i \tag{S3}$$

$$d_i = \eta \cdot (NPV_i^{veg} + NPV_i^{soil}) \tag{S4}$$

GCAM specifies carbon density at the equilibrium state, i.e., the maximum potentially achievable carbon density for a given land leaf, for vegetation carbon density (CD_i^{veg}) and soil carbon density (CD_i^{soil}). However, accounting for the dynamics of land carbon changes is essential, given the nonlinear nature of plant growth and soil carbon change. In GCAM, when the land area expands, the vegetation carbon growth follows a sigmoid function of the "mature-year" that is region- and land-specific[i], while vegetation carbon is released immediately (within a 5-year model step) for decreasing area. For both increases and decreases, the soil carbon changes follow an exponential function with a half-life implied by the regional "soil-time."

Thus, for vegetation carbon, the NPV is calculated as an integral of the discounted future carbon flow valued at the land system carbon prices (β^{Land}), as shown in **Eq. S5**, whereas $f^{sigmoid}(\cdot)$ is a sigmoid function and μ_t are discount factors calculated with a 10% private discount rate. The corresponding NPV equation for soil carbon is presented in **Eq. S6**, whereas $f^{exponential}(\cdot)$ is an exponential function and MCD^{soil} is a threshold of a minimum soil carbon density. That is, only the net soil carbon density ($CD_i^{soil} - MCD^{soil}$) is valued for soil carbon to reflect carbon storage that is additional. In addition, we set $MCD^{soil} = CD_{cropland}^{soil}$ in our study.

[i] For the mature-year of dedicated energy crops, 5-year and 8-year are used for herbaceous and woody biomass crops, respectively, in the version of GCAM used in this study.

$$NPV_i^{veg} = \int_{t=0}^{\text{MatureYear}_i} \left[\beta^{Land} \cdot CD_i^{veg} \cdot f^{sigmoid}(\text{MatureYear}_i)_t \cdot \mu_t \right] \quad \text{(S5)}$$

$$NPV_i^{soil} = \int_{t=0}^{\text{SoilTime}_i} \left[\beta^{Land} \cdot \left(CD_i^{soil} - MCD^{soil} \right) \cdot f^{exponential}(\text{SoilTime})_t \cdot \mu_t \right] \quad \text{(S6)}$$

The detailed land carbon data and parameters can be found in the open-source R package, *gcamdata* (e.g., */inst/extdata/aglu/LDS/ and /inst/extdata/aglu/Various_CarbonData_LTsage.csv*). Our approach to implementing a land carbon rent policy is generally consistent with the theoretical studies that focused on pricing forest carbon[41–43]. The approach allows systemically valuing carbon in all land types using the same carbon prices and the land carbon price (β^{Land}) can be linked to carbon prices in the Energy and Industrial Processes sectors.

When land carbon price increases, relative rental profits are affected so that landowners are incentivized to convert low-carbon-density land to relatively higher-carbon-density land. In practice, the effectiveness or responsiveness of land system mitigation policies depends on several key factors: (1) land logit exponents and calibrated logit share-weights, (2) land carbon density accounting method and related parameters (e.g., discount rate, timing parameters, etc.), and (3) initial land allocation data and implied rental profits. For example, when the land logit exponent is small or land availability is limited (e.g., low accessibility), implying a relatively inelastic land supply response, a higher carbon price is required to encourage the same land conversion (e.g., afforestation). Also, land policies tend to be more effective when the carbon densities are more different across land categories since relative rental profits would thus be more sensitive to carbon prices. If all land categories have a similar carbon storage ability, then land mitigation policy would have little impact on carbon storage. Overall, rental profits, as calculated based on production technology specifications and market information, connect land competition and the land mitigation policy to other market-mediated responses.

S2.5. Land carbon density

Recently, in GCAM v6, the land carbon density was updated based on data mainly from the SoilGrid database[44] (soil organic carbon) and Spawn et al. (2020)[45] (vegetation carbon). When aggregating carbon density data from the grids to GCAM water basins, the third quantile value is used to represent an equilibrium state density. Also, soil carbon density is for topsoil (0-30 cm). The data for key land categories are shown in **Figs. S7–S8**. Note that the data for forest and pasture corresponds to unmanaged land in GCAM. It is assumed that the vegetation carbon density of managed forest and pasture is 50% of their corresponding unmanaged land in a region. And the soil carbon density in managed land is assumed to be 13% and 20% lower than unmanaged land for forest and pasture, respectively. Note that soil carbon density in **Fig. S7** shows data for annual cropland, while soil carbon density in tree crops and other arable land is assumed to be 20% higher than annual cropland[46]. In addition, by default, GCAM assumes that the soil carbon density of dedicated energy crops is the same as that of unmanaged pasture in a given region. For vegetation carbon of cropland, **Fig. S7** (right panel) shows the data assumed uniformly for “Other Arable Land” in GCAM, which is 3 MgC per ha. In GCAM, vegetation carbon by crops is computed based on crop yield and other key crop-specific factors, e.g., root-to-shoot ratio, carbon content, timing factor, etc. As discussed in **Section S2.4**, the difference in land carbon density across land is more important in determining the effectiveness of the land mitigation policy, e.g., **Figs. S9**.

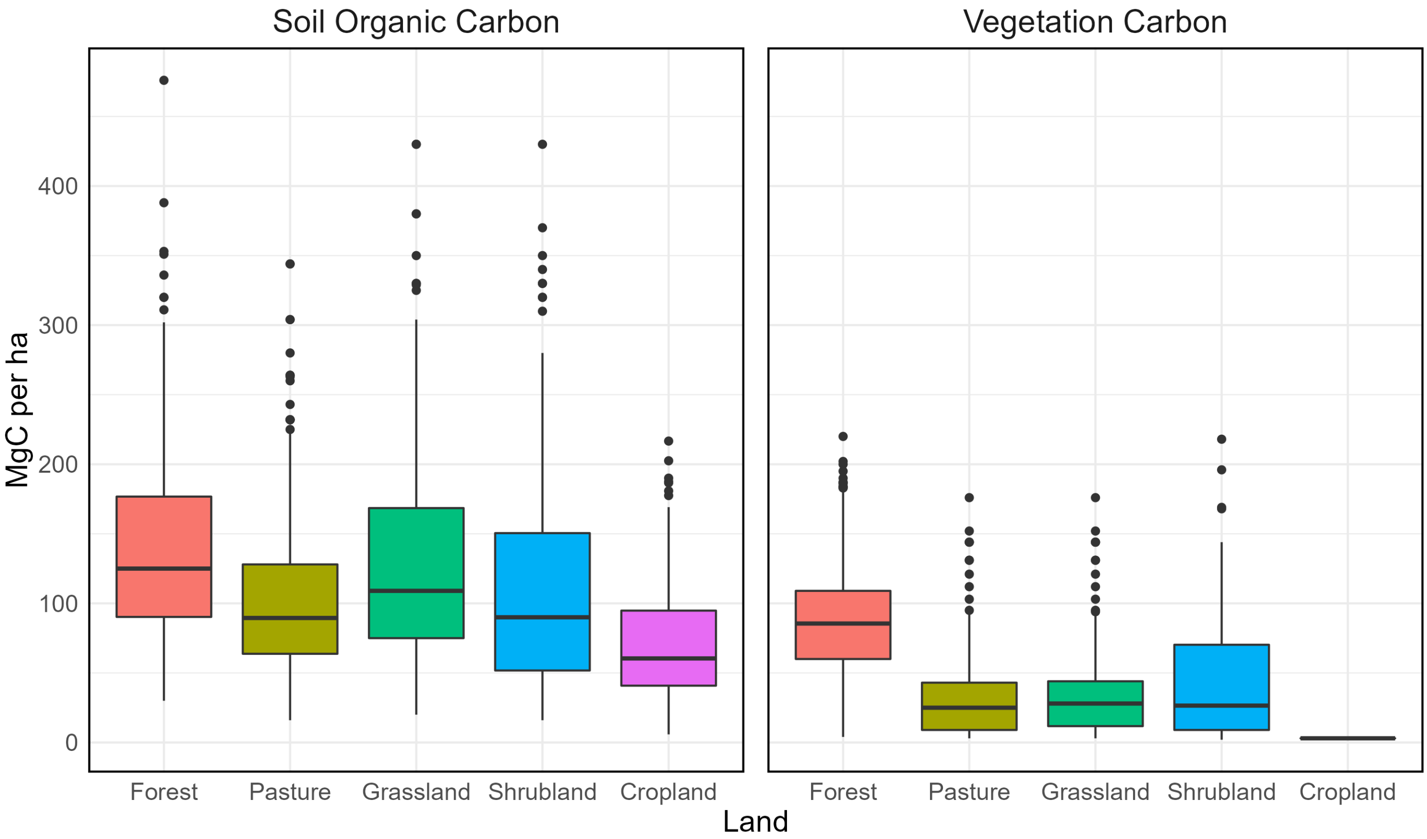

Fig. S7 | Land carbon density distributions. The distributions of land carbon density across regions by land types are shown for soil organic carbon (left) and vegetation carbon (right). The boxplots show the median values (line), the interquartile ranges (IRQ; boxes), the 1.5 x IRQ ranges (whiskers), and points outside 1.5 x IRQ ranges (black points) of the default GCAM v6 land carbon density data. Note that Forest and Pasture correspond to the unmanaged forest and unmanaged pasture in GCAM, and the soil organic carbon of cropland is for annual crops. Note that the median value of the total (soil organic carbon & vegetation carbon) land carbon density is 224 MgC per ha for forest with an IRQ of [164, 284] MgC per ha. Source: gcamdata in GCAM v6.

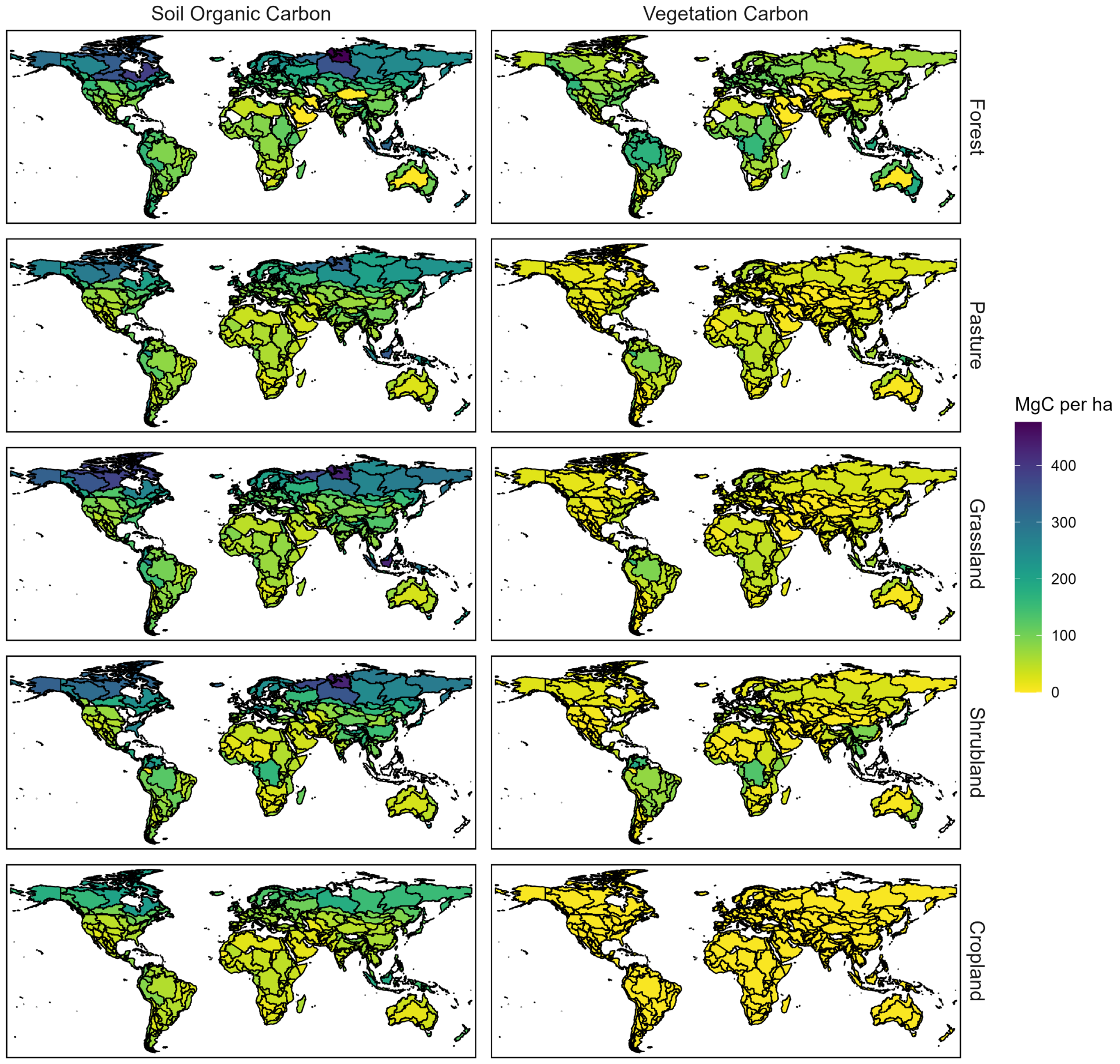

Fig. S8 | Land carbon density maps. Global maps of the default GCAM land carbon density data at the water-economy regions for key land categories by soil organic carbon and vegetation carbon.

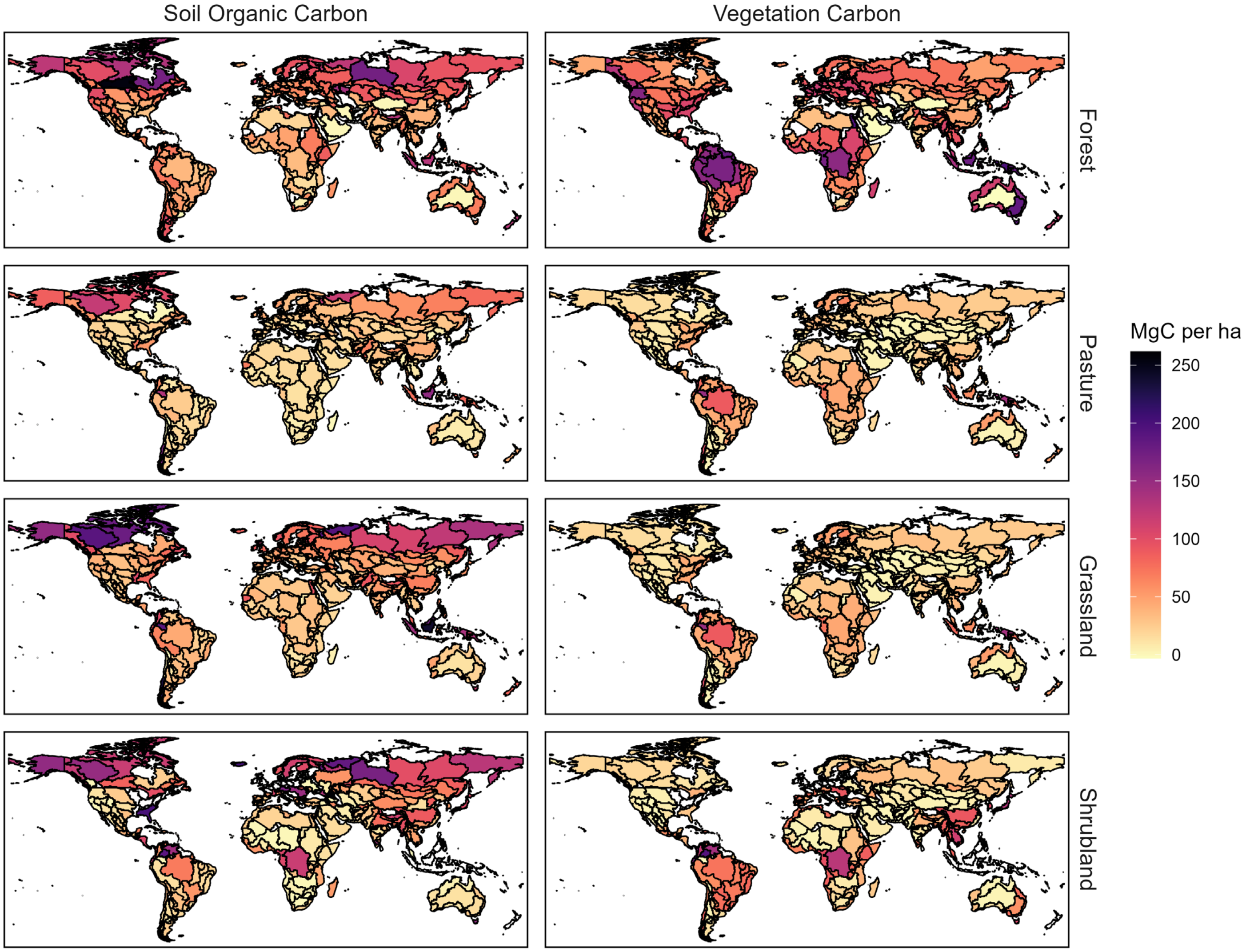

Fig. S9 | Difference of land carbon density relative to cropland. Land carbon density difference between key land categories (Forest, Pasture, Grassland, and Shrubland) and Cropland.

S2.6. Agricultural demand responses

In GCAM, a nesting structure (**Fig. S10**) is used to aggregate food from different sources (measured by calories). At the top level (Staples vs. Non-Staples), the approach developed by Edmonds et al. (2017)[14] is used to specify how own- and cross-price elasticates and income elasticities change endogenously with income. In other words, the per capita food calorie consumption is responsive to price & income and substitution is allowed between staple and non-staple food calories, as implied by the parameters specified. Food calories, or dietary energy available, were derived based on food demand (in tonnes) and the conversion factors were compiled based on FAOSTAT data using the R package *gcamfaostat*[47].

Currently in GCAM v6, food consumption at the lower nests does not allow substitutions, despite a nested logit structure (i.e., zero logit exponent parameters). That is, calorie consumption share across commodity sources is fixed under Staples or Non-Staples. However, in this study, we allow a more flexible food demand substitution to reflect the endogenous price-induced dietary change. In particular, we set the logit exponent

parameter to -1 in all nests (except the top nest) so that it is close to a Cobb-Douglas utility function (e.g., fixed expenditure share)[ii]. In mitigation scenarios, GCAM captures the price transmission from the carbon market to the food market. A higher elasticity of substitution in food consumption allows consumers to mediate the food price impacts via more responsive price-induced dietary changes.

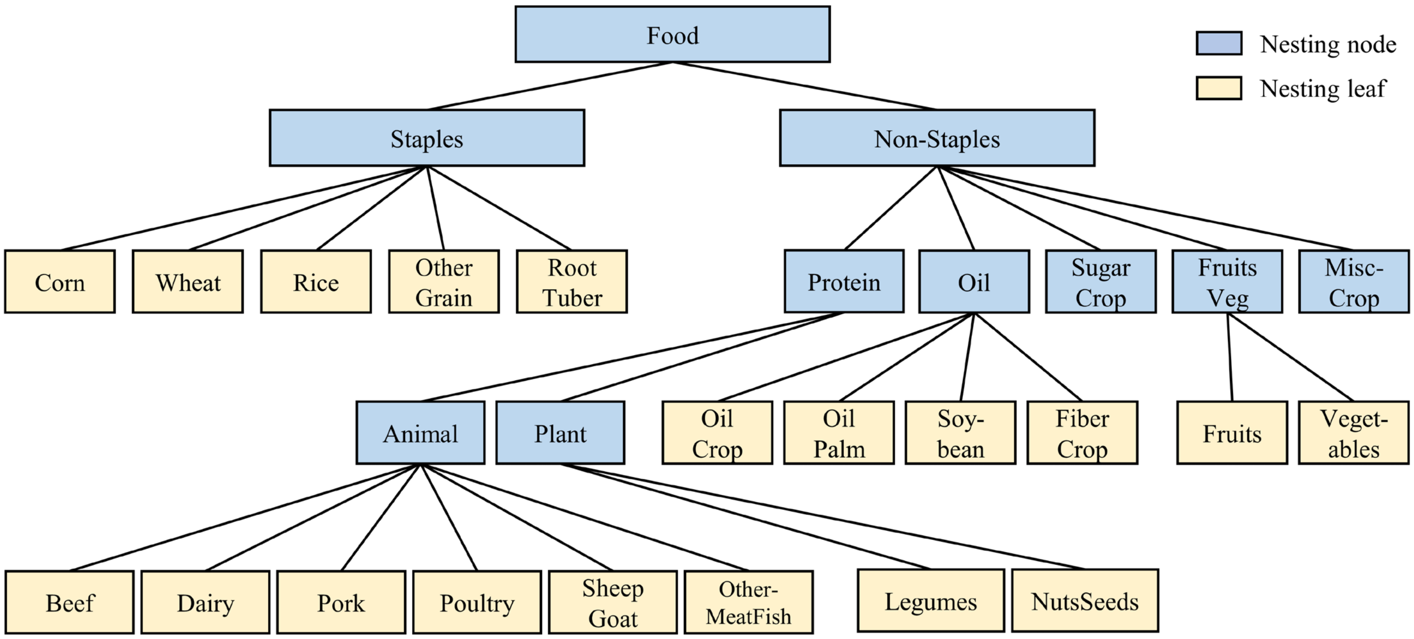

Fig. S10 | Food demand nesting structure in GCAM v6. See **Table. S2** for the GCAM commodity mapping.

S2.7. Residual biomass supply

The supply curves for agricultural and forestry residual biomass in GCAM were developed based on Gregg and Smith (2010)[49]. The maximum residual biomass energy availability is derived based on a set of agricultural product-specific attributes, e.g., harvest index, root-to-shoot ratio, water content, energy content, and retention requirement for erosion control. And the maximum availability is endogenously linked to agricultural and forestry production in GCAM, and the share of the availability is responsive to biomass prices. The recent model intercomparison in Hanssen et al. (2020)[50] indicated that GCAM's residual biomass production is at the high end compared with the literature. For this study, we updated the key parameters and assumptions (e.g., *gcamdata/inst/extdata/aglu/Various_ag_resbio_data.csv*). We also added a 10% requirement for other uses (e.g., animal bedding)[51] and considered 5% – 20% dry matter loss[52,53] when determining the maximum residual biomass energy available for crops. In addition, the supply curve is recalibrated based on the information provided by Hanssen et al. (2020) (Figure S8). In particular, a lower supply price at the maximum availability is used, i.e., decreased from 10 $US\$_{1975}$/GJ to about 6 $US\$_{1975}$/GJ. As a result, the residual biomass supply curve shifts moderately towards the bottom left and is more consistent with the literature. It is worth noting that the

[ii] Note that similar modifications enabling greater flexibility in substitution on the demand side have been tested and implemented in recent studies, e.g., a logit exponent of -0.25 was used in Zhao et al. (2021)[16] and a Cobb-Douglas utility function was tested in Zhao et al. (2021)[48].

supply curve for forestry residues (both primary and secondary) is tied to forestry products which are produced using managed forest land, not unmanaged forest land.

S2.8. Bioenergy carbon flows, price transmission, and budget limits on crediting BECCS

Land-based CDR measures rely on different processes for carbon removal. A/R involves expanding forests to increase carbon sequestration in vegetation and soil. BECCS, on the other hand, utilizes advanced energy system technologies to convert lignocellulosic biomass into modern energy carriers while also capturing the biogenic carbon and storing it underground in a geologic formation[54].

GCAM traces the energy and emission flows and their corresponding monetary values. The flow chart for advanced bioenergy supply and demand is shown in **Fig. S11**. The primary bioenergy supply includes purpose-grown bioenergy crops, residues, and municipal solid waste (MSW). A logit-based Armington framework is used for connecting future regional supply to regional demand, and a weak market integration trend is assumed. Primary biomass is consumed in different sectors, i.e., refining, electricity, gas, hydrogen, and end-use (final energy). The outputs from intermediate sectors are also consumed by the end-use sectors in GCAM, e.g., building, transportation, and other industries. In GCAM energy and energy service systems, nested logit structures are used to represent market competitions across subsectors and technologies, including the technologies with CCS options. Our study relies on the default assumptions of CCS cost and removal fractions in GCAM[iii]. The levelized non-energy costs, e.g., carbon capture, compression, transport, and storage, are derived based on Dooley and Dahowski (2009)[55]. In addition, GCAM assumes that all biomass-to-H_2 technologies will be equipped with CCS while biomass gasification is not equipped with CCS[iv]. GCAM recently broke out more detailed industries and added CCS options in the alumina and chemical sectors, but they play an insignificant role in BECCS deployment compared to refining and electricity.

A comprehensive representation of the intersectoral linkages is critical for emission accounting and mitigation policy implementation[56]. Direct agricultural inputs (e.g., fertilizer and irrigation) emissions are explicitly traced on the supply side for advanced bioenergy pathways. On the demand side, carbon in biogenic carbon (i.e., 23 gC per MJ) in primary biomass, carbon in other energy inputs in energy conversion, and combustion emissions are explicitly traced. For energy flows with intersectoral linkages, carbon policies value the corresponding emissions endogenously. For example, with carbon prices, fertilizer market prices would be higher, driven by the higher energy costs induced by carbon taxes in fertilizer production. Thus, the prices of purpose-grown bioenergy crops would be higher. However, the sectors that utilize CCS technologies receive carbon subsidies due to the negative emissions. It is important to note that GCAM v6 does not account for emissions from farming or transportation and distribution. In our study, we attribute 15% of the biogenic carbon to account for these emissions, based on life-cycle analysis[v], i.e., Wang et al. (2012)[59] and Prussi et al. (2021)[60]. The additional emission attribution avoids the over-crediting of bioenergy or BECCS under mitigation policies in GCAM.

[iii] Note that GCAM does not explicitly model the detailed supply chain of the CCS "service" and only model representative regional suppliers (i.e., with no spatial details but representing the mean costs and technological specifications).

[iv] In GCAM mitigation scenarios, biomass-to-H_2 technologies are typically deployed in later periods of the century when carbon prices are sufficiently high. Biomass gasification plays a relatively insignificant role and tends to be crowding out over time.

[v] Note that Second-generation (2G) bioenergy generally has a smaller life-cycle emission than first-generation (1G) biofuels. We also attribute 55% of the biogenic carbon to account for emissions along 1G biofuels supply chain that were not explicitly traced in GCAM, based on Chen et al. (2018)[57] and Lee et al. (2021)[58].

Under stringent climate mitigation policies, GCAM may rely heavily on BECCS, resulting in significant net negative emissions at the end of the century. GCAM addressed the feasibility of large-scale net negative emissions by implementing a limit on the percent of GDP that would be allowed to be spent on subsidizing biomass for net negative emissions. The feature has been included in GCAM v5[35], and a budget limit of 1% GDP was assumed. Recently in GCAM v6, the net negative emission in the budget constraint for BECCS is updated to biogenic carbon for the convenience of policy implementation and value tracing. Including a limit on the negative emission technology budget (e.g., % of GDP) implicitly represents a macroeconomic linkage that was missing in the model, and it allows differentiating carbon prices across sectors. In this study, for exploring the trade-offs between land-based mitigation measures, we set a relatively higher budget limit[vi], i.e., 2% of GDP, so that the constraint is not distorting the responses across the studied scenarios. Stronger limits on BECCS are considered in the scenario design explicitly.

It is also important to note that constraining the deployment of BECCS by using a budget limit (% of GDP) effectively reduces the shadow price of carbon in BECCS. Even though it further complicates the carbon pricing in EIP sectors, our approach permits tracing the value flow and shadow price of carbon across sectors. The same method is used in implementing the “Low-Bioenergy” scenarios in our study. As expected, in most studied scenarios, the budget constraint will not bind until later periods when net-zero carbon emissions are reached (see shadow prices of carbon in **Fig. S13**). Using the BECCS budget limit in our study may also enhance compliance with the Hotelling rule, as demonstrated in Strefler et al. (2021)[61] with alternative Hotelling paths.

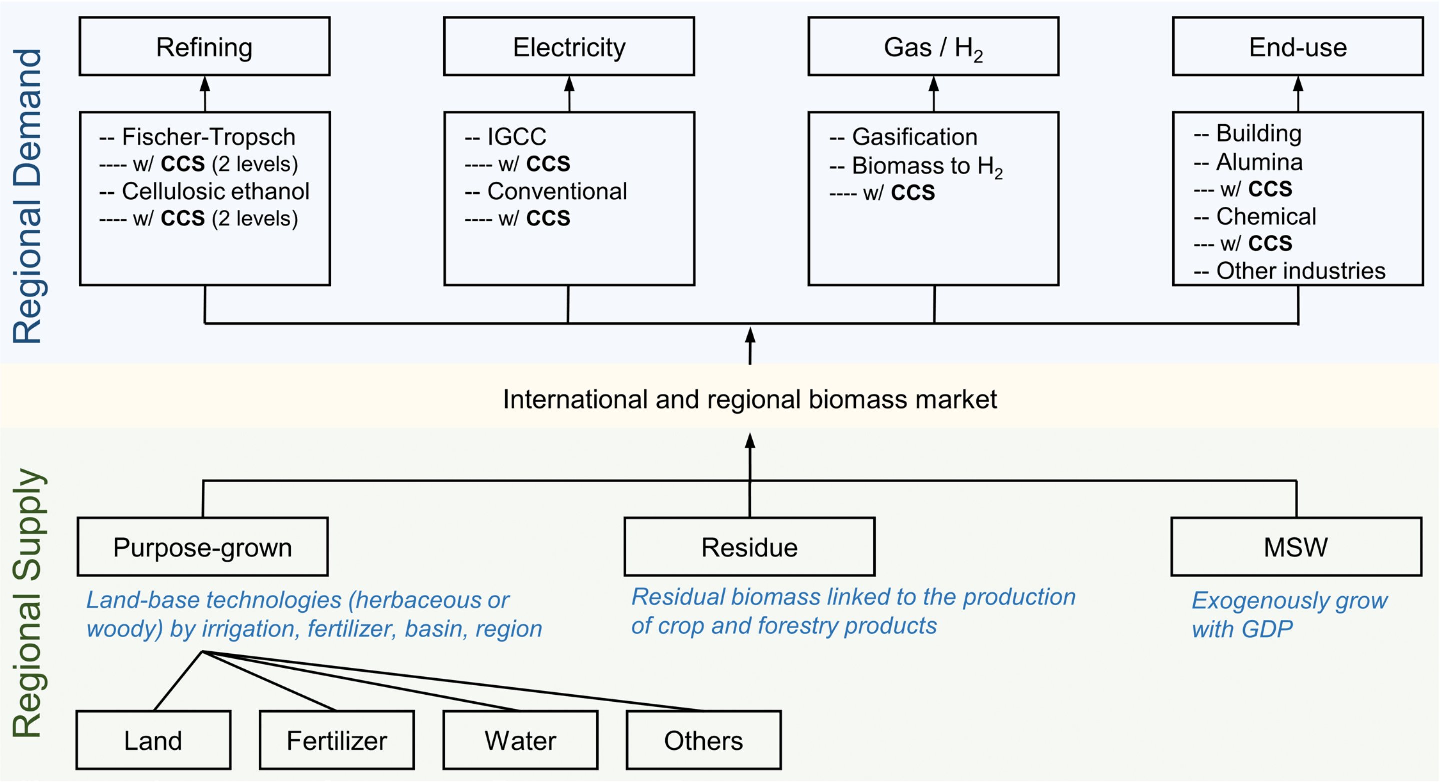

[vi] BECCS is the only sector that is affected by the budget limit in this study, which is the default assumption in GCAM v6. Note that if the direct air capture with carbon storage (DACCS) option in the version of GCAM is turned on, it would be included in the budget limit as well.

Fig. S11 | Schematic of biomass flows in GCAM v6. IGCC stands for integrated gasification combined cycle. Note that w / CCS indicates the technology has the option of using carbon capture and sequestration (CCS) technologies under mitigation policies.

S3. Supplementary results and discussion

S3.1. GCAM reference scenario

A summary of key results in the reference projection is provided in **Fig. S12**. When no mitigation policy is included, driven by the higher future consumptions of fossil fuels and non-CO_2 GHG intensive products, the global total GHG emissions increase by about 70% from 50.5 $GtCO_2$-eq yr^{-1} (40.3 CO_2 and 10.2 non-CO_2 GHG) in 2015 to 85.5 $GtCO_2$-eq yr^{-1} (65.2 CO_2 and 20.3 non-CO_2 GHG) in 2100 (**Figs. S12A** and **S12B**). The cumulative carbon emission in 2020 – 2100 is 4381 $GtCO_2$, including 111 $GtCO_2$ from LULUCF, and the cumulative non-CO_2 GHG emission is 1317 $GtCO_2$-eq.

The total cropland increases by 360 Mha (~23%) from 1584 Mha in 2015 to 1944 Mha in 2100. More than half of the increase is driven by purpose-grown energy crops (+176 Mha), and staple crops increased by 71 Mha (from 629 Mha to 700 Mha). Pasture is the primary land source (-211 Mha), followed by other natural lands (i.e., -132 Mha in grassland and shrubland). The net total forest area decreased by 16 Mha, with a 199 Mha reduction in the natural forest but a 186 Mha increase in managed forest (**Figs. S12C**). The regional heterogeneity in future socioeconomic and productivity changes lead to different regional LUC and LULUCF emission patterns (**Figs. S12D**), consistent with discussions in Zhao et al. (2022)[15].

For primary bioenergy (second generation), nonland-based biomass supply (agricultural and forestry residues and MSW) increases from 26 EJ yr^{-1} to 40 EJ yr^{-1} in 2100, while purpose-grown biomass, introduced in 2025, supplies 36 EJ yr^{-1} in 2100. Following the historical trend, biomass is still mainly used in end-use sectors (47 EJ in 2100), e.g., buildings and industries, in reference projection. In addition, no BECCS is being deployed in the reference scenario since the CCS technology deployment relies on carbon policy incentives. Notably, bioenergy already used in the reference scenario can be reallocated to BECCS technologies in mitigation pathways, as also highlighted in Bauer et al. (2020)[62]. The first-generation bioenergy consumption changes over time are relatively moderate compared with the second-generation bioenergy (**Figs. S12E**). This is also the case in mitigation pathways as they compete for resource uses (e.g., land, water, etc.) and could substitute for consumption (refining).

Future agricultural price changes vary by sector (**Figs. S12F**), as there will be dietary changes (e.g., increasing meat consumption) and reallocations of global agricultural production due to income and substitution effects[14]. For staple crops, globally, the supply-side changes, e.g., productivity growth, technology adaptation, and trade responses, offset the demand-side drivers, and thus, the world prices are fairly stable (e.g., +1% by 2100). The price of livestock products has a relatively higher increase (e.g., +5% by 2100) due to the stronger future demand and the cost transmission from pastureland and feed crops.

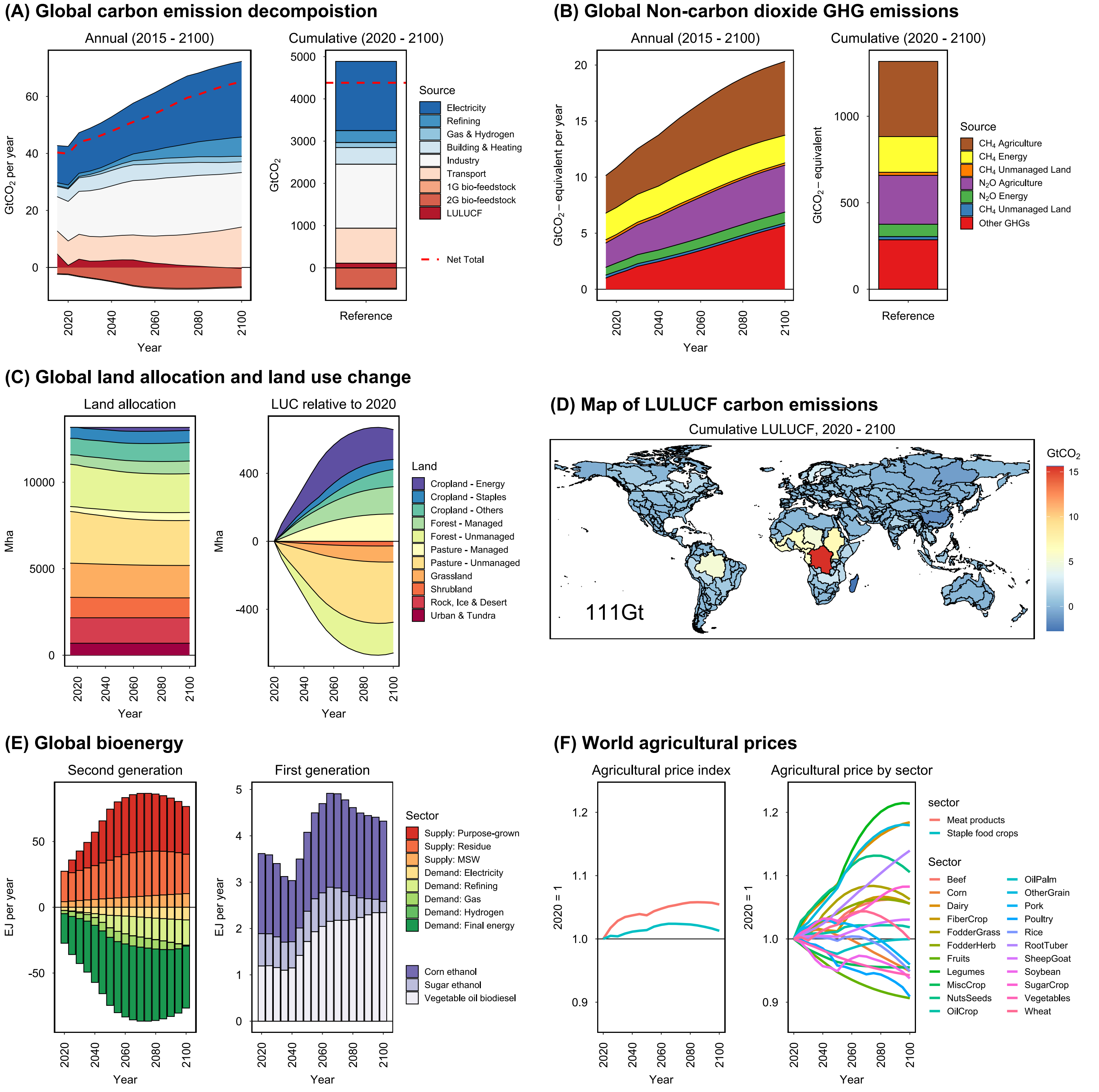

Fig. S12 | Summary of key results in the reference projection. Panel A displays a decomposition of global annual (left) and cumulative (right) carbon dioxide emissions. Panel B shows a decomposition of global annual (left) and cumulative (right) non-CO_2 emissions. Panel C presents the reference projections of global land allocation (left) and the land use change relative to 2020 (right). Panel D shows the map of the LULUCF carbon emissions with the net total value labeled (111 $GtCO_2$). Panel E shows the global primary bioenergy supply and demand for second-generation bioenergy (left) and the production of first-generation bioenergy (right). Panel F presents the agricultural price index by sectors, including the aggregated sectors (meat and staple food crops; left) and disaggregated GCAM sectors (right).

S3.2. Supplementary discussion of results in mitigation pathways

In the main paper, **Figs. 3** and **4** present a comparison of key subsets of the mitigation pathways in different scenario dimensions. The complete set of results for all pathways can be found in **Figs. S13 – S27**. This section aims to provide an overview of these mitigation pathway results. While all the result figures are thoroughly captioned and self-explanatory, this section also offers additional context to enhance clarity and discuss important implications.

Fig.2 in the main paper and **Fig. S13** (Panel A) present the shadow prices of carbon by sectors, which are the solution variables in the mitigation pathways (additional variables compared to the reference scenario). The data for generating **Fig. 2** are provided in **Tables S3** and **S4**. As expected, for scenarios with the same climate target, carbon prices in FFI sectors are negatively related to land-based carbon mitigations. That is, higher removals through land-based CDRs permit lower mitigation efforts in FFI sectors, resulting in lower FFI marginal cost of mitigation. In our study, where applicable, we report prices in constant 2010 US dollars, following the IPCC AR6 convention. **Fig. S13 (Panel B)** also displays the annual mitigation relative to the reference scenario. It is important to note that the combinations of carbon prices and mitigation represent a single point per year on the marginal abatement cost curve (MACC). In our study, we do not explore the welfare impacts or policy costs[9] due to the additional computational burden and the high uncertainty[vii] associated with such calculation[63]. However, future studies investigating such policy benefit-cost analyses need to recognize the sectoral differentiation in carbon prices. **Fig. 2** in the main paper also includes a comparison with AR6 results of carbon prices. It is worth noting that AR6 pathways may exhibit different shadow prices of carbon across sectors, but only carbon prices for a global carbon market were reported to the Scenario Database. The global carbon prices in AR6, by default, should correspond to the shadow price of carbon in the Fossil Fuels and Industry (FFI) sectors. It is also important to note that when land carbon is partially priced, or incentive limits are added to BECCS, FFI carbon prices cannot represent the economy-wide marginal cost of mitigation.

In **Fig. S14**, global carbon emissions are decomposed to trace detailed carbon removals, specifically BECCS by source and LULUCF. **Fig. S15** presents the default emission decomposition results in GCAM, which separate biogenic carbon, including negative values for first-generation (1G) and second-generation (2G) bio-feedstock[64], while positive emissions encompass both uncaptured biogenic carbon and fossil fuel carbon. Regional breakdowns are also provided in **Fig. S15** (Panel B). Furthermore, as described in the Method section of the main paper, similar to other IAMs, GCAM does not differentiate primary cellulosic bioenergy by feedstock source once it enters the energy system. This assumption implies that the market for second-generation (2G) primary bioenergy is integrated, and the products are homogeneous. For example, pelleted and processed biomass pellets derived from corn stover or miscanthus are likely of similar quality and economic value. Consequently, the carbon content of primary cellulosic bioenergy is assumed to be the same regardless of the feedstock source.

In general, the emission reductions observed in the results are a direct result of carbon policies (**Fig. S13**), and the responses across scenarios are consistent with sector-specific policies. The reference scenario, driven by historical global net deforestation trends, exhibits significant LULUCF emissions. Mitigation pathways without land policies tend to exacerbate deforestation, while stronger land policies help alleviate deforestation and encourage net afforestation. It is important to acknowledge that assuming the absence of land policies does not accurately reflect reality, as considerable efforts have been made to protect and preserve forests and

---

[vii] As a partial equilibrium model, GCAM employs an approach to calculate policy costs by running multiple iterations to determine a MACC and then computing the area under the curve.

natural areas. Initiatives such as Reducing Emissions from Deforestation and Forest Degradation (REDD+) established by the United Nations Framework Convention on Climate Change (UNFCCC)[65], the Bonn Challenge[viii], and zero-deforestation supply chain policies[66], have been implemented to combat deforestation and preserve nature areas. On the other hand, implementing 100% land carbon pricing could pose institutional challenges[67,68], such as emission accounting and policy enforcement and monitoring, and raise concerns about food security[ix]. Therefore, scenarios that lie between the absence of land carbon pricing and 100% land carbon pricing may provide a more realistic reflection of real-world policies. Exploring these intermediate scenarios is an important contribution to our study.

In addition, in **Fig. 4B** and **Fig. S14**, note that the 2025 increase in LULUCF emissions under A/R-Focused & 100%-LCP was consistent with scenarios with partial land carbon pricing in the scenario. This increase in LULUCF emissions was due to land expansion into other natural land. It is worth noting that since carbon policy implementation begins in 2025, carbon sequestration in A/R has just commenced and thus does not have a substantial impact on reducing emissions in the early years.

Fig. S16 illustrates the projections of non-CO_2 GHG emissions. For the 2°C pathways (n = 11), the mean (standard deviation) of the total cumulative non-CO_2 GHG emissions is 944 (24) GtCO_2-eq. Similarly, for the 1.5°C pathways (n = 4), the mean (standard deviation) is 921 (27) GtCO2-eq. Compared to the reference scenario (1317 GtCO_2-eq), the reductions in non-CO_2 GHG emissions, amounting to 28% in 2°C pathways and 30% in 1.5°C pathways, are primarily driven by demand responses and technological improvements induced by carbon pricing. The improvements are implied by the technology-level marginal abatement cost curves specified for non-CO_2 GHG species in GCAM. Note that the differences observed across pathways for each climate target are relatively small compared to the uncertain range in the transient climate response to cumulative emissions of carbon dioxide (TCRE). Therefore, the variations in non-CO_2 GHG emissions are expected to have a minimal impact on our cumulative carbon dioxide emission targeting approach. However, the decomposed results highlight that agricultural CH_4 emissions are the primary source of variation across the studied pathways. Stronger land carbon pricing plays a key role in reducing agricultural CH_4 emissions, particularly through its larger impacts on cropland and pasture, which leads to a relative decrease in rice cultivation and beef production.

Figs. S17-S21 depict the primary bioenergy and biogenic carbon flows from supply sectors to demand sectors, following the structure shown in **Fig. S11**. Across the studied scenarios, the average supply of primary bioenergy ranges from 66 EJ yr^{-1} (Low-Bioenergy & 100%-LCP) to 166 EJ yr^{-1} (1.5 °C & No-LCP), while the supply of purpose-grown primary bioenergy ranges from 22 EJ yr^{-1} (Low-Bioenergy & 100%-LCP) to 105 EJ yr^{-1} (1.5 °C & No-LCP). The decomposition analysis emphasizes the significance of biomass derived from residues and municipal solid waste (MSW) (**Figs. S17-S18**) and the transition in biomass use from final energy sectors to sectors with high carbon capture potential (**Figs. S19-S21**). In addition, residual biomass from both crop and forestry does not vary significantly across scenarios as they are constrained by the supply of crop and forestry products (**Table S5**). In particular, forest products are produced from managed forest land. And managed forest land, associated with forestry product demand and economic growth (with price and income elasticities), exhibits relatively greater stability compared to unmanaged forest land (with relatively higher land carbon density), which is relatively more responsive to land carbon policies.

[viii] https://www.bonnchallenge.org/

[ix] Agricultural GHG emissions have not been a primary focus in the existing regulation schemes, e.g., they are not currently covered in the emission trading system (ETS) in the EU and the New Zealand.

In scenarios other than Low-Bioenergy, purpose-grown bioenergy experiences faster growth compared to residue and MSW, as energy crop yields increase over time (**Fig. S22**), and the supply of residues and MSW may approach their physical limits sooner. On the demand side, the majority of primary biomass is used in the electricity, refining, and hydrogen sectors by 2100. The mean share of primary bioenergy used in sectors combined with CCS technologies ranges from 61% (Low-Bioenergy & 100%-LCP) to 88% (1.5 °C & No-LCP), and the share of the CCS efficiency (share of carbon captured in biogenic carbon in the sector) ranges from 65% (A/R-Focused & 100%-LCP) to 76% (1.5 °C & No-LCP). The reliance on purpose-grown biomass, sectoral transitions, and carbon capture efficiency are all influenced by carbon prices, leading to market-mediated responses with varying dynamics across scenarios. Our model projects these responses endogenously.

Fig. S22 shows the yield projections for purpose-grown energy crops. The yield growth is much higher than the exogenous biophysical yield growth specified (**Fig. S5**), implying a strong price-induced land intensification. The land use and land use change projections are provided in **Figs. S23-24**, with the net LULUCF carbon emissions maps shown in **Fig. S25**. Across the studied scenarios, the mean (2020 – 2100) purpose-grown energy cropland increase ranges from 110 Mha (Low-Bioenergy & 100%-LCP) to 519 Mha (1.5 °C & No-LCP), and the forest change ranges from -151 Mha (1.5 °C & No-LCP) to 647 Mha (A/R-Focused & 100%-LCP). Other nature land (grassland and shrubland) also experiences a large variation, ranging from -298 Mha (A/R-Focused & 100%-LCP) to 45 Mha (Low-Bioenergy & 100%-LCP). These changes reflect the influence of land competition and comprehensive land policies. Regional patterns of land use change are generally consistent, although the magnitude of responsiveness to land policies may differ. Overall, stronger land carbon policies incentivize regions to allocate more land for carbon storage, promoting more efficient land carbon removal.

Due to the dynamics and the market-mediated responses across sectors, space, and time, the policy-driven trade-offs between land-based CDR options have implications on carbon prices, agricultural prices, resource use efficiency, and other sustainability metrics. In **Figs. 3 & 4**, we present the results connecting land-based CDRs to their corresponding land use, followed by illustrating the land carbon removal intensity and its corresponding decomposition. It is important to note that the removal intensity values in **Fig 3E & 3F** (**Fig 4E & 4F**) were computed based on the data presented in **Figs. 3B & 3D** (**Figs. 4B & 4D**). Similarly, the relationship between **Fig. S14** and **Fig. S24** implies the land carbon removal intensity shown in **Figs. S26-S27**. Furthermore, we compared the land removal intensity from our results with AR6 pathways in **Fig. S28-S30**. **Table S6** also summarizes the derivation of land removal intensity in recent sectoral studies in comparison with our results. For additional discussion of these results, please refer to **Section S3.3**.

Fig. S31 presents the distribution of agricultural price impacts (relative to the reference scenario) across sector-region combinations in GCAM. The overall pattern is similar to what was discussed in the main **Fig. 6A**, albeit with wider ranges observed. In **Fig. S32**, the relationship between the shadow price of carbon and land use impacts is visualized. It provides a preliminary understanding of the acreage responses, both own-price and cross-price, in relation to carbon prices. As expected, the relationship is positive, and stronger connections are observed between forest carbon prices and forest land use change, as well as between BECCS carbon prices and energy cropland change. Moreover, longer-term responses (e.g., 2100) tend to be more uncertain compared to near-term responses (e.g., 2050). These results underscore the significance of sector-specific and year-specific carbon prices when exploring their implications on land use and agricultural prices.

Fig. S33 complements the main **Fig. 6D** and provides additional insights into the relationship between agricultural prices and cropland impacts. The future use of cropland is sensitive to land-based mitigation policies. For instance, in the Main & 100%-LCP scenario, global cropland area decreases by approximately 21% (400 Mha), while staple cropland area decreases by 24% (or 170 Mha) compared to the reference

scenario by 2100. However, yield intensifications (**Fig. S34**) and demand-side adaptations (e.g., reduced crop consumption for feed and other purposes; **Fig. S35**) play crucial roles in mediating the production and price impacts. In addition, these responses are more important when land mitigation policies are stronger. **Table S7** presents the regression results for the agricultural price models, with detailed descriptions provided in the table caption and the main paper. Furthermore, the world average dietary energy available is about 2960 kilocalories per capita per day (kcal/ca/d) in 2020, and it is projected to grow by 27% to 3770 kcal/ca/d by the end of the century in the reference scenario (**Fig. S36**). Under the mitigation runs, the dietary energy supply could decrease moderately by 12 (Low-Bioenergy & No-LCP) to 85 (Low-Bioenergy & 100%-LCP) kcal/ca/d compared to the reference projection. Regional impacts could be larger, particularly for regions that are more sensitive to land-based mitigation policies, e.g., AFRICA (-105 kcal/ca/d in Low-Bioenergy & 100%-LCP and -90 kcal/ca/d in 1.5C & 100%-LCP) and LATIN_AM (-105 kcal/ca/d in Low-Bioenergy & 100%-LCP).

S3.3. Unraveling the complexity of land carbon removal intensity

Similar to the importance of "crop yield" in crop production, the concept of “carbon yield” or land carbon removal intensity plays a crucial role in comprehending and comparing land-based CDR measures. This efficiency metric is commonly employed in sectoral studies that typically focus on a specific land-based CDR measure. A recent seminal study by Roe et al. (2021)[69] extensively examined this efficiency, known as “mitigation density,” across various land-based CDR measures using data adapted from sectoral studies. In our study, we also calculate land carbon removal intensity to evaluate the outcomes of IAMs. Our approach is described in **Methods** in the main paper.

Figs. S26-S27 illustrate the results of our scenarios using two methods of LULUCF removal intensity. The LULUCF in IAM studies accounts for land use change carbon emissions beyond forest land changes, even though forest change is a main driver of LULUCF changes (**Fig. S3**). For example, changes in other natural land, e.g., grassland and shrubland, also play key roles in LULUCF accounting. In our No%-LCP and 10%-LCP scenarios, where human land expansion into grassland and shrubland contributes to LULUCF emissions in addition to net global deforestation, the land removal intensity for LULUCF becomes more uncertain using our default approach (**Fig. S26**). However, when other natural land is included, the removal intensity becomes more comparable across scenarios, as shown in the alternative approach (**Fig. S26**). Our analysis focuses on the default approach, as it aligns better with the existing literature and allows for comparisons with available results in AR6 pathways. Moreover, since the metric aims to measure net removal intensity, the results are more meaningful in the case of scenarios with net LULUCF removal (e.g., net afforestation/reforestation), i.e., the 50%-LCP and 100%-LCP scenarios. Therefore, while acknowledging the uncertainty, our analysis primarily focuses on net afforestation scenarios using the default approach.

As suggested by Cook-Patton et al. (2020)[70], the mitigation potential of restoring forest cover depends on two key factors: the potential area available for forest restoration (referred to as the "area of opportunity") and the rate at which these forests can sequester atmospheric carbon (measured in $tCO_2\ ha^{-1}\ yr^{-1}$). Cook-Patton et al. (2020) reported a 30-year A/R land removal intensity of 11.6 $tCO_2\ ha^{-1}\ yr^{-1}$, which was about 12% lower than the corresponding estimate of 13.1 $tCO_2\ ha^{-1}\ yr^{-1}$ in Griscom et al. (2017)[71]. Using the land carbon removal in $tCO_2\ ha^{-1}\ yr^{-1}$ allows us to capture changes in land area over time, which was overlooked when using the “mitigation density” approach from Roe et al. (2021) which only terminal land use change was used. In **Table S6**, we harmonize the land carbon removal intensity approach based on the data provided in sectoral studies. The “Mean LUC” is computed based on the trajectory of LUC in the study period. For example, in Austin et al. (2020)[72], A/R land expands faster in the period before 2035, resulting in a ratio between the terminal LUC and

mean LUC of about 1.5, which is smaller than 2 (linear interpolation). We observe a similar pattern in our study, as A/R takes time to accumulate carbon and is relatively cheaper in early periods. The derivation of land carbon removal intensity for our scenarios with net A/R (50%-LCP & 100%-LCP) is also provided in **Table S6**. On the other hand, energy-crop based BECCS tends to have a large ratio between the terminal LUC and Mean LUC as BECCS may expand over time at an increasing rate. Considering the trajectory of the land use, the land removal intensity improves the comparison across land-based mitigation measures, as the conventional approach may be biased towards A/R.

With a consistent definition, the land removal intensity of IAM models becomes more comparable, despite the large variability observed (**Figs. S28-S29**). However, caution is required when comparing the removal intensity between IAM projections and sectoral studies due to the potentially large differences in the definition and scope of the metric. For instance, it is crucial to distinguish between waste and residue-based BECCS, as failing to do so would result in a significantly higher land removal intensity for BECCS (**Fig. S30**).

S3.4. Agricultural prices vs. land carbon removal intensity

The distinct carbon removal processes of A/R and BECCS result in differences in temporal nonlinearity in land carbon removal intensity (**Section S3.3**). Here, we further investigated the relationship between agricultural prices and land removal intensity in our mitigation pathways, as both metrics are sensitive to the strength and scope of land mitigation policies and provide valuable insights. We first observed a significant positive correlation between agricultural prices and land removal intensity across our scenarios (see the first column in **Fig. S37**). Note that the agricultural prices are the production-weighted interannual global mean prices (so it considers the nonlinearity in the policy constraints), and the waste and residue-based BECCS is not included in the removal intensity calculation. However, this positive relationship is primarily driven by variations in LULUCF efficiencies, as indicated by the decomposition shown in the last two columns of **Fig. S37** (Method 1). Method 1 is preferred in this analysis as it focuses on scenarios with a strong emphasis on A/R, which provides land variations predominantly related to forest land.

The observation is further confirmed by regression models (**Table S8**) that (1) Model 4, which includes both explanatory variables, explains the largest variation in agricultural prices, (2) the coefficient of LULUCF (vs. Forest) removal intensity is statistically significant at a 1% level, and (3) the coefficient of energy crop based BECCS removal intensity is not statistically significant. These results align with our theoretical expectation that (1) A/R is more effective when implemented on low-carbon land, which often coincides with managed agricultural land such as cropland and managed pasture, leading to higher agricultural prices, and (2) the relationship is not evident in our scenarios for BECCS due to the stronger market-mediated responses and weaker dependency on the land carbon incentives.

S3.5. Limitations and future work

Our analysis has certain limitations that should be acknowledged. First, we did not account for potential future climate change impacts on agricultural and forestry production. These impacts, especially under high warming scenarios, could introduce additional feedback dynamically between mitigation efforts and agricultural production[73]. However, the climate change impacts on agricultural and forestry productivity are uncertain, and in low warming scenarios with limited overshooting, such impact should be relatively moderate[74,75]. Second, we did not include other CDR measures, such as direct air capture with carbon storage (DACCS), enhanced

weathering (EW), and biochar[17], or other agricultural practices and carbon management. In particular, the application of agricultural practices such as no-till and cover crops may increase the carbon storage in cropland. However, the mitigation potential of agricultural practices is likely small compared to A/R or BECCS[69,71]. Thus, further considering agricultural practices likely have little impact on the rank order of land carbon density (or land carbon removal intensity) across different land type and the land allocation under land carbon mitigation policies investigated in our study. The potential impacts of other CDR measures, given the high uncertainty in future deployment, under different land mitigation policies warrant further investigation and analysis. Moreover, we did not consider the unplanned reversal of carbon storage in both land (soil and vegetation) and geologic formations (e.g., BECCS). The durability and reversibility differences in land-based mitigation measures[76], like potential increased wildfire risks for A/R and the potential for transportation and storage leakage for BECCS, should be addressed in future research. Lastly, the interaction between land-based mitigation policies and other scenarios or policy dimensions, such as alternative socioeconomic pathways and non-CO_2 GHG mitigation policies, should be explored in future studies.

There are also some technical uncertainties in the IAM modeling in our study:

1) Land use modeling and land mitigation policy

As detailed in **Sections S2.2-S2.4**, our land allocation method implies that land competes grossly based on rental profits, and the land transition matrix is not produced. That is, land use is modeled while net land use change is derived based on the land allocation difference across periods. Note that transition-based land use modeling approaches have been developed and tested in previous studies. However, they relied heavily on assumptions or data that did not widely exist, e.g., the transition function in Ferreira Filho et al. (2015)[29], or did not consider economic responses, e.g., the Markov model in Li and Wu (2022)[30]. The uncertainty in land use modeling approaches[6] should be addressed in future studies.

In addition, our study does not include land protection policies mainly because in a competitive market, protecting land for carbon storage and our land carbon pricing policy approach could be theoretically and functionally equivalent[77]. However, in practice, the two could be different in implementation. For instance, land protection policies typically focus on the preservation and conservation of land resources for maintaining ecological integrity and providing ecosystem services with the primary goals of safeguarding biodiversity, natural habitats, and cultural heritage[78]. On the other hand, the land carbon pricing policy in this study focuses on carbon storage, one of the key ecosystem services lands can provide, as we aim to highlight the role of land in contributing to carbon mitigation. Nevertheless, valuing nature and ecosystem services is uncertain, and land policies are subject to significant institutional challenges (e.g., measurement, reporting, and verification)[67,68,79]. And the co-benefits or trade-offs in ecosystem services have not been explicitly considered in our framework and should be considered in future work[80,81].

2) Market-mediated responses and agricultural price impacts

It is crucial for IAM models to capture market-mediated responses[21,31,82,83] to represent factors such as price-induced resource reallocation, technological progress, and sectoral transitions, which have proven to be important in our analysis. Considering market-mediated responses is also essential for examining food security implications. While our agricultural price projections generally align with the AR6 pathways (**Main Fig. 6**), it is worth noting that less than 5% of the AR6 pathways reported agricultural prices. The limited reporting of agroeconomic variables in the AR6 Scenario Database hampers its usefulness. Given that agricultural prices are one of the most critical sustainability metrics, future IAM results submissions should prioritize reporting these prices.

A few recent studies have also demonstrated a wide range of agricultural price projections. For example, Fuhrman et al. (2020)[64] projected a threefold to sevenfold increase in agricultural prices (index for corn, wheat, & rice prices) by 2100 (relative to 2010) in 1.5°C pathways using GCAM, depending on the presence of DACCS and large-scale BECCS deployment. Fajardy et al. (2021)[4] indicated a roughly 75% increase in world average crop prices by the end of the century compared to 2015 under the default 1.5°C scenario with BECCS, driven primarily by price increases in the reference scenario (e.g., almost 60% in the BAU scenario).

The extent to which market-mediated responses are considered, as well as the strength of the response, are critical factors in determining the impacts on agriculture and food prices in mitigation pathways. In addition to what is discussed in our studies, another important aspect of market-mediated responses is the income (GDP) response. Our version of GCAM did not include an endogenous macroeconomic response that allows GDP to respond to mitigation policies in the mitigation pathways. A negative impact of mitigation on GDP could result in greater reductions in food consumption, thereby influencing agricultural prices to a lesser extent. Future studies should further examine the modeling structure and parameters that govern market-mediated responses in IAMs.

3) Cumulative carbon dioxide emissions targeting

In our study, we focus on end-of-century cumulative carbon dioxide emissions rather than directly targeting temperature or other climate variables. By adopting this approach, we avoid the uncertainties associated with translating emissions and other forcing agents into climate outcomes within our modeling framework. However, uncertainties still persist regarding the biophysical impacts (e.g., albedo and evapotranspiration) and other climate forcers[84–86].

Our mitigation scenarios exhibit small to moderate global carbon emission overshooting. The magnitude of overshooting is reduced when stronger land mitigation policies are implemented, particularly through the near-term deployment of A/R. For example, in the 2°C Main scenario, the 100%-LCP scenario experiences a peak emission of 1194 $GtCO_2$, which is only slightly higher (4% higher) than the end-of-century target of 1150 $GtCO_2$. However, it is worth noting that the transient climate response to cumulative CO_2 emissions (TCRE) is subject to greater uncertainty when net negative carbon emissions are considered[2].

4) Data and parameter uncertainty

Like any modeling study, the results presented in this study are subject to sensitivity regarding the data and parameters used. In our analysis, we have incorporated the most recent updates in the GCAM agriculture and land use module[87] in the version of the model employed in this study. However, it is important to acknowledge that other key data and parameters, as discussed in **Section S2**, may still carry uncertainties. And key examples include factor productivity for future purpose-grown energy crop production and land carbon emission density. It is worth noting that all the data and modeling utilized in this study are publicly available, ensuring transparency and reproducibility. While addressing data and parameter uncertainty is not the primary focus of the present study, it represents an avenue for exploration in future research.

Table S3 | Shadow price of carbon by sector in GCAM scenarios (2010$ per tCO2). This table includes data used in **Fig. 2A** in the main paper.

Scenario	Year	No-LCP				10%-LCP				50%-LCP				100%-LCP			
		FFI	BECCS	Land-Forest	Land-Non Forest	FFI	BECCS	Land-Forest	Land-Non Forest	FFI	BECCS	Land-Forest	Land-Non Forest	FFI	BECCS	Land-Forest	Land-Non Forest
2C Main	2025	43	43	0	0	41	41	4	4	37	37	19	19	36	36	36	36
2C Main	2050	173	173	0	0	162	162	16	16	149	149	75	75	145	145	145	145
2C Main	2100	759	342	0	0	712	355	71	71	655	415	327	327	636	478	636	636
A/R-Focused	2025					40	40	4	0	35	35	18	0	33	33	33	0
A/R-Focused	2050					160	160	16	0	140	140	70	0	131	131	131	0
A/R-Focused	2100					702	349	70	0	616	387	308	0	575	420	575	0
Low-Bioenergy	2025	65	61	0	0	55	53	6	6	48	48	24	24	45	45	45	45
Low-Bioenergy	2050	257	114	0	0	213	101	23	23	180	92	96	96	169	91	181	181
Low-Bioenergy	2100	1160	313	0	0	966	328	100	100	819	317	422	422	772	325	794	794
1.5C	2025	83	83	0	0	75	75	8	8	68	68	34	34	66	66	66	66
1.5C	2050	330	276	0	0	301	299	30	30	270	270	135	135	265	265	265	265
1.5C	2100	1447	402	0	0	1320	393	132	132	1186	417	593	593	1160	458	1160	1160

Table S4 | Distribution of carbon prices in AR6 pathways (2010$ per tCO_2). This table includes data used in **Fig. 2B** in the main paper. The variable name in IPCC AR6 Scenario Database is "Price|Carbon."

AR6 CB	Year	10th	25th	50th	75th	90th	Mean	n
(175,500]	2025	11	25	73	134	203	140	95
(175,500]	2050	116	187	445	691	2103	1184	95
(175,500]	2100	287	436	1208	1857	5222	4096	95
(500,825]	2025	9	17	46	79	132	67	205
(500,825]	2050	114	203	315	556	973	635	205
(500,825]	2100	272	367	866	1701	6056	2467	205
(825,1150]	2025	11	17	37	52	72	39	183
(825,1150]	2050	77	127	182	307	876	366	183
(825,1150]	2100	226	316	528	1062	2157	966	183
(1150,1475]	2025	8	16	26	37	51	27	82
(1150,1475]	2050	66	88	110	146	213	133	82
(1150,1475]	2100	200	268	447	955	1461	667	82
[175, 1475]	2025	10	17	38	71	122	64	565
[175, 1475]	2050	78	132	219	461	897	568	565
[175, 1475]	2100	229	356	699	1359	3137	1994	565

Table S5 | Connecting land use to land-based carbon removal. This table summarizes the data and metrics used for connecting land to carbon removal and the calculations of land carbon removal intensity. Values shown are for 2020 – 2100 mean values, if not otherwise stated. Note that the marginal land carbon density is computed as the ratio between land carbon stock change and land use change. In scenarios involving net afforestation, the forest's "marginal land carbon density" should be greater than the land removal intensity (LULUCF vs. forest) in absolute values, indicating additionality.

	Variable & Metric	2C Main				A/R-Focused			Low-Bioenergy				1.5C			
		No-LCP	10%-LCP	50%-LCP	100%-LCP	10%-LCP	50%-LCP	100%-LCP	No-LCP	10%-LCP	50%-LCP	100%-LCP	No-LCP	10%-LCP	50%-LCP	100%-LCP
Land-based CDR ($GtCO_2$ / yr)	Carbon Budget	14.2				14.2			14.2				6.2			
	BECCS & LULUCF	-3.8	-4.6	-5.8	-6.3	-4.7	-6.3	-7.2	-1.3	-2.9	-4.5	-5.2	-6.7	-7.4	-8.3	-8.7
	BECCS	-6.6	-6.1	-5.0	-4.4	-6.1	-5.1	-4.5	-2.9	-2.6	-2.4	-2.4	-9.3	-8.7	-7.5	-6.7
	BECCS | Purpose-grown	-4.4	-4.0	-3.1	-2.6	-4.0	-3.2	-2.8	-1.3	-1.1	-0.9	-0.8	-6.1	-5.5	-4.5	-3.9
	BECCS | Residue & MSW	-2.2	-2.1	-1.9	-1.8	-2.1	-1.8	-1.7	-1.5	-1.5	-1.6	-1.5	-3.3	-3.1	-2.9	-2.8
	LULUCF	2.8	1.5	-0.7	-1.9	1.4	-1.2	-2.7	1.6	-0.2	-2.1	-2.9	2.6	1.3	-0.9	-2.0
Primary bioenergy (EJ /yr)	Total	153	146	130	119	147	134	125	67	66	66	66	166	161	146	134
	Purpose-grown	97	90	76	65	92	80	71	29	27	23	22	105	100	86	75
	Purpose-grown | CCS	78	71	57	48	72	59	51	23	19	16	14	96	89	75	66
	Purpose-grown | NonCCS	19	20	19	17	20	21	20	7	7	8	7	10	10	10	10
	Residue	49	48	47	46	48	47	46	30	32	36	37	53	53	53	52
	Residue | Crop	39	39	38	37	38	37	36	21	23	27	28	43	44	43	43
	Residue | Forestry	10	10	9	9	10	10	10	9	9	9	9	10	10	9	9
	MSW	8	8	8	8	8	8	8	7	7	7	7	8	8	8	8
Biogenic carbon ($GtCO_2$ / yr)	Total	12.9	12.3	11.0	10.0	12.4	11.3	10.5	5.6	5.6	5.6	5.6	14.0	13.5	12.3	11.3
	Purpose-grown	8.2	7.6	6.4	5.5	7.7	6.7	6.0	2.5	2.2	2.0	1.8	8.9	8.4	7.2	6.3
	Purpose-grown | CCS	6.5	6.0	4.8	4.0	6.0	5.0	4.3	1.9	1.6	1.3	1.2	8.1	7.5	6.3	5.5
	Purpose-grown | NonCCS	1.6	1.6	1.6	1.4	1.7	1.7	1.7	0.6	0.6	0.6	0.6	0.8	0.9	0.9	0.8
	Residue & MSW	4.8	4.7	4.6	4.5	4.7	4.6	4.5	3.1	3.4	3.7	3.8	5.1	5.1	5.1	5.0
	Residue & MSW | CCS	3.2	3.1	2.8	2.7	3.0	2.7	2.6	2.1	2.2	2.2	2.2	4.2	4.1	4.0	3.8
	Residue & MSW | NonCCS	1.6	1.7	1.8	1.9	1.7	1.8	2.0	1.0	1.2	1.4	1.5	0.9	1.0	1.1	1.2
Land use change w.r.t. 2020 (Mha)	Cropland | Energy	476	434	354	302	444	383	344	147	134	117	110	519	479	398	344
	Cropland | Energy | CCS	381	340	267	223	347	284	247	114	96	79	73	471	429	349	300
	Cropland | Energy | NonCCS	94	94	87	79	97	99	97	33	37	39	37	48	50	49	44
	Cropland | NonEnegy	40	-6	-92	-142	12	-49	-89	131	57	-48	-106	36	-22	-124	-179
	Forest & Other natural land	-247	-130	83	198	-115	163	348	-123	25	209	299	-268	-128	112	232
	Forest	-134	-51	98	181	36	400	648	-38	64	193	256	-150	-51	118	207
	Forest | Managed	84	72	52	41	85	101	122	108	88	65	53	81	66	42	29
	Forest | Unmanaged	-218	-123	46	140	-50	299	527	-145	-24	128	204	-231	-117	77	178
	Other natural land	-114	-79	-15	16	-150	-237	-300	-85	-39	16	43	-118	-77	-6	25
Land carbon stock change ($GtCO_2$ / yr)	Forest	-3.3	-1.6	1.1	2.4	0.0	6.1	9.7	-1.3	1.0	2.9	3.7	-3.0	-1.3	1.3	2.5
	Other natural land	-1.3	-0.8	-0.1	0.3	-1.8	-2.9	-3.6	-0.9	-0.2	0.4	0.6	-1.2	-0.7	0.0	0.3
	Others	1.7	0.9	-0.3	-0.8	0.4	-2.0	-3.4	0.6	-0.5	-1.2	-1.4	1.6	0.7	-0.4	-0.8
(Marginal) Land carbon density (tCO_2 / ha /yr)	Forest	24.4	30.9	11.2	13.1	-1.3	15.2	15.0	33.8	15.0	15.2	14.3	20.0	25.1	11.0	12.2
	Other natural land	11.2	10.3	5.3	17.4	12.0	12.3	12.1	10.3	4.9	22.7	14.3	10.3	9.7	5.7	12.5
	Others	7.0	6.7	3.6	3.9	3.8	12.2	9.7	4.7	22	5.8	4.7	5.9	5.6	3.4	3.5
Land carbon removal intensity (tCO_2 / ha /yr)	BECCS & LULUCF	-11.2	-11.9	-12.7	-13.1	-9.8	-8.0	-7.3	-11.8	-14.6	-14.6	-14.3	-18.1	-17.2	-16.2	-15.8
	BECCS (Purpose-grown) & LULUCF	-4.7	-6.4	-8.5	-9.3	-5.4	-5.7	-5.5	2.3	-6.8	-9.5	-10.1	-9.2	-9.9	-10.5	-10.8
	BECCS	-14.0	-14.0	-14.2	-14.6	-13.7	-13.3	-13.0	-19.5	-19.8	-20.8	-21.4	-18.0	-18.1	-18.7	-19.5
	BECCS | Purpose-grown	-9.3	-9.1	-8.8	-8.7	-9.0	-8.5	-8.1	-9.1	-8.2	-7.5	-7.4	-11.7	-11.6	-11.4	-11.4
	BECCS | Purpose-grown | CCS Land	-11.6	-11.6	-11.7	-11.8	-11.5	-11.4	-11.3	-11.7	-11.4	-11.2	-11.1	-12.9	-12.9	-13.0	-13.0
	LULUCF vs. Forest	-21.0	-29.7	-7.3	-10.4	39.8	-3.0	-4.2	-41.9	-3.7	-10.8	-11.2	-17.6	-25.6	-7.5	-9.8
	LULUCF vs. Forest & Other natural	-11.4	-11.6	-8.7	-9.6	-12.3	-7.3	-7.8	-12.9	-9.6	-9.9	-9.6	-9.9	-10.2	-7.9	-8.8
Key metrics	Purpose-grown | Yield (GJ per ha)	204	208	213	215	206	208	207	199	199	198	199	203	208	215	218
	Residue & MSW share in biogenic carbon	37%	38%	42%	45%	38%	40%	43%	56%	60%	65%	67%	37%	38%	41%	44%
	CCS sector share	75%	73%	69%	67%	73%	68%	65%	72%	68%	63%	61%	88%	86%	84%	82%
	CCS sector share | Purpose-grown	80%	78%	75%	74%	78%	74%	72%	77%	72%	67%	66%	91%	89%	88%	87%
	CCS sector share | Residue & MSW	67%	65%	61%	59%	65%	60%	57%	68%	65%	61%	59%	83%	81%	78%	76%
	Capture rate in CCS sectors	68%	67%	66%	66%	67%	66%	65%	71%	70%	69%	68%	76%	74%	72%	72%
	Share of biogenic carbon captured	51%	49%	46%	44%	49%	45%	43%	51%	47%	43%	42%	66%	64%	61%	59%

Table S6 | Deriving land carbon removal intensity. The land carbon removal intensity derivations from recent sectoral studies, as well as the results from the afforestation scenarios (50%-LCP & 100%-LCP) in the present study, are provided. The land use change (LUC) represents the terminal value, i.e., the last period relative to 2020. Note that the source data from the original studies were used. In cases where the land use trajectory was not provided, linear interpolation was used to calculate the interannual mean LUC.

Study	Land removal intensity	Scenario information	Scenario name	Study period	LUC (Mha)	Mean LUC (Mha)	Cumulative land use (Mha·yr)	Mean CDR ($GtCO_2$ yr^{-1})	Cumulative CDR ($GtCO_2$)	Mitigation density (tCO_2 ha^{-1})	Land removal intensity (tCO_2 $ha^{-1}yr^{-1}$)
Roe et al. (2021)	A/R	Adapted based on Austin et al. (2020). The study assumed a 2050 carbon price (2017 USD) of $2000/$tCO_2$ for the technical potential and $100/$tCO_2$ for the cost-effective scenario.	Technical potential	2020 - 2050	1423	949	28458	6.2	185	130	6.5
			Cost-effective	2020 - 2050	271	181	5418	1.0	29	108	5.4
	A/R	Adapted based on Busch et al. (2019). The study assumed a 2050 carbon price (2014 USD) of $500/$tCO_2$ for the technical potential and $100/$tCO_2$ for the cost-effective scenario.	Technical potential	2020 - 2050	1237	619	18556	8.3	250	202	13.5
			Cost-effective	2020 - 2050	184	92	2766	1.1	33	181	12.0
	Energy crop-based BECCS	Adapted based on the biomass to electricity & CCS scenario in Hanssen et al. (2020). The technical potential scenario was from the source study, while the cost-effective was a new estimate at $100/$tCO_2eq$.	Technical potential	2020 - 2050	740	370	11095	2.4	72	97	6.5
			Cost-effective	2020 - 2050	161	81	2416	0.5	15	93	6.2
Busch et al. (2019)	A/R	Initial A/R carbon prices. Global marginal abatement cost curves developed for tropical A/R.	$20 USD	2020 - 2050	32	16	477	0.2	5.7	179	11.9
			$50 USD	2020 - 2050	84	42	1262	0.5	15.1	180	12.0
Austin et al. (2020)	A/R	Initial A/R carbon prices & Hotelling rate. The Global Timber Model (GTM) was used.	$35@1%	2020 - 2055	317	224	7852	0.9	33	103	4.2
			$35@3%	2020 - 2055	453	280	9799	1.1	39	87	4.0
			$50@1%	2020 - 2055	400	285	9992	1.3	44	111	4.4
			$50@3%	2020 - 2055	560	351	12301	1.5	54	96	4.4
			$75@1%	2020 - 2055	522	373	13055	1.8	63	121	4.8
			$75@3%	2020 - 2055	685	441	15418	2.1	75	109	4.8
			$100@1%	2020 - 2055	600	434	15179	2.3	80	133	5.3
			$100@3%	2020 - 2055	777	508	17771	2.6	91	117	5.1

Study	Land removal intensity	Scenario information	Scenario name	Study period	LUC (Mha)	Mean LUC (Mha)	Cumulative land use (Mha·yr)	Mean CDR ($GtCO_2$ yr^{-1})	Cumulative CDR ($GtCO_2$)	Mitigation density (tCO_2 ha^{-1})	Land removal intensity (tCO_2 $ha^{-1}yr^{-1}$)
This Study	LULUCF vs. A/R land	See **Methods**	2C Main & 50%-LCP	2020 - 2050	91	81	2442	0.8	25	281	10.4
			2C Main & 100%-LCP	2020 - 2050	177	143	4286	2.6	79	444	18.3
			A/R-Focused & 50%-LCP	2020 - 2050	337	236	7076	0.7	22	65	3.1
			A/R-Focused & 100%-LCP	2020 - 2050	568	395	11841	2.5	74	129	6.2
			Low-Bioenergy & 50%-LCP	2020 - 2050	169	117	3512	2.0	60	357	17.1
			Low-Bioenergy & 100%-LCP	2020 - 2050	242	176	5270	3.6	109	449	20.6
			1.5C & 50%-LCP	2020 - 2050	117	115	3451	1.7	50	430	14.6
			1.5C & 100%-LCP	2020 - 2050	209	190	5687	3.8	114	545	20.0
	Energy crop-based BECCS		2C Main & 50%-LCP	2020 - 2050	302	131	3925	0.3	9	31	2.4
			2C Main & 100%-LCP	2020 - 2050	255	113	3404	0.2	7	29	2.2
			A/R-Focused & 50%-LCP	2020 - 2050	312	135	4065	0.3	9	28	2.1
			A/R-Focused & 100%-LCP	2020 - 2050	271	121	3623	0.2	7	24	1.8
			Low-Bioenergy & 50%-LCP	2020 - 2050	125	78	2343	0.2	6	44	2.4
			Low-Bioenergy & 100%-LCP	2020 - 2050	116	72	2164	0.2	5	40	2.1
			1.5C & 50%-LCP	2020 - 2050	426	183	5497	1.2	35	82	6.4
			1.5C & 100%-LCP	2020 - 2050	377	151	4540	0.9	27	73	6.0
	LULUCF vs. A/R land		2C Main & 50%-LCP	2020 - 2100	101	98	7838	0.7	57	570	7.3
			2C Main & 100%-LCP	2020 - 2100	208	181	14514	1.9	152	728	10.4
			A/R-Focused & 50%-LCP	2020 - 2100	584	400	31981	1.2	95	163	3.0
			A/R-Focused & 100%-LCP	2020 - 2100	954	648	51863	2.7	217	227	4.2
			Low-Bioenergy & 50%-LCP	2020 - 2100	302	193	15420	2.1	167	551	10.8
			Low-Bioenergy & 100%-LCP	2020 - 2100	354	256	20519	2.9	230	650	11.2

Study	Land removal intensity	Scenario information	Scenario name	Study period	LUC (Mha)	Mean LUC (Mha)	Cumulative land use (Mha·yr)	Mean CDR ($GtCO_2$ yr^{-1})	Cumulative CDR ($GtCO_2$)	Mitigation density (tCO_2 ha^{-1})	Land removal intensity (tCO_2 $ha^{-1}yr^{-1}$)
			1.5C & 50%-LCP	2020 - 2100	123	118	9479	0.9	71	578	7.5
			1.5C & 100%-LCP	2020 - 2100	221	207	16575	2.0	162	735	9.8
	Energy crop-based BECCS		2C Main & 50%-LCP	2020 - 2100	670	354	28356	3.1	251	374	8.8
			2C Main & 100%-LCP	2020 - 2100	578	302	24190	2.6	210	363	8.7
			A/R-Focused & 50%-LCP	2020 - 2100	763	383	30679	3.2	260	341	8.5
			A/R-Focused & 100%-LCP	2020 - 2100	722	344	27542	2.8	223	308	8.1
			Low-Bioenergy & 50%-LCP	2020 - 2100	169	117	9367	0.9	70	416	7.5
			Low-Bioenergy & 100%-LCP	2020 - 2100	162	110	8792	0.8	65	398	7.4
			1.5C & 50%-LCP	2020 - 2100	608	398	31806	4.5	363	597	11.4
			1.5C & 100%-LCP	2020 - 2100	525	344	27553	3.9	313	596	11.4

Table S7 | Regression results for agricultural price models. This table presents results from Ordinary Least Squares (OLS) regressions estimating the relationship between crop price responses and the mitigation policy drivers. The dependent variable is the logarithm of the global staple crop price ratio between mitigation scenarios and the reference scenario. The table reports the estimated parameter (est.) and standard error (s.e.) for 8 models, each with a different set of explanatory variables, i.e., shadow prices of carbon (β^i) in different sectors (i). Note that $\beta limit^{BECCS}$ represents the carbon price penalty when a bioenergy limit is binding, and $\beta^{BECCS} = \beta^{FFI} - \beta limit^{BECCS}$. $S^{A/R-Focused}$ is a slope dummy variable; $S^{A/R-Focused} = 1$ if A/R-Focused scenarios are applied and $S^{A/R-Focused} = 0$ if all land carbon is included in land mitigation policies. In Model 8, β^{FFI} is labeled as $\beta^{EIP\ \&\ Land}$ since FFI carbon prices would represent the full system prices when sector-specific prices are not separated in the regression. Similarly, β^{FFI} is labeled as β^{EIP} when β^{BECCS} is not separated (i.e., in Models 2, 4, 6, and 7). The unit of est. and s.e. is percentage per $100 (2010 USD) increase in β^i, e.g., a 3.61% increase in global staple crop price is expected when the shadow price of carbon of BECCS increases by $100, holding other variables constant. The number of observations is 240 (15 scenarios x 16 study periods). All coefficients are statistically significant at the 0.001 level. This table supplements the analysis presented in **Fig. 6c** in the main paper.

Explanatory variable	Model 1		Model 2		Model 3		Model 4		Model 5		Model 6		Model 7		Model 8	
	est.	s.e.	est.	s.e.	est.	s.e.	est.	s.e.	est.	s.e.	est.	s.e.	est.	s.e.	est.	s.e.
$\beta^{EIP\ \&\ Land}$															2.51	(0.14)
β^{EIP}			2.87	(0.13)			2.87	(0.13)			0.84	(0.08)	0.84	(0.09)		
β^{FFI}	-0.74	(0.11)			-0.74	(0.11)			-0.66	(0.1)						
$\beta^{Land:\ Forest}$	1.36	(0.27)	1.36	(0.27)					1.51	(0.26)	3.35	(0.36)	4.90	(0.17)		
$\beta^{Land:\ NonForest}$	3.17	(0.26)	3.17	(0.26)					3.10	(0.25)	1.76	(0.37)				
β^{BECCS}	3.61	(0.21)			3.61	(0.21)			2.69	(0.27)						
$\beta limit^{BECCS}$			-3.61	(0.21)			-3.61	(0.21)								
β^{Land}					4.53	(0.12)	4.53	(0.12)								
$\beta^{Land} \cdot S^{A/R-Focused}$					-3.17	(0.26)	-3.17	(0.26)								
Intercept									2.08	0.41						
Adjusted R-squared	0.962		0.962		0.962		0.962		0.919		0.915		0.907		0.581	

Table S8 | Relationship between agricultural prices and land removal intensity in mitigation pathways. This table presents results from Ordinary Least Squares (OLS) regressions estimating the relationship between crop price responses and land removal intensity. The dependent variable is the logarithm of the global interannual mean staple crop prices (e.g., in 2010 US dollars). The table reports the estimated parameter (est.) and standard error (s.e.) for 4 models, each with a different set of explanatory variables, i.e., the logarithm of end-of-century land remove intensity by sector ($LRE_{T=2100}$). The data are shown in **Fig. S37**. The number of observations is 9 (scenarios with meaningful afforestation & removal intensity metrics). Significance levels are denoted as follows: *** ($p < 0.001$), ** ($p < 0.01$), * ($p < 0.05$), . ($p < 0.1$), and no symbol ($p \geq 0.1$).

Explanatory variable	Model1		Model2		Model3		Model4	
	est.	s.e.	est.	s.e.	est.	s.e.	est.	s.e.
Aggregate (energy crop-based BECCS & LULUCF) removal intensity ($\log(LRE_{T=2100}^{Energy\ crop-based\ BECCS\ \&\ LULUCF})$)	0.24 **	(0.06)						
Energy crop-based BECCS removal intensity (BECCS vs. energy) ($\log(LRE_{T=2100}^{Energy\ crop-based\ BECCS})$)			0.19	(0.16)			0.15	(0.08)
LULUCF (vs. Forest) removal intensity ($\log(LRE_{T=2100}^{LULUCF})$)					0.12 **	(0.03)	0.12 **	(0.02)
Intercept	5.22 ***	(0.12)	5.33 ***	(0.35)	5.49 ***	(0.06)	5.18 ***	(0.17)
Adjusted R-squared	0.665		0.038		0.692		0.776	

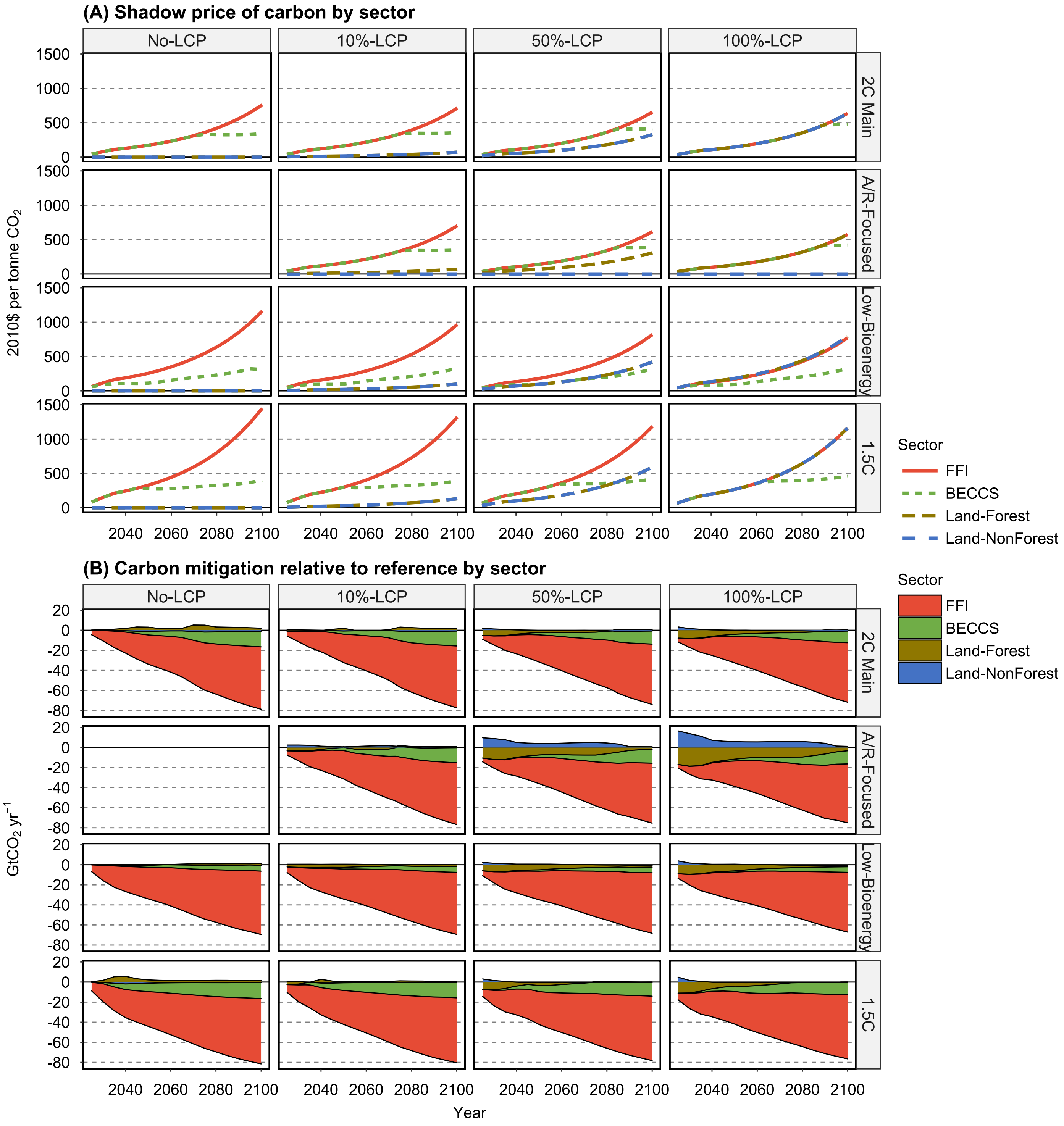

Fig. S13 | Shadow price of carbon (Panel A) and carbon mitigation relative to the reference scenario (Panel B) by sector in 2025 to 2100. Global carbon pricing is implemented in 2025. Note that the No-LCP scenario under A/R-Focused is identical to the No-LCP scenario under 2 °C Main. Data source: GCAM simulation results.

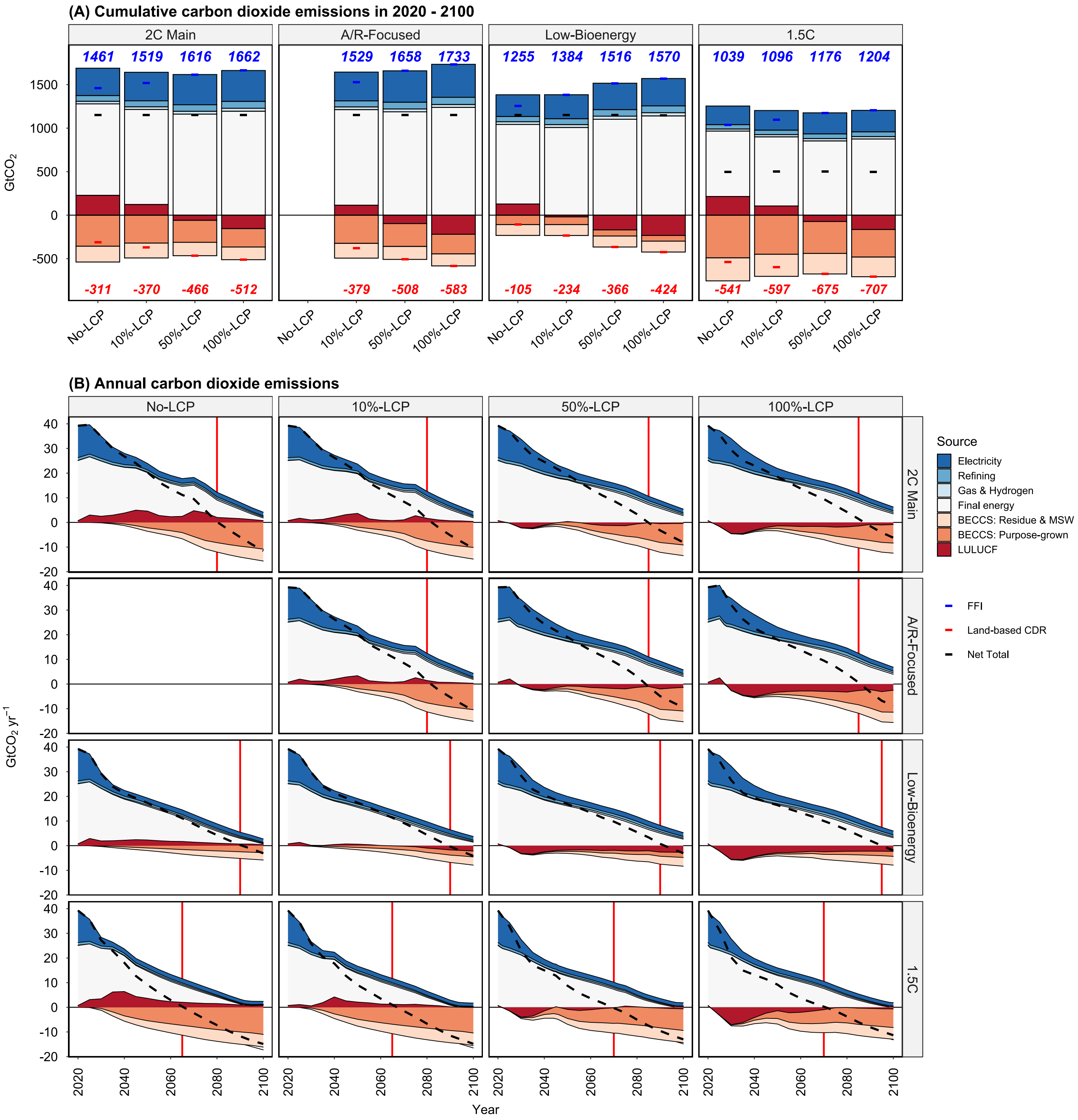

Fig. S14 | Decomposition of global 2020 – 2100 cumulative (Panel A) and annual (Panel B) carbon dioxide emissions. Carbon dioxide emissions/removals in panels (**A**) and (**B**) show contributions by Fossil Fuels and Industry (FFI), BECCS (by feedstock sources: residue & MSW or purpose-grown energy crop), and LULUCF. The final energy sectors include industry, buildings, and transportation. The peak carbon emission year and corresponding cumulative emissions are highlighted (red vertical lines) in panel (**B**). In panel **(A)**, the total FFI emissions ($GtCO_2$) and land-based CDR removals ($GtCO_2$) are annotated in blue and red numbers, respectively. This figure supplements **Figs. 3A, 3B, 4A, and 4B** in the main paper.

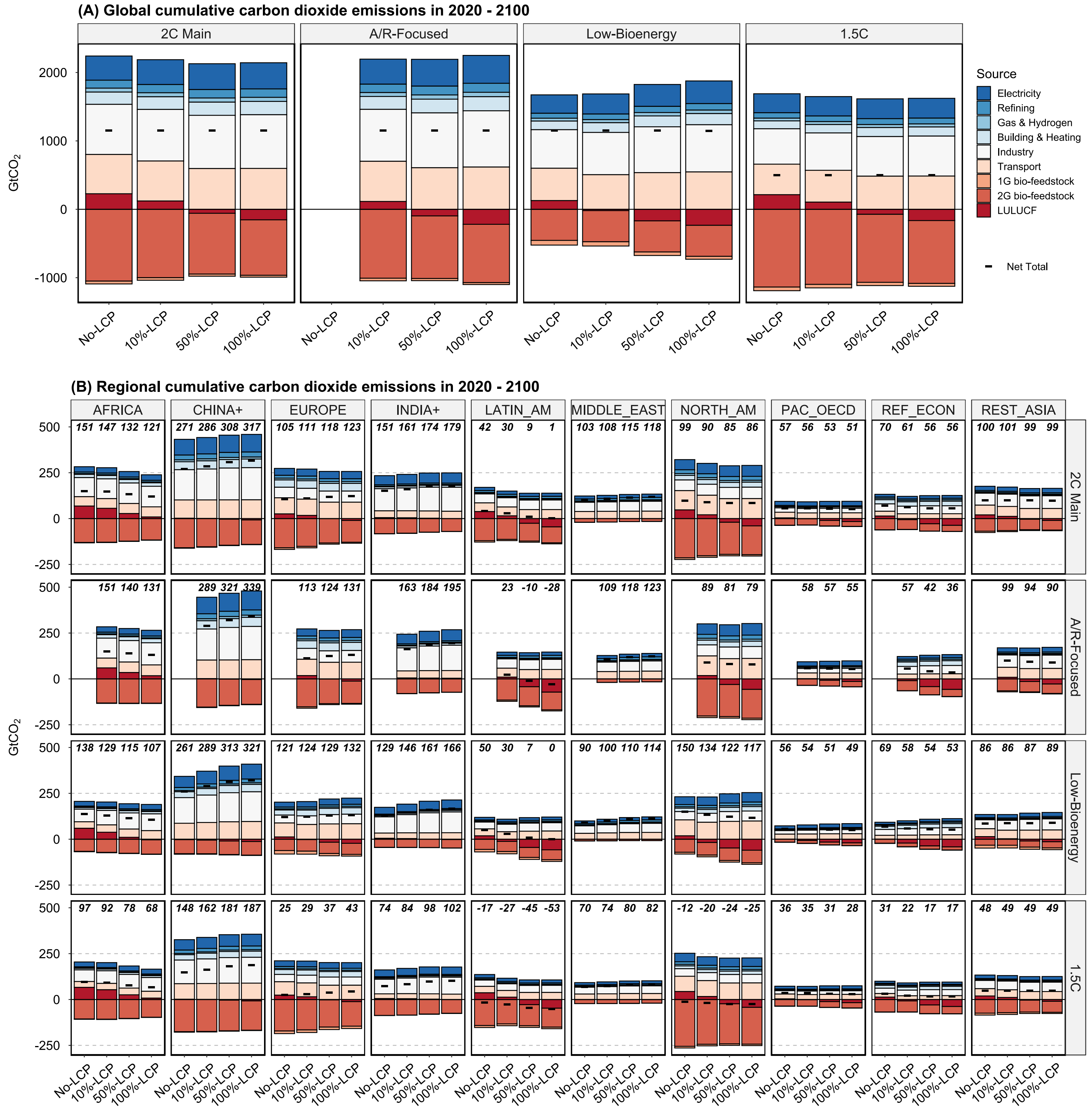

Fig. S15 | Decomposition of global (Panel A) and regional (Panel B) cumulative carbon dioxide emissions. Note that the decomposition partitions biogenic carbon in first-generation (1G) and second-generation (2G) bio-feedstock, so the positive emissions include uncaptured emissions from the combustion of both fossil fuels and bioenergy. In panel **(B)**, the regional net total carbon emissions ($GtCO_2$) are shown in black numbers for reference.

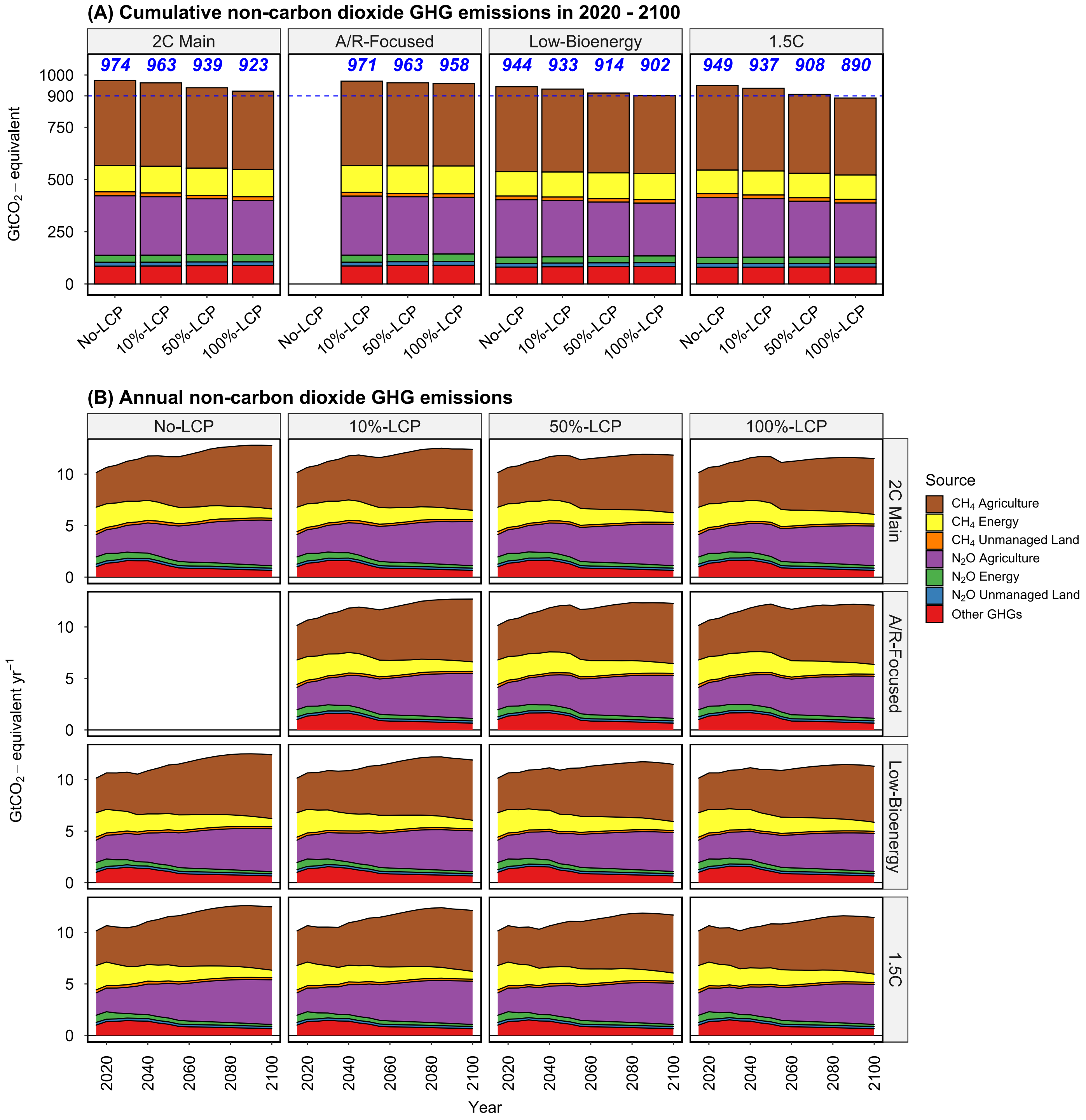

Fig. S16 | Projections of global 2020 – 2100 cumulative (A) and annual (B) non-carbon dioxide GHG emissions. Other GHGs are F-gases. The number (in blue) on the top of a bar plot in panel **(A)** indicates the total cumulative emissions. Note that unmanaged land emissions were from natural forest and grassland fire.

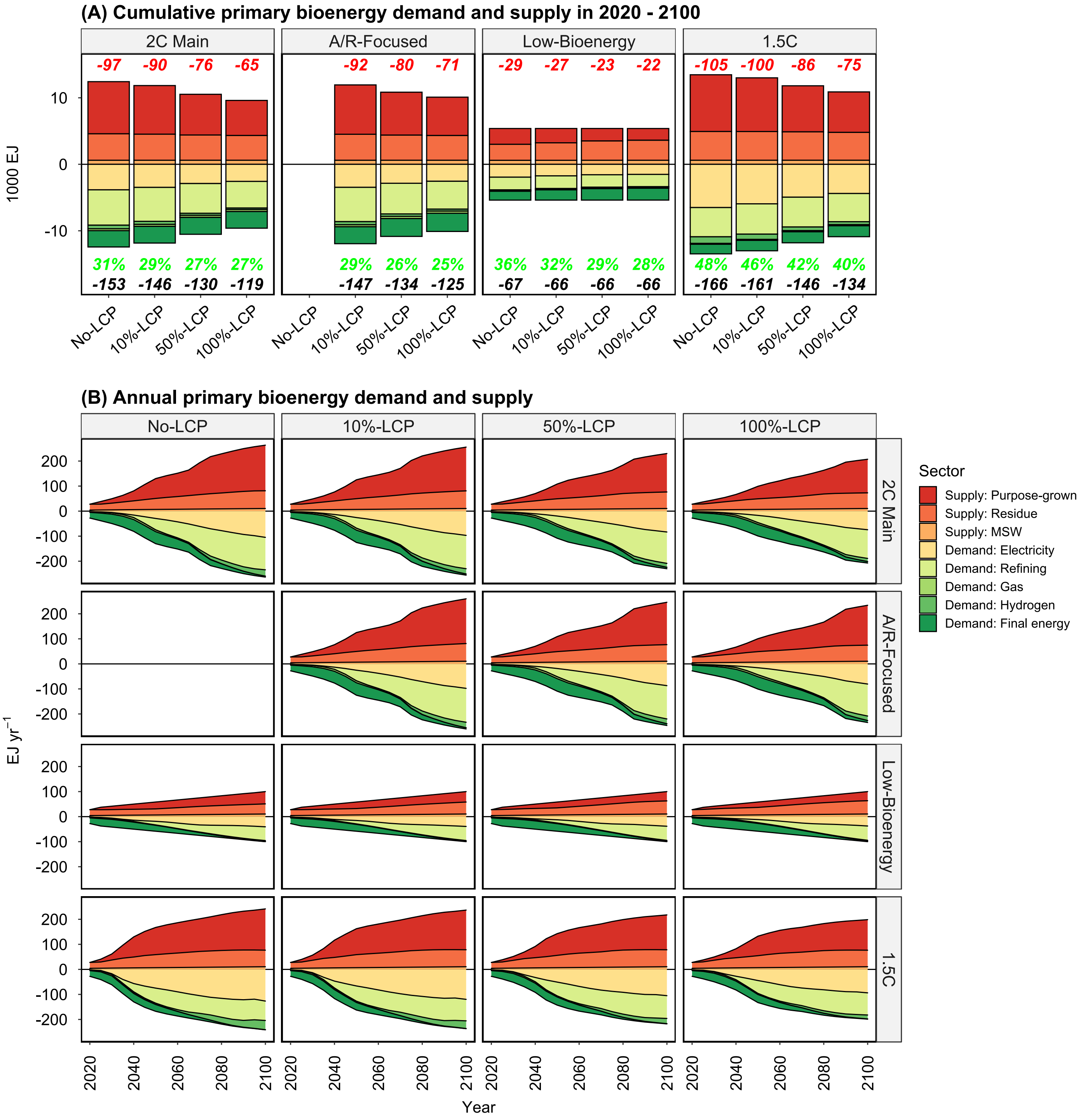

Fig. S17 | Projections of global 2020 – 2100 cumulative (A) and annual (B) primary bioenergy supply and demand. The positive values indicate supply by feedstock sources. The negative values indicate demand by sectors. In panel **(A)**, the total supply/demand (EJ yr^{-1}), the purpose-grown bioenergy supply (EJ yr^{-1}), and the share of biomass demand for electricity are shown in black, red, and green numbers, respectively.

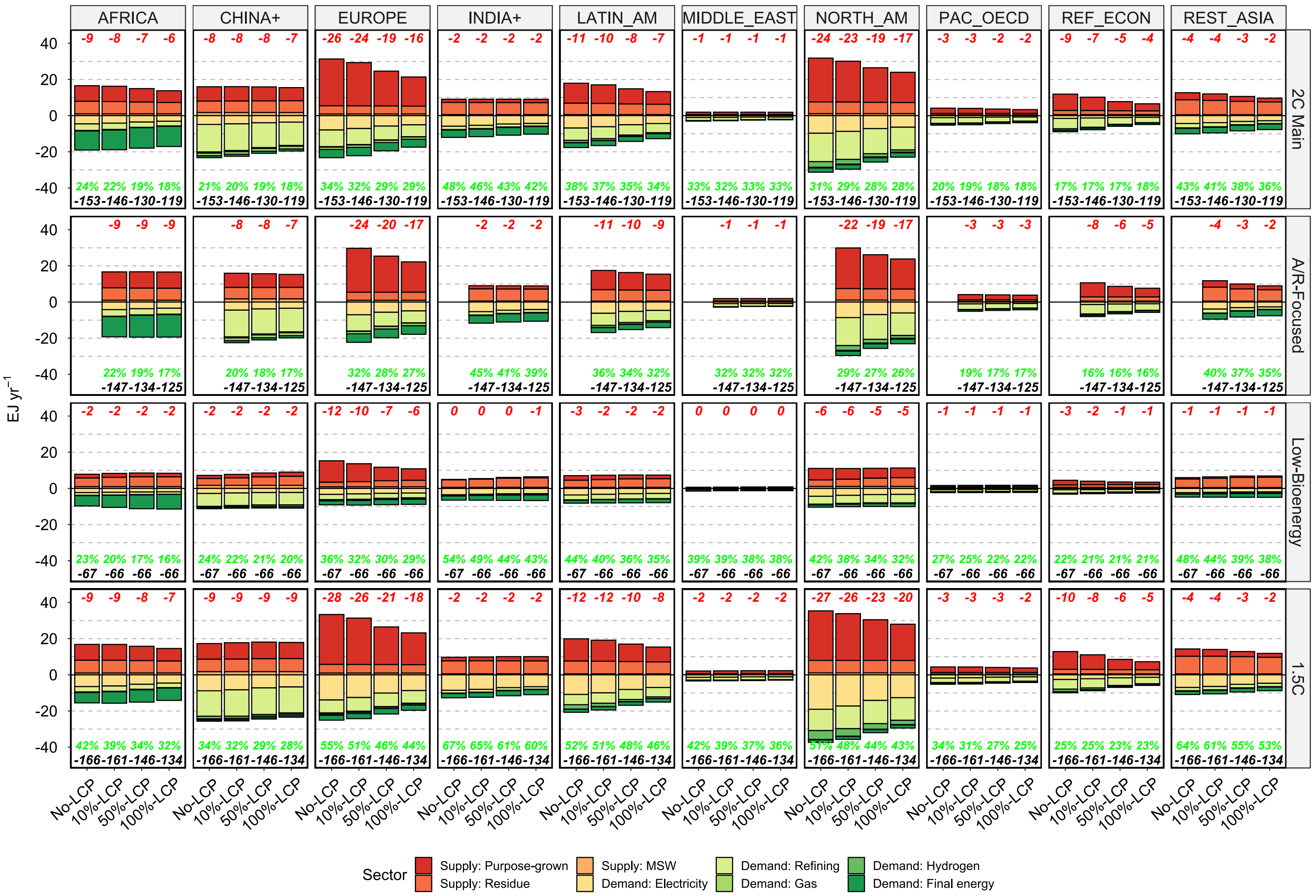

Fig. S18 | Projections of 2020 – 2100 regional (IPCC R10) cumulative primary bioenergy supply and demand. This figure presents a regional breakdown of **Fig. S17A**. Refer to the caption of **Fig. S17A** for a detailed description.

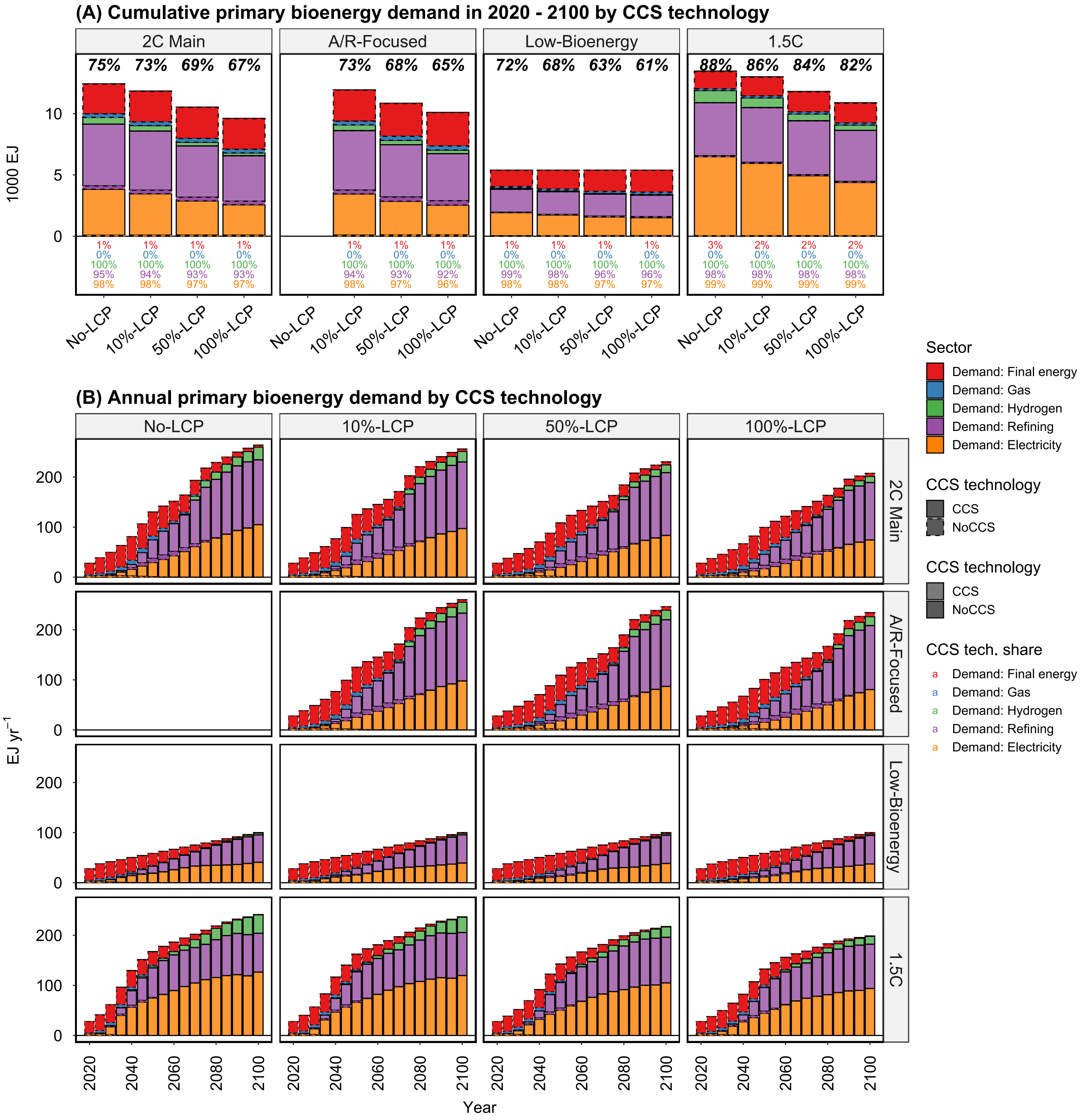

Fig. S19 | Projections of global 2020 – 2100 cumulative (A) and annual (B) primary bioenergy by demand sectors and CCS technology deployment. This figure breaks down the primary bioenergy demand shown in **Fig. S17** by the deployment of CCS technology (e.g., NoCCS indicates the energy is consumed in a sector/technology not combined with CCS). In panel **(A)**, the CCS technology deployment rate for bioenergy (i.e., the share of primary bioenergy use that is combined with CCS) is shown at the bottom by sector and the aggregate (weighted mean) values are shown in black at the top. Note that both line type (bar border) and transparency (filled color) are used to distinguish CCS technologies. Bars with dotted lines and darker filled colors indicate demand sectors/technologies with no CCS technologies. The final energy sectors include industry, buildings, and transportation. See additional details in **Section S2.8**.

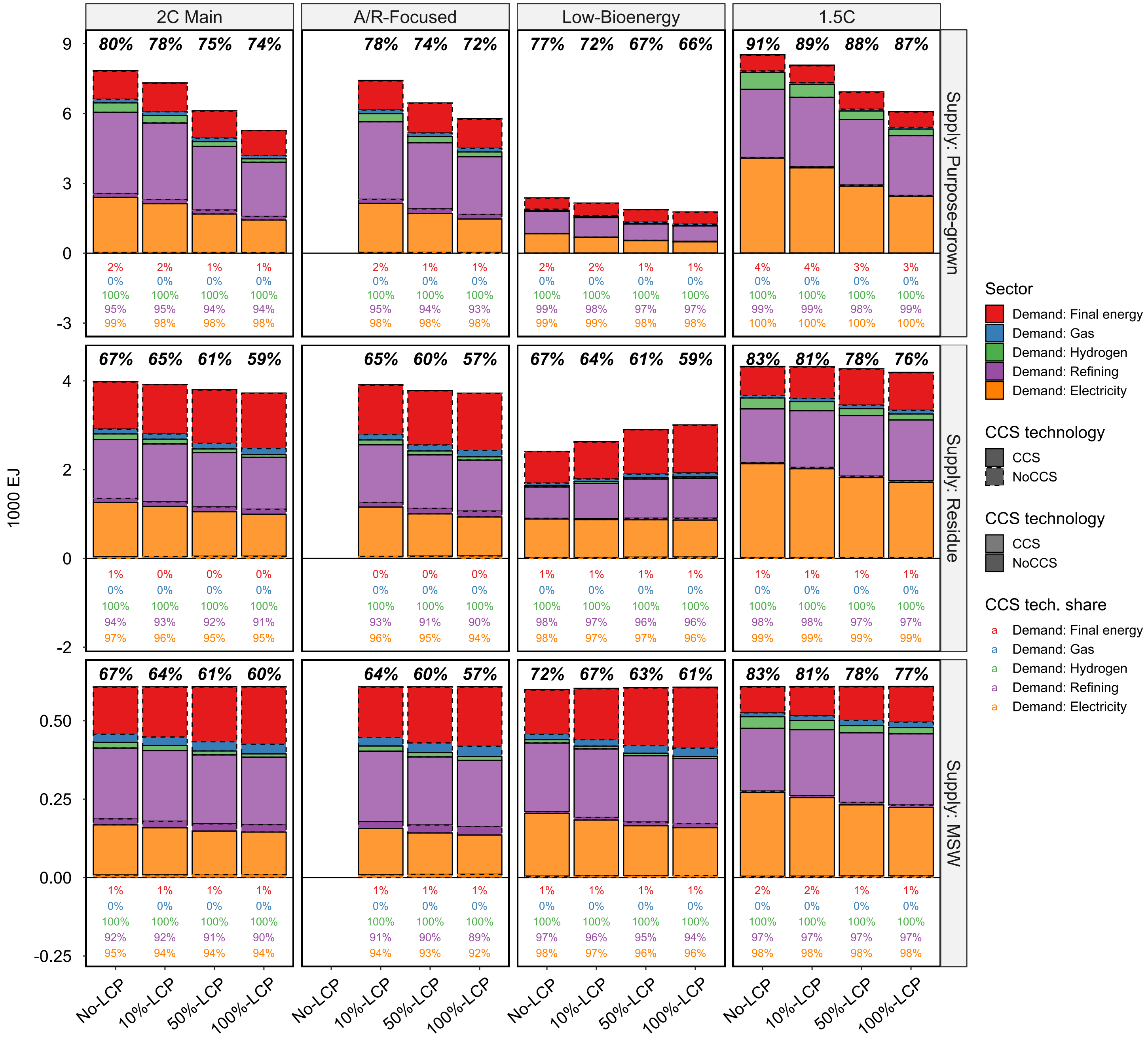

Fig. S20 | Projections of global 2020 – 2100 cumulative primary bioenergy by demand sectors, CCS technology deployment, and supply sectors. This figure provides a decomposition of Panel A of Fig. S19 by supply sectors. See more detailed captions in Fig. S19.

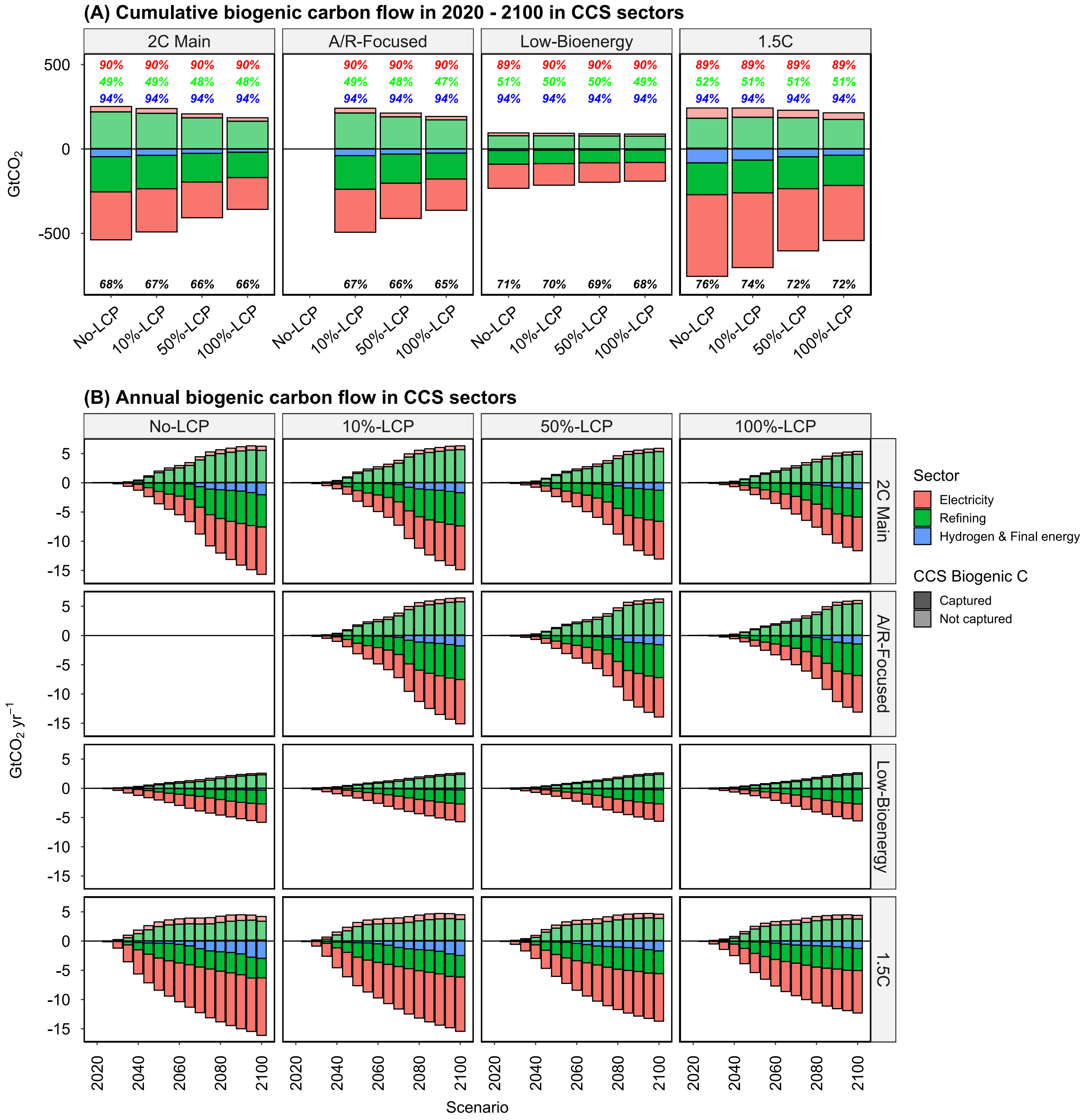

Fig. S21 | Projections of global 2020 – 2100 cumulative (A) and annual (B) primary biogenic carbon flow in CCS sectors by capture. The negative (positive) values indicate the captured (released) carbon when CCS technology is deployed. In panel **(A)**, the CCS efficiency (i.e., the share of captured over total carbon inflow) by sector is shown by color, and the total CCS efficiency is shown in black.

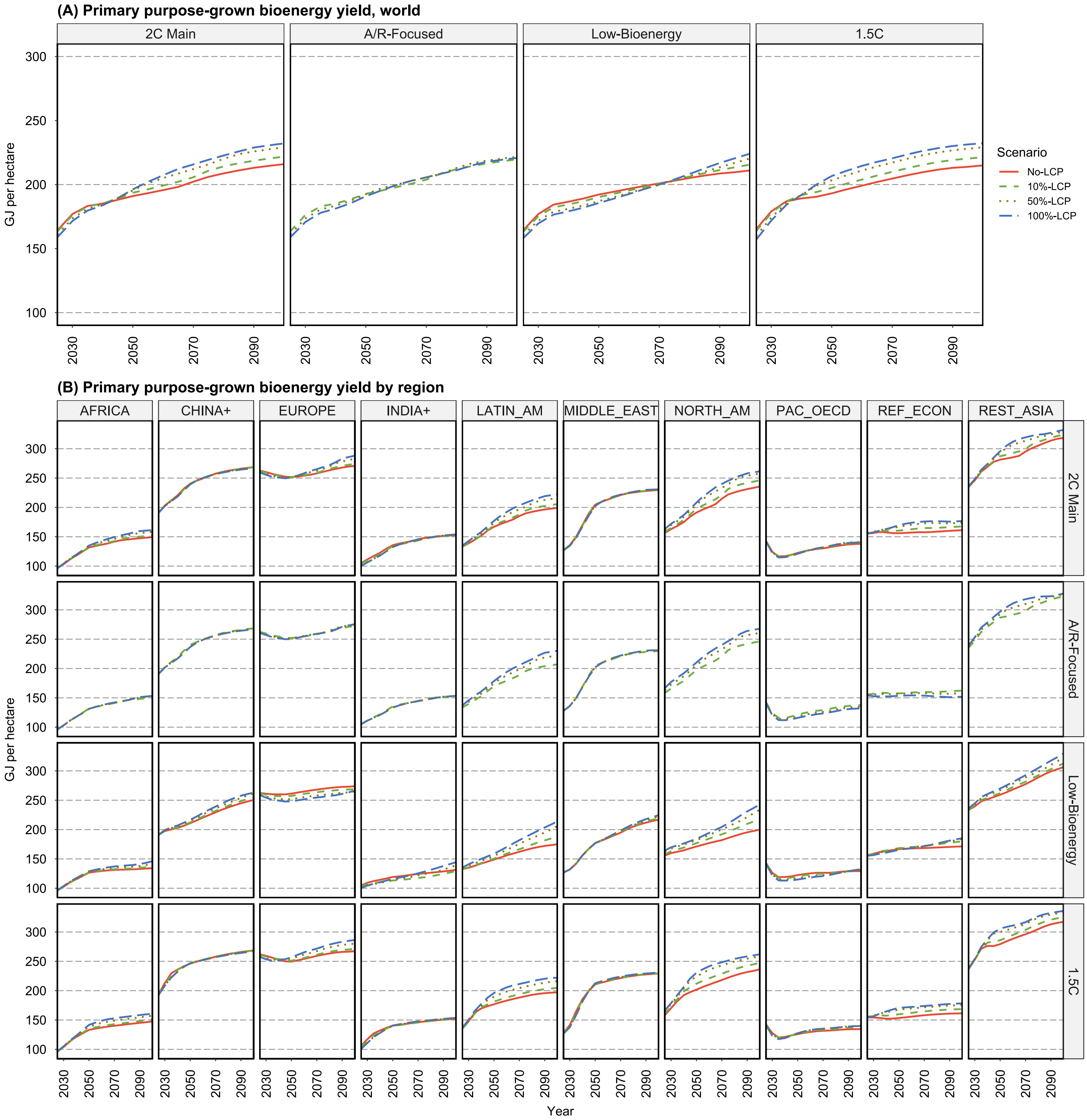

Fig. S22 | Projections of purpose-grown bioenergy crop yield at the world (Panel A) and regional (Panel B) levels. Note that GCAM assumes an energy content of 17.5 GJ per metric ton in primary biomass and a biogenic carbon content of 23 MtC per EJ.

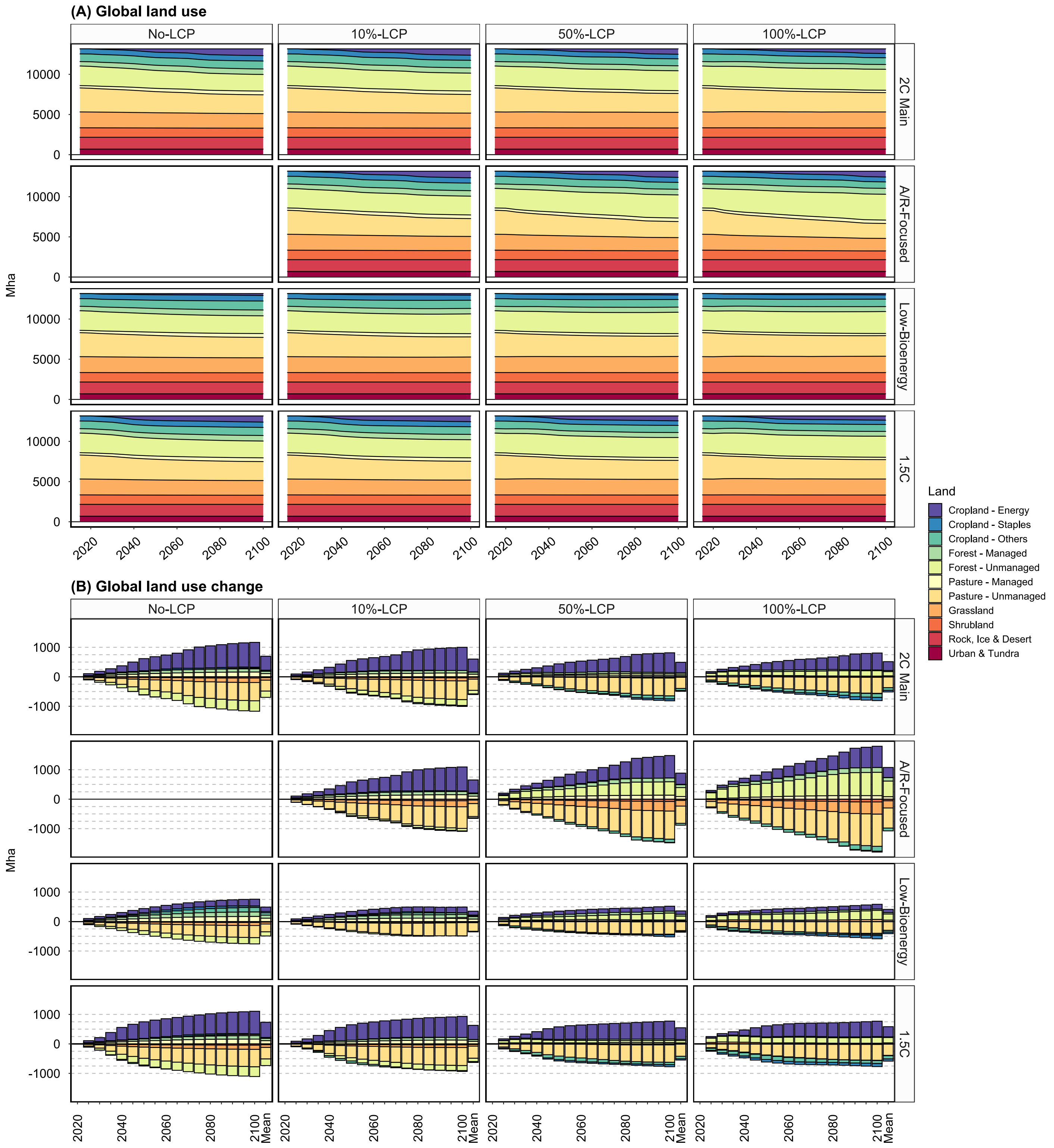

Fig. S23 | Global land use and land use change. Stacked areas or bars show projected land allocation (Panel A) and land use change relative to 2020 (Panel B) by land type. The annual mean value is added in Panel B (the last column in each panel).

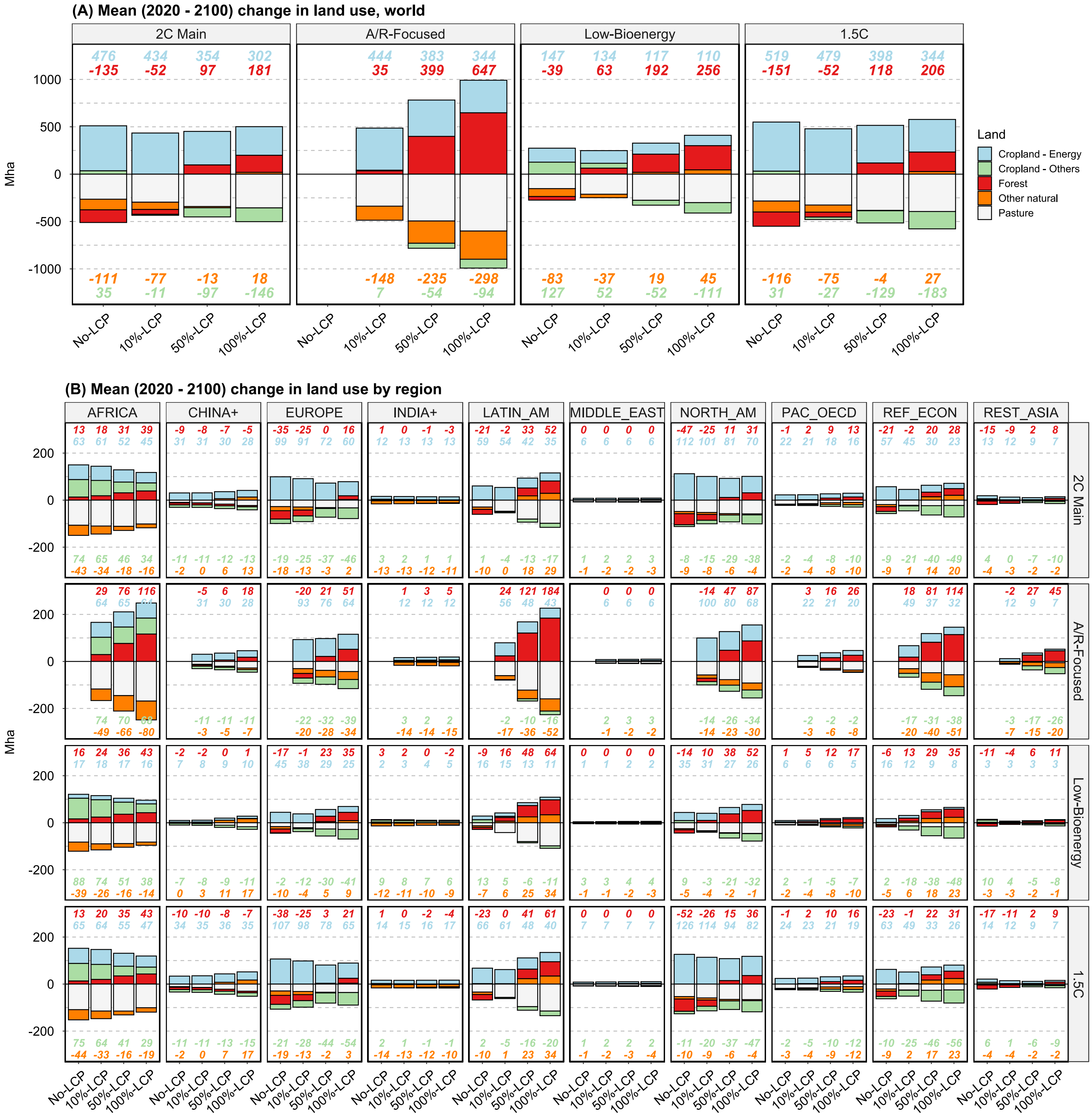

Fig. S24 | Global and regional mean (2020 – 2100) changes in land use. Stacked bars show projected annual mean changes in land use at the world (Panel A) and regional (Panel B) levels, with values shown by color (for cropland, forest, and other natural land).

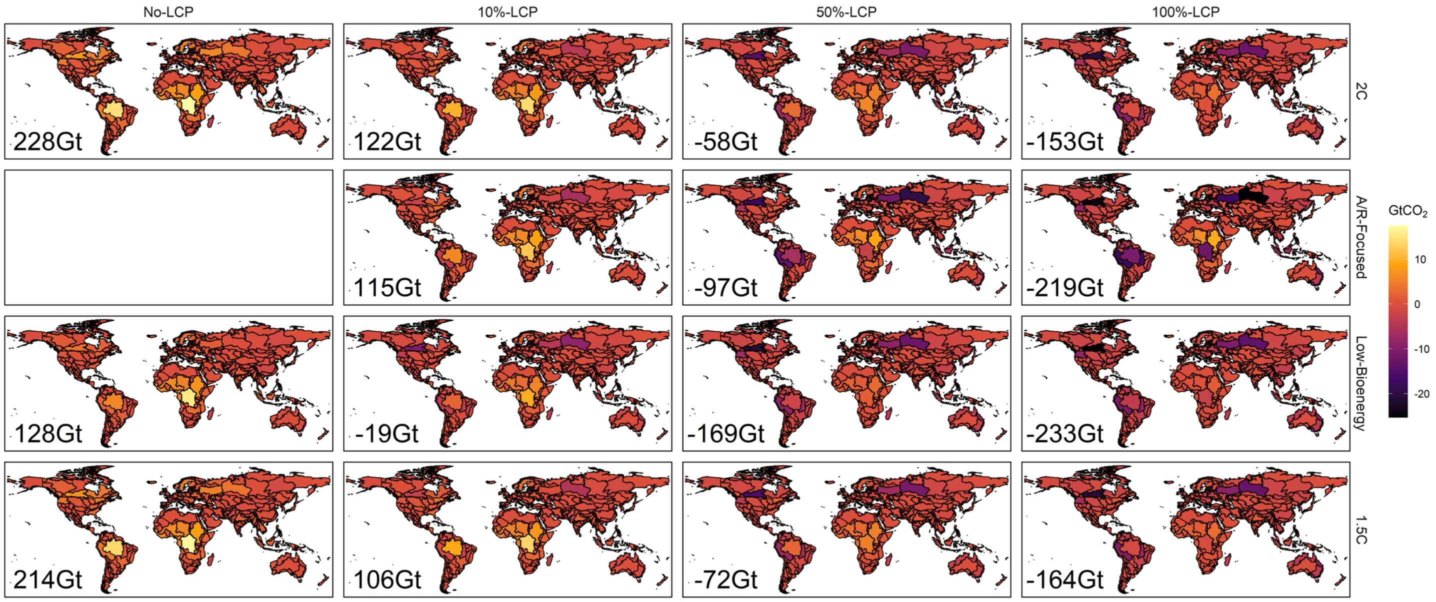

Fig. S25 | Net LULUCF CO_2 emissions. Global maps of the net total land use change CO_2 emissions by GCAM land modeling regions across the studied scenarios: 4 land carbon pricing strengths (columns) x 4 sets of policy scenarios (rows). Negative values indicate net sequestration. The global net total LULUCF CO_2 emission value is labeled in each map (lower-left corner). Note that 2C and A/R-Focused have the same results with no land carbon pricing (No-LCP), so the duplicate is not shown again. Data source: GCAM simulation results.

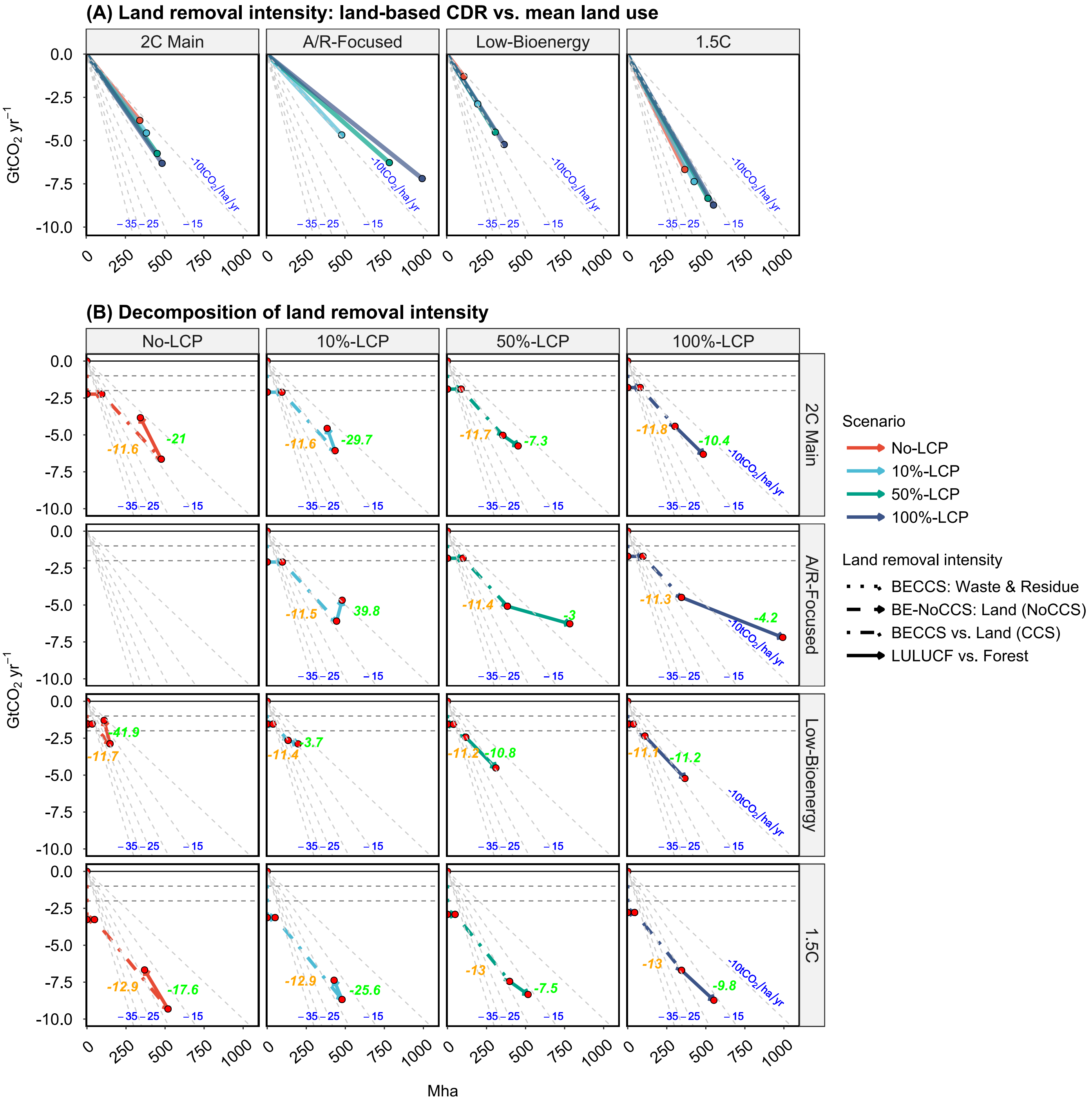

Fig. S26 | Land carbon removal intensity. Points in panel (**A**) present the relationship between the 2020 – 2100 mean land-based CDR and the corresponding land implication so that the slope of the lines implies 2020 – 2100 mean land carbon removal intensity. The slope of background lines (grey), for reference, is labeled (in blue with a unit of tCO_2 per hectare per year). Panel (**B**) decomposes panel (**A**) by land-based CDR sources and the corresponding land use, including waste & residue-based BECCS (vertical lines; no land attribution), energy cropland for bioenergy not used in sectors with CCS technologies (horizontal lines; no CCS attribution), energy crop-based BECCS vs. energy cropland for bioenergy used in sectors with CCS technologies (slopes annotated in orange), and LULUCF vs. forest (slopes annotated in green).

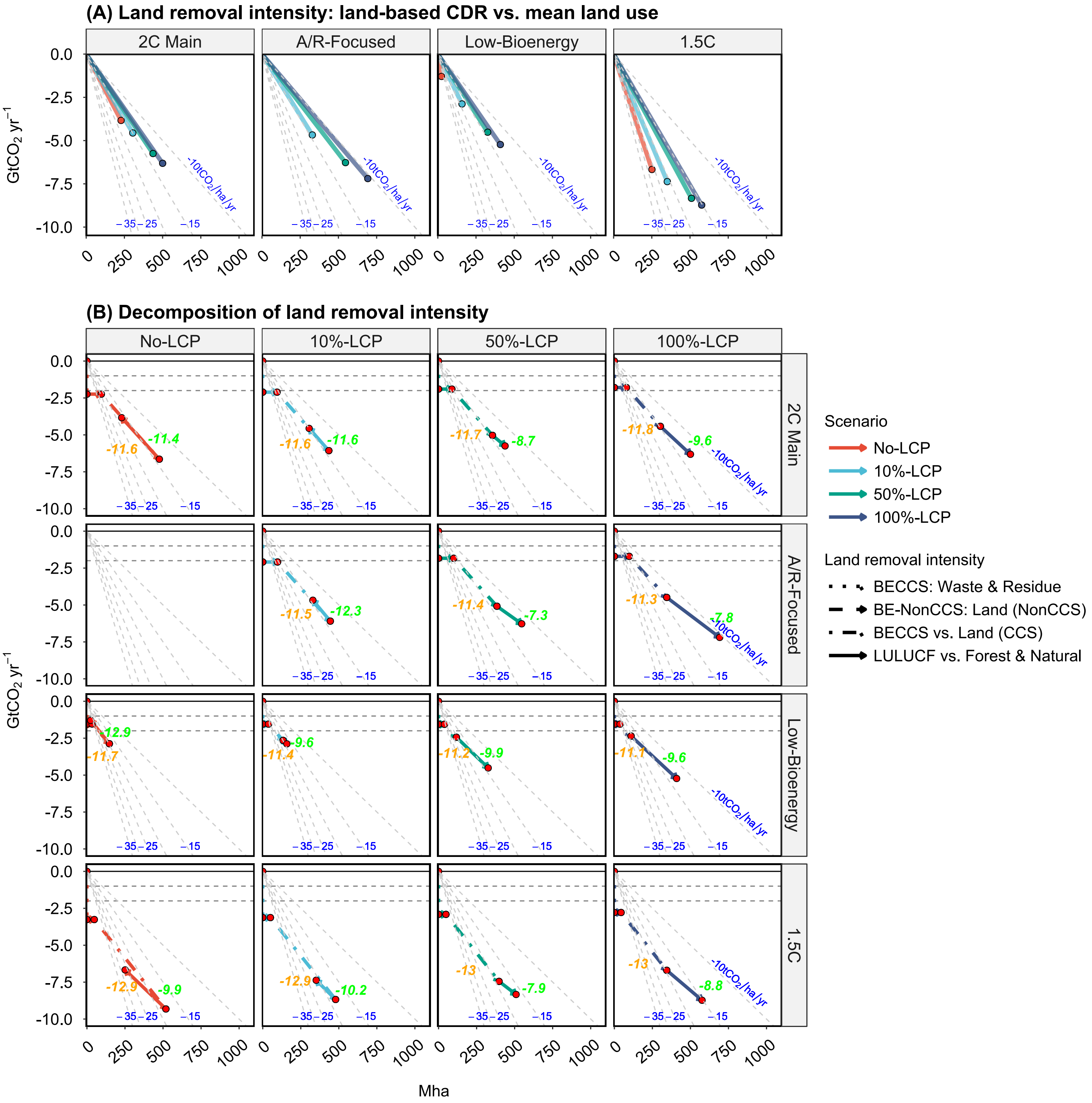

Fig. S27 | Land carbon removal intensity (alternative method for LULUCF removal intensity). Points in panel (**A**) present the relationship between the 2020 – 2100 mean land-based CDR and the corresponding land (with other natural land included) implication so that the slope of the lines implies 2020 – 2100 mean land carbon removal intensity. Panel (**B**) decomposes panel (**A**) by land-based CDR sources and the corresponding land use, and both forest and other natural land are attributed to LULUCF. See captions in **Fig. S26** for additional details.

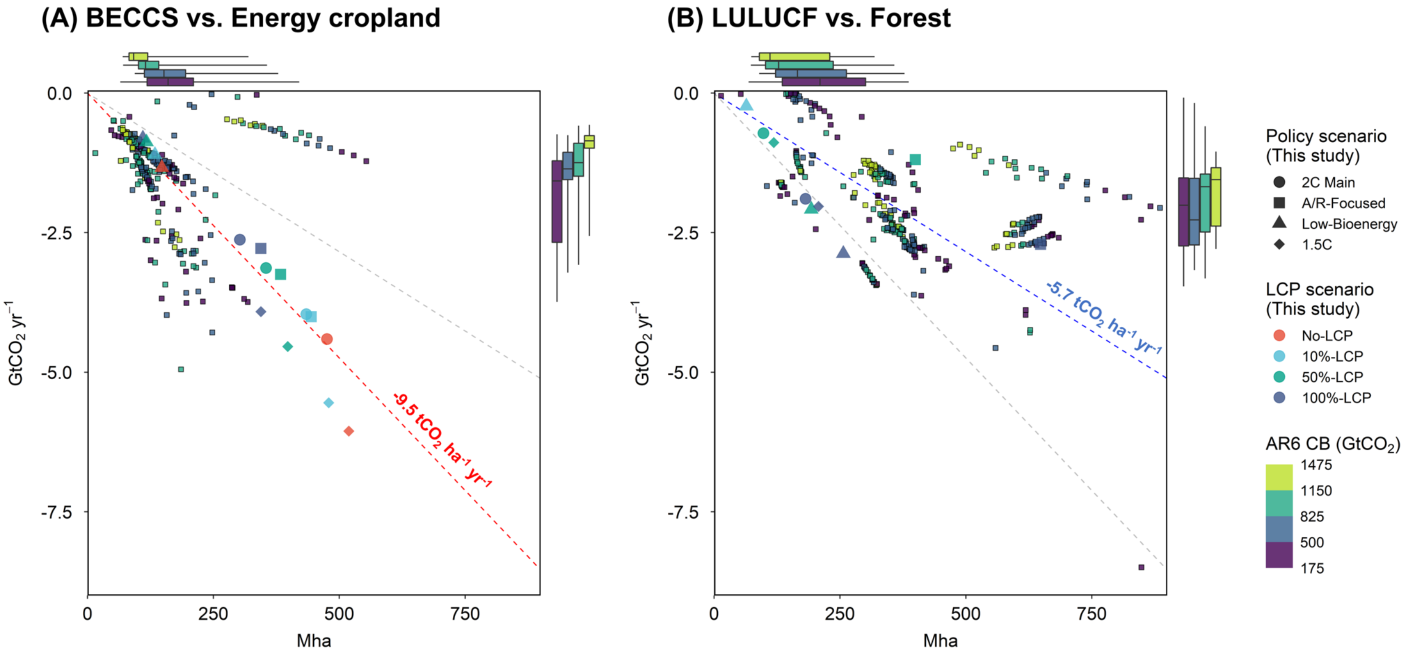

Fig. S28 | Relationship between 2020 – 2100 mean land-based carbon dioxide removal (CDR) and its corresponding land implication. **Panels (A)** and **(B)** depict energy crop-based BECCS versus purpose-grown energy cropland and LULUCF versus forest land, respectively, and the ratio (slope of the lines) represents the 2020-2100 mean land carb on removal intensity. Each dot represents a projection from an IPCC AR6 pathway (square) by carbon budget (CB) subrange (filled color) or from mitigation pathways generated in the present study using GCAM by policy scenario (point shape) and land-system carbon pricing (LCP) scenario (color). The boxplots on the sides show the distribution across AR6 pathways, including the median (horizontal line within the boxes), interquartile range (boxes), and 5 – 95 percentile range (whiskers), by study period and carbon budget (CB) subrange. The slope of the dotted line (annodated) indicates the median land removal intensity in AR6 pathways. This figure supplements **Fig. 5** in the main paper. Data sources: GCAM simulation results and AR6 Scenario Database.

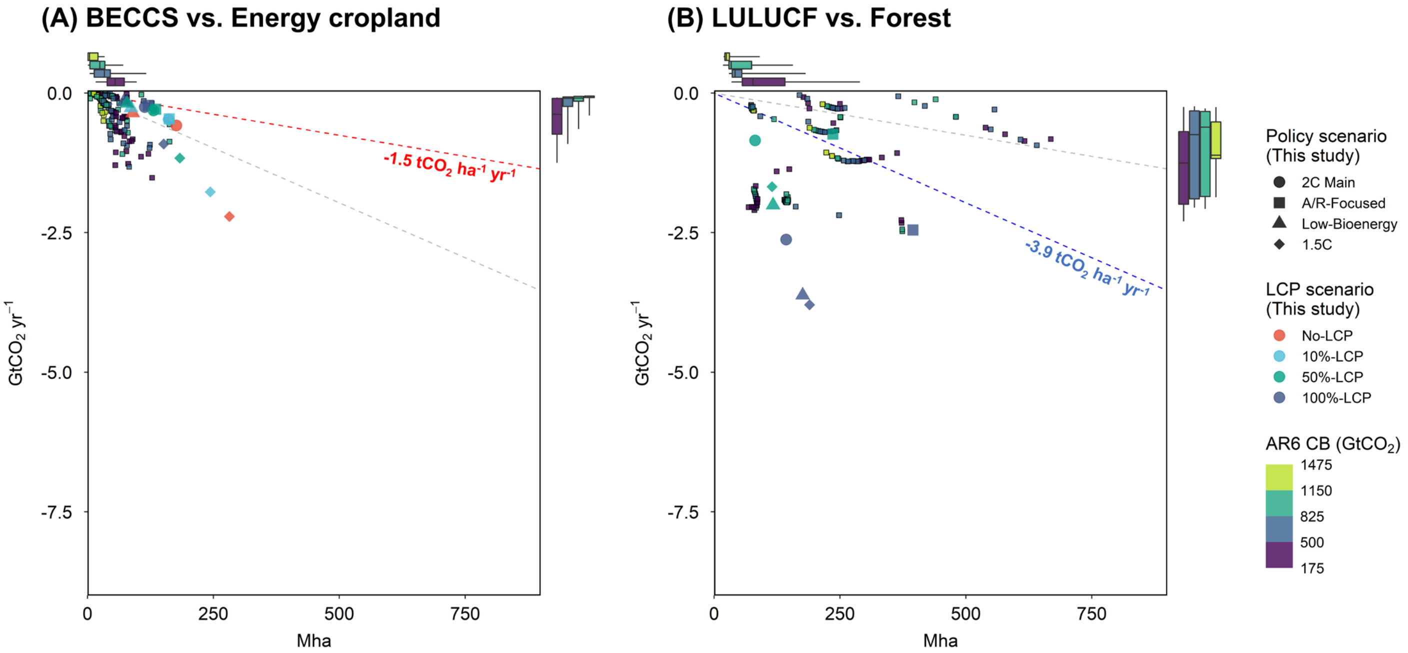

Fig. S29 | Relationship between 2020 – 2050 mean land-based carbon dioxide removal (CDR) and its corresponding land implication. Panels (A) and **(B)** depict energy crop-based BECCS versus purpose-grown energy cropland and LULUCF versus forest land, respectively, and the ratio (slope of the lines) represents the 2020-2050 mean land carb on removal intensity. Each dot represents a projection from an IPCC AR6 pathway (square) by carbon budget (CB) subrange (filled color) or from mitigation pathways generated in the present study using GCAM by policy scenario (point shape) and land-system carbon pricing (LCP) scenario (color). The boxplots on the sides show the distribution across AR6 pathways, including the median (horizontal line within the boxes), interquartile range (boxes), and 5 – 95 percentile range (whiskers), by study period and carbon budget (CB) subrange. The slope of the dotted line (annodated) indicates the median land removal intensity in AR6 pathways. Data sources: GCAM simulation results and AR6 Scenario Database.

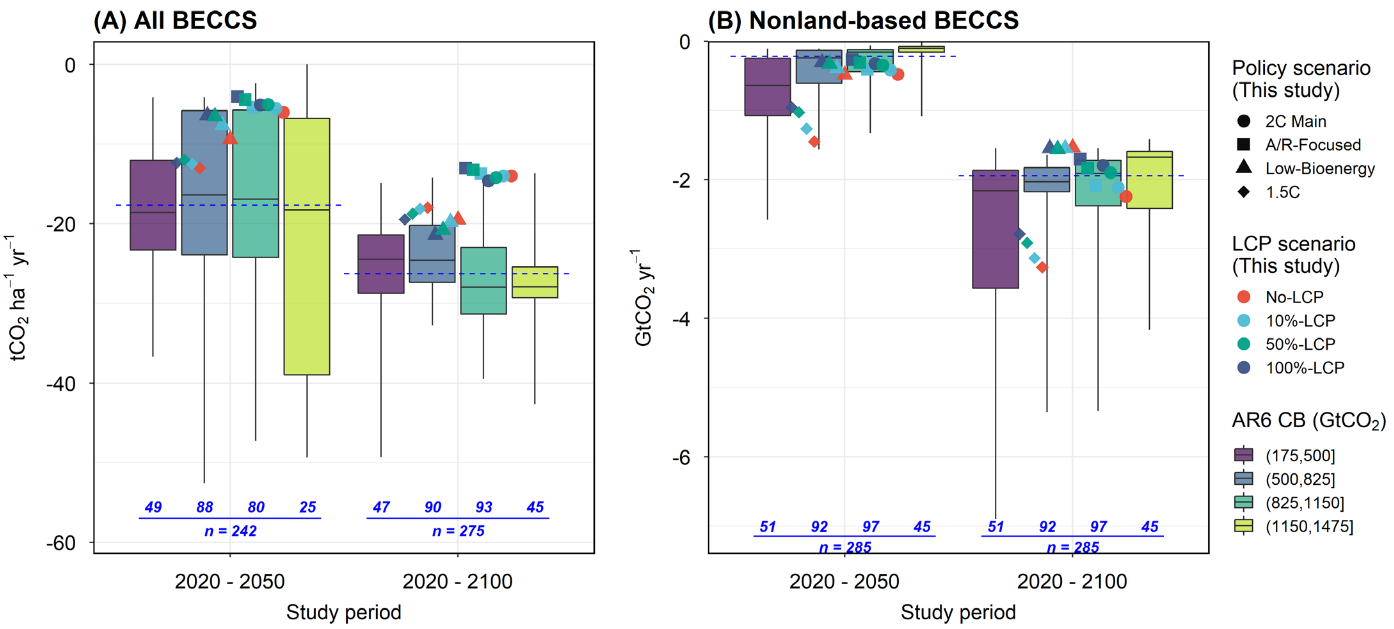

Fig. S30 | BECCS land carbon removal intensity decomposition. Panel (A) shows land removal intensity for BECCS (both land-based and nonland-based) versus purpose-grown energy cropland. See **Fig. 5** caption in the main paper for the detailed description. The negative values represent net removal in the study period. To enhance clarity in the presentation, the data used in **Panel (A)** has been truncated to include only AR6 pathways with a gross BECCS land removal intensity of less than 60 $tCO_2 ha^{-1} yr^{-1}$. **Panel (B)** shows the waste & residue-based BECCS deployment. After disaggregating waste & residue-based BECCS, the land removal intensity for energy crop-based BECCS is shown in **Fig. 5A** in the main paper. Data sources: GCAM simulation results and AR6 Scenario Database.

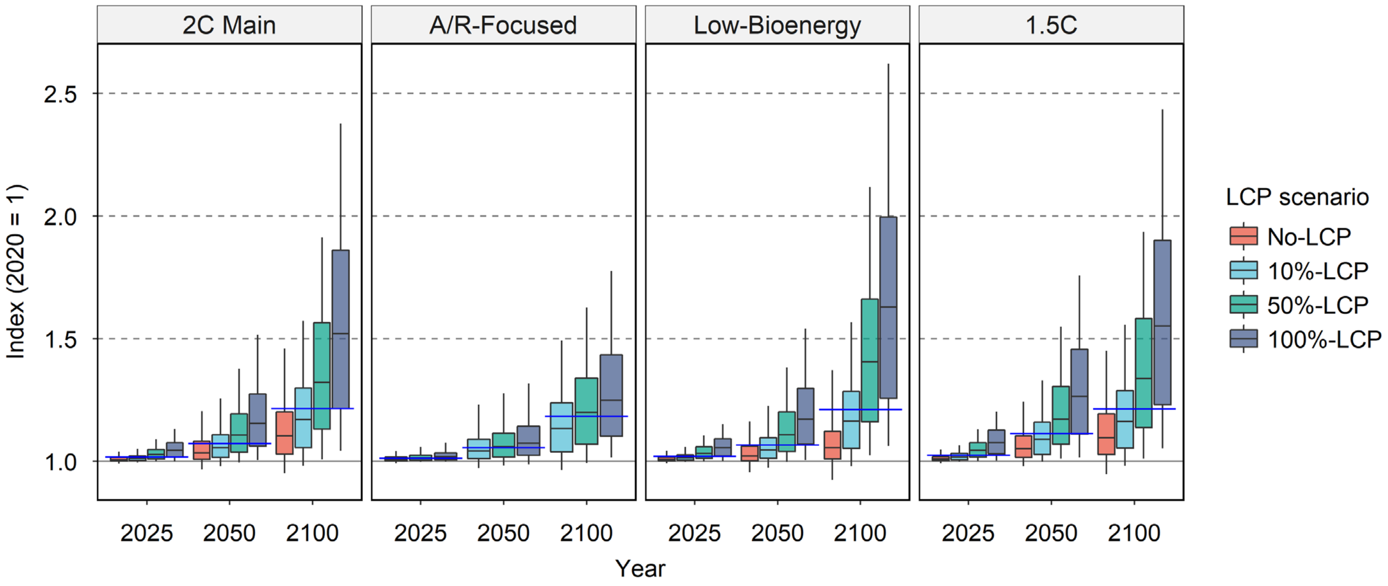

Fig. S31 | Agricultural price index distributions across GCAM sector-region combinations. The boxplots show the median (horizontal line within the boxes), interquartile range (boxes), and 5 – 95 percentile range (whiskers) of agricultural prices across combinations of GCAM region (n = 32; **Table S1**) and agricultural markets (n = 24; **Table S2**, not including OtherMeat&Fish), by year and scenarios. The blue line represents the median value in all LCP scenarios. This figure supplements **Fig. 6** in the main paper.

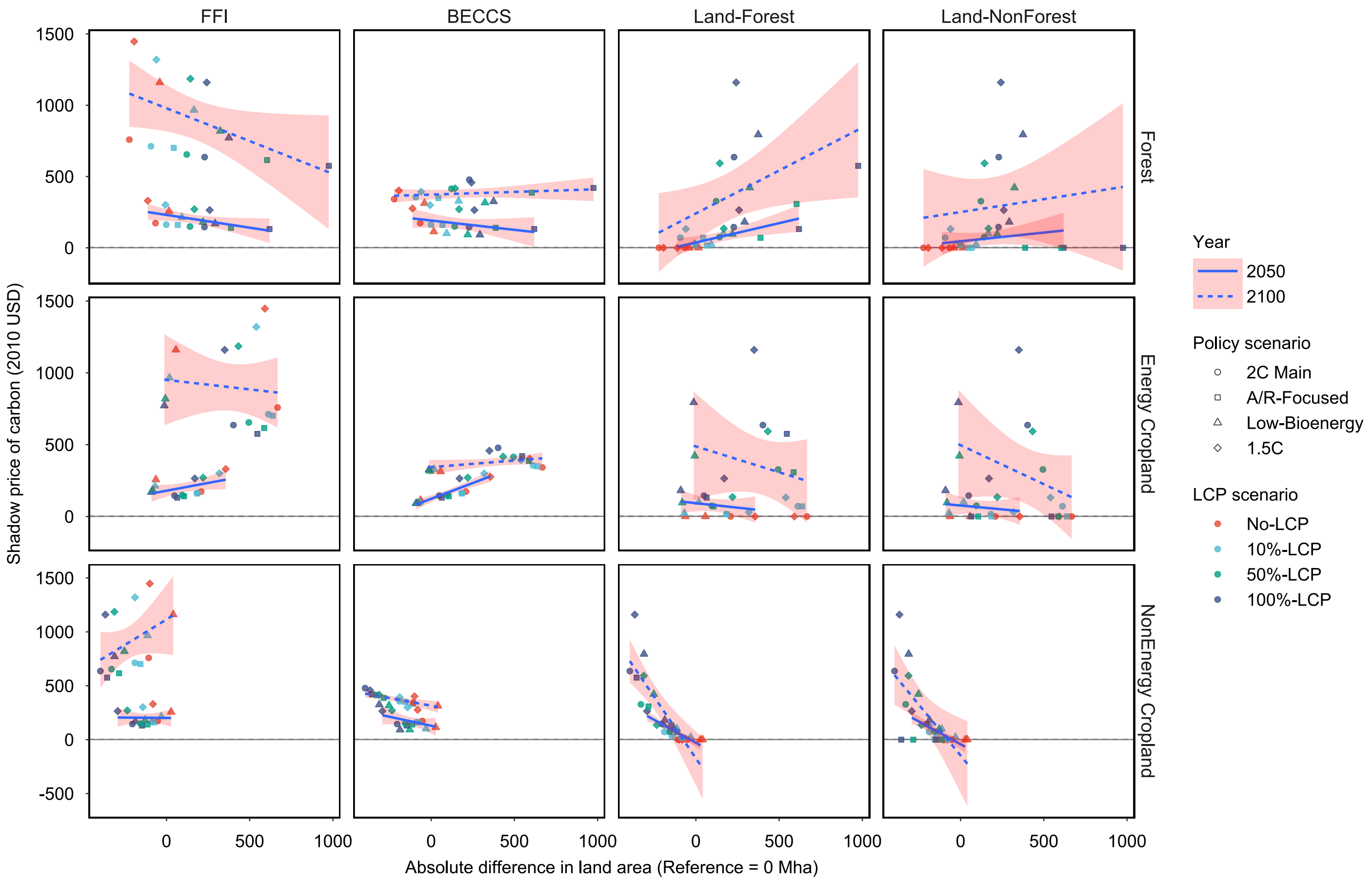

Fig. S32 | Relationship between shadow price of carbon and land use impacts (reference = 0 Mha). Panels show combinations of carbon markets (by column, i.e., FFI, BECCS, forest land, and nonforest land) and land sectors (by row, i.e., forest, energy cropland, and non-energy cropland). Points show the relationship by LCP scenarios (colors) and policy scenarios (shapes) for 2050 and 2100. Linear trend lines (blue lines) and 95% CI (grey ribbons) are added by year for each subpanel.

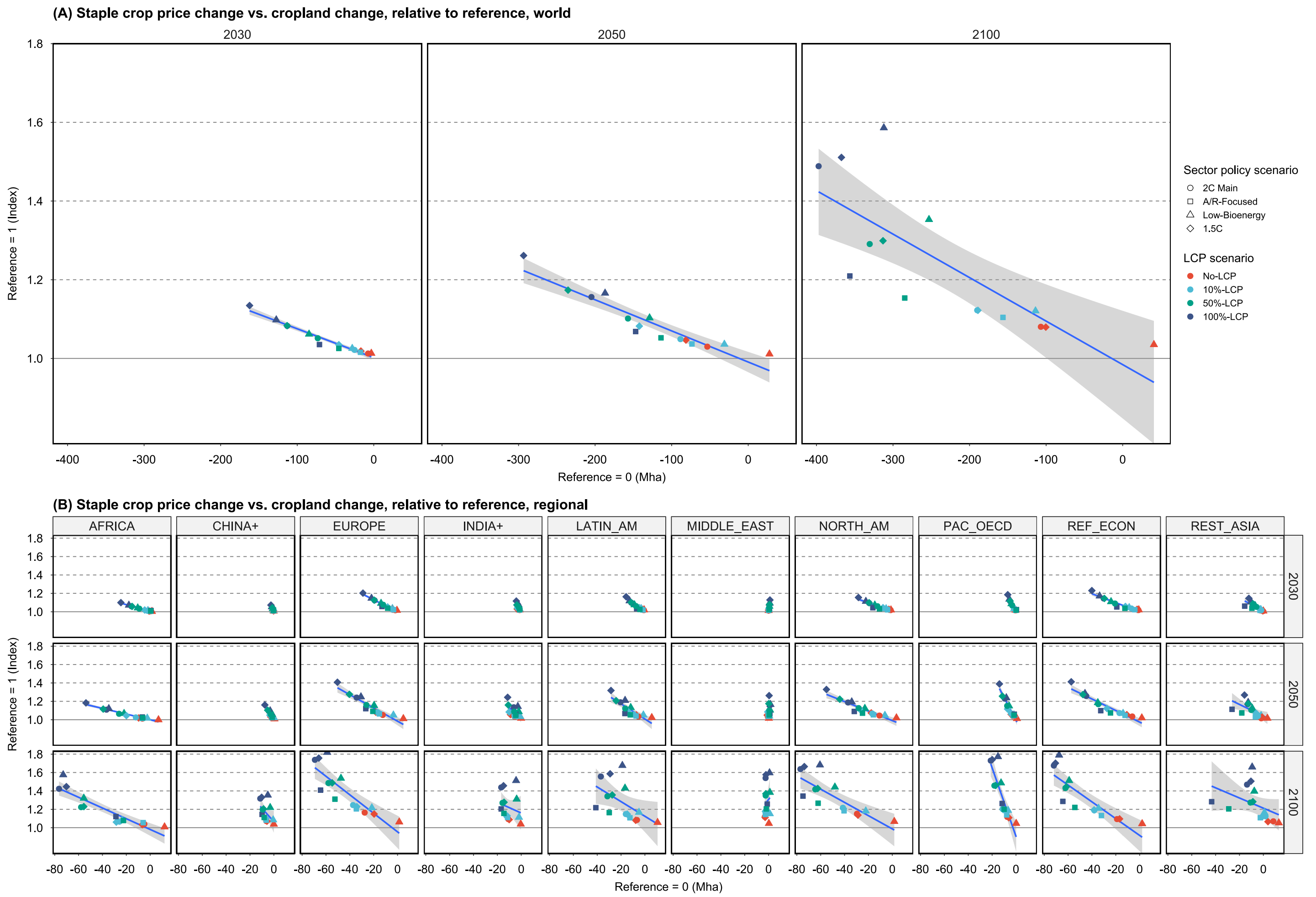

Fig. S33 | Relationship between staple crop price impact (reference = 1) and non-energy cropland impacts (reference = 0 Mha) at the world level (Panel A) and by AR6 R10 regions (Panel B). Points show the relationship by LCP scenarios (colors) and policy scenarios (shapes). Linear trend lines (blue lines) and a 95% confidence interval (grey ribbons) are added for each region (subpanel).

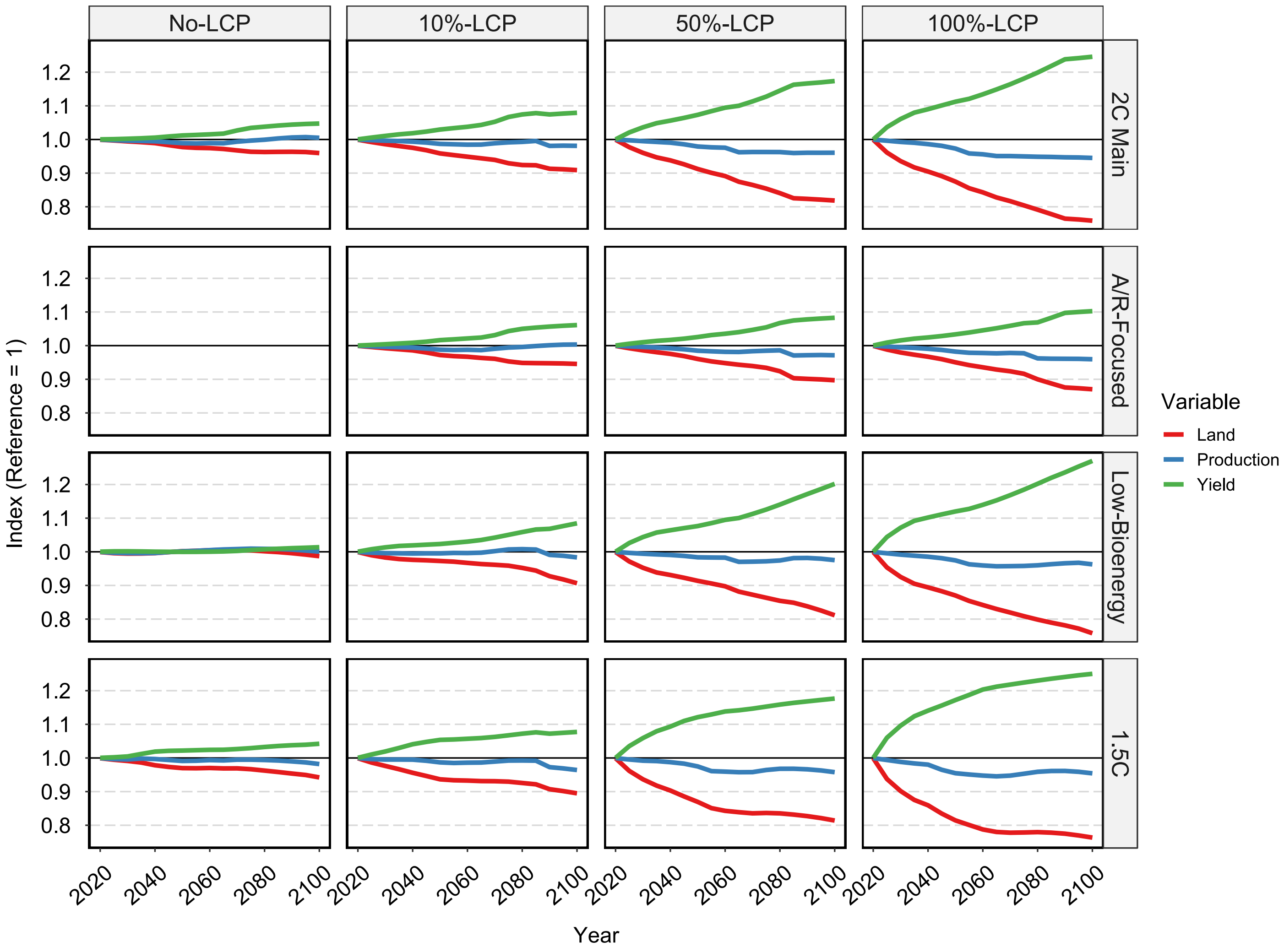

Fig. S34 | Changes in staple crop production, land area, and yield relative to the reference scenario. Lines show projections of the relative changes by variable (color) for staples at the world level.

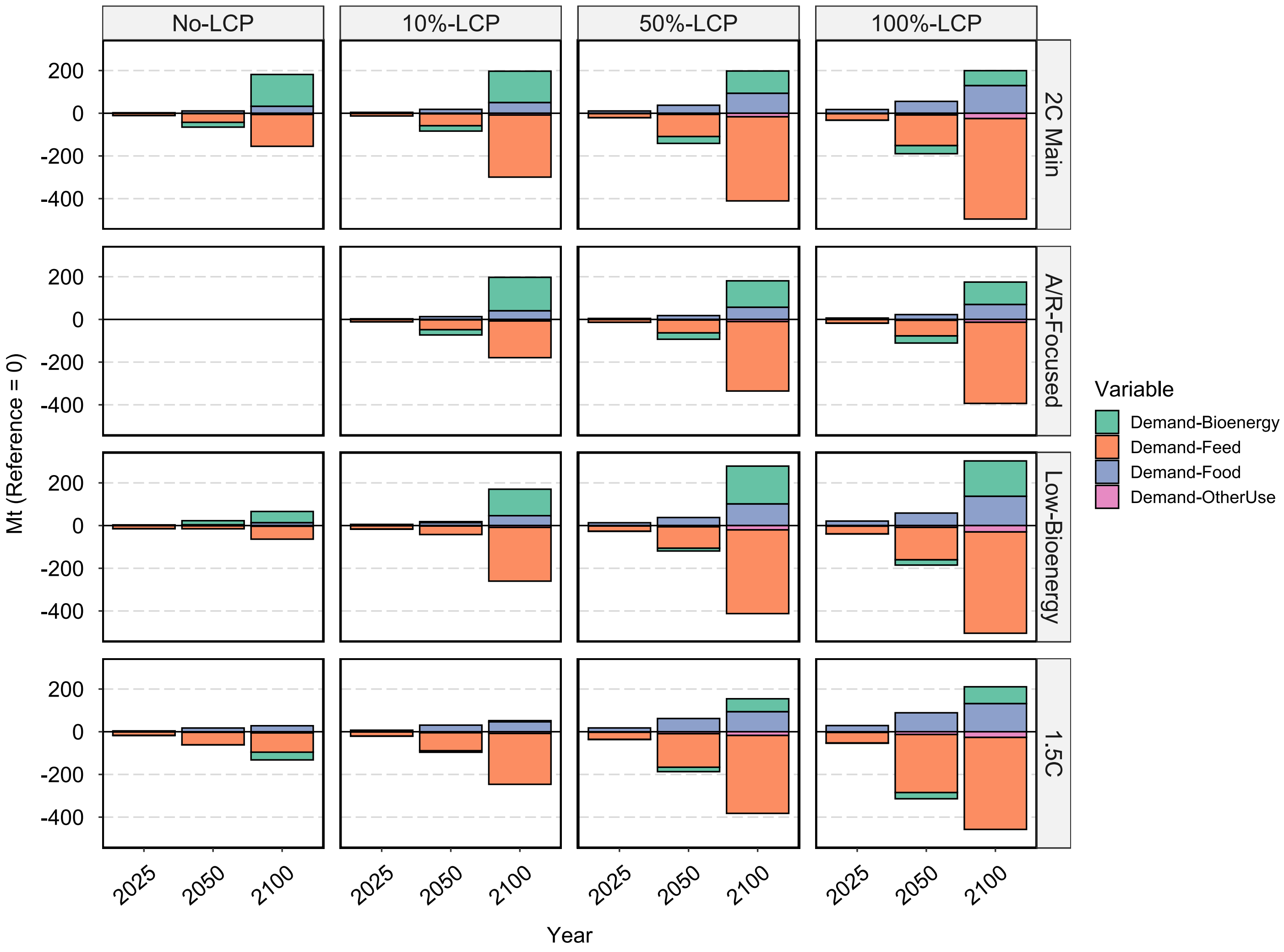

Fig. S35 | Changes in staple crop demand by source relative to the reference scenario. Bars show projections of the relative changes (Reference = 0 Mt) by variable (filled color) at the world level. Note that when the BECCS negative emission credit limit is reached, there could be a moderate increase in 1G bioenergy, which explains the "Demand-Bioenergy" increase in 2100. However, the 1G bioenergy is negligible compared to the 2G bioenergy in our study (see 1G bioenergy feedstock in **Fig. S15**).

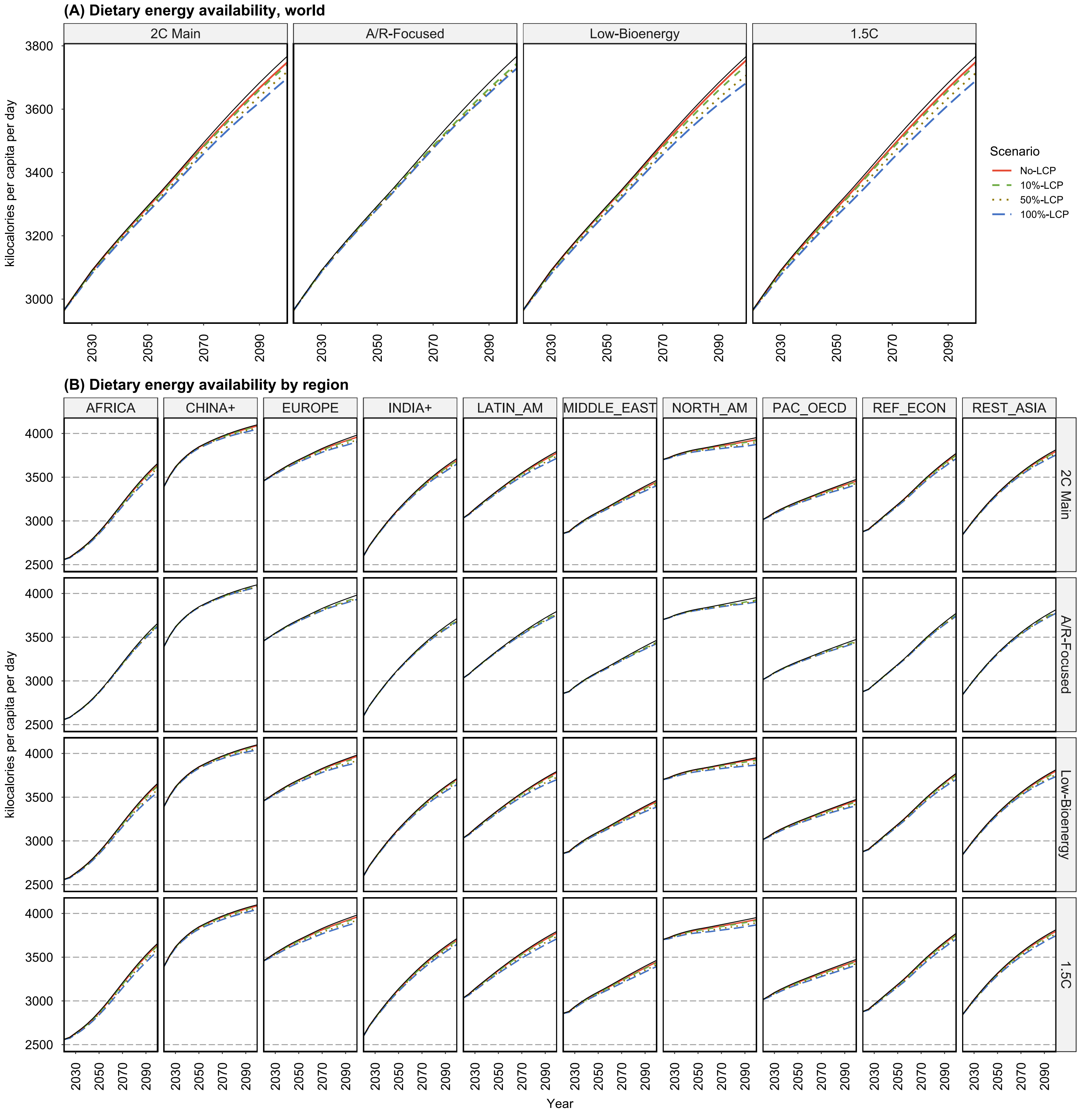

Fig. S36 | Projections of dietary energy availability at the world (Panel A) and regional (Panel B) levels. The black lines show the reference projections.

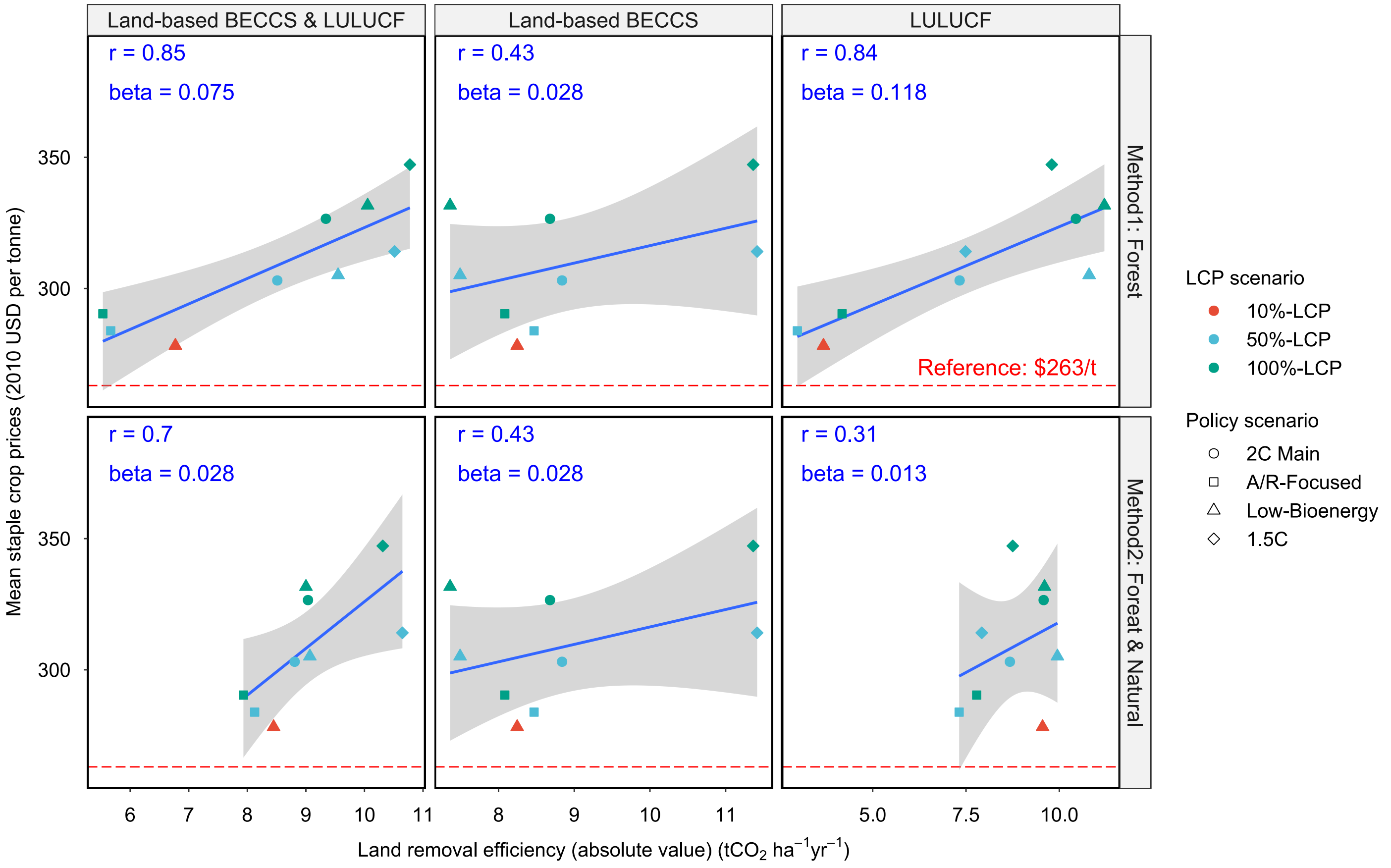

Fig. S37 | Relationship between agricultural prices and land carbon removal intensity. Points show the relationship between the mean staple crop prices and mean land removal intensity (2020 – 2100) by LCP scenarios (colors) and policy scenarios (shapes) for the removal intensity of energy crop-based BECCS, LULUCF, and the aggregate (energy crop-based BECCS & LULUCF). Two methods are employed for calculating the LULUCF removal intensity (**Method**), as indicated in the two panel rows. Crop prices are determined as production-weighted averages throughout the entire study period in mitigation pathways. The reference pathway is indicated by a red dotted line, representing a value of $286 per metric ton. Linear trend lines (blue lines) and 95% confidence intervals (grey ribbons) are included, and the correlation coefficient (r) and beta coefficient are provided.

Supplementary references

1. Byers, E. *et al.* AR6 Scenarios Database. (2022) doi:10.5281/ZENODO.7197970.
2. IPCC. Summary for Policymakers. in *Climate Change 2021: The Physical Science Basis. Contribution of Working Group I to the Sixth Assessment Report of the Intergovernmental Panel on Climate Change* (eds. Masson-Delmotte, V. et al.) 3−32 (Cambridge University Press, Cambridge, United Kingdom and New York, NY, USA, 2021).
3. IPCC. Annex III: Scenarios and modelling methods. in *Climate Change 2022: Mitigation of Climate Change* (Cambridge University Press, Cambridge, UK and New York, NY, USA, 2022).
4. Fajardy, M. *et al.* The economics of bioenergy with carbon capture and storage (BECCS) deployment in a 1.5 °C or 2 °C world. *Global Environmental Change* **68**, 102262 (2021).
5. Rogelj, J. *et al.* Scenarios towards limiting global mean temperature increase below 1.5 °C. *Nature Clim Change* **8**, 325–332 (2018).
6. Zhao, X., Calvin, K. V. & Wise, M. A. The critical role of conversion cost and comparative advantage in modeling agricultural land use change. *Clim. Change Econ.* **11**, 2050004 (2020).
7. Wise, M., Calvin, K., Kyle, G. P., Luckow, P. & Edmonds, J. ECONOMIC AND PHYSICAL MODELING OF LAND USE IN GCAM 3.0 AND AN APPLICATION TO AGRICULTURAL PRODUCTIVITY, LAND, AND TERRESTRIAL CARBON. *Climate Change Economics* **5**, 1–22 (2014).
8. Wise, M. *et al.* An approach to computing marginal land use change carbon intensities for bioenergy in policy applications. *Energy Economics* **50**, 337–347 (2015).
9. Calvin, K. *et al.* Trade-offs of different land and bioenergy policies on the path to achieving climate targets. *Climatic Change* **123**, 691–704 (2014).
10. Muratori, M., Calvin, K., Wise, M., Kyle, P. & Edmonds, J. Global economic consequences of deploying bioenergy with carbon capture and storage (BECCS). *Environ. Res. Lett.* **11**, 095004 (2016).
11. Wise, M., Dooley, J., Luckow, P., Calvin, K. & Kyle, P. Agriculture, land use, energy and carbon emission impacts of global biofuel mandates to mid-century. *Applied Energy* **114**, 763–773 (2014).
12. Sinha, E. *et al.* Implication of imposing fertilizer limitations on energy, agriculture, and land systems. *Journal of Environmental Management* **305**, 114391 (2022).
13. Kim, S. H. *et al.* Balancing global water availability and use at basin scale in an integrated assessment model. *Climatic Change* **136**, 217–231 (2016).
14. Edmonds, J. A., Link, R., Waldhoff, S. T. & Cui, R. A global food demand model for the assessment of complex human-earth systems. *Clim. Change Econ.* **08**, 1750012 (2017).
15. Zhao, X. *et al.* The impact of agricultural trade approaches on global economic modeling. *Global Environmental Change* **73**, 102413 (2022).
16. Zhao, X. *et al.* Global agricultural responses to interannual climate and biophysical variability. *Environ. Res. Lett.* **16**, 104037 (2021).
17. Fuhrman, J. *et al.* Diverse carbon dioxide removal approaches could reduce impacts on the energy–water–land system. *Nat. Clim. Chang.* 1–10 (2023) doi:10.1038/s41558-023-01604-9.
18. McJeon, H. *et al.* Fossil energy deployment through midcentury consistent with 2°C climate stabilization. *Energy and Climate Change* **2**, 100034 (2021).
19. Iyer, G. *et al.* Ratcheting of climate pledges needed to limit peak global warming. *Nat. Clim. Chang.* 1–7 (2022) doi:10.1038/s41558-022-01508-0.
20. Ou, Y. *et al.* Can updated climate pledges limit warming well below 2°C? *Science* **374**, 693–695 (2021).
21. Yarlagadda, B. *et al.* Trade and Climate Mitigation Interactions Create Agro-Economic Opportunities With Social and Environmental Trade-Offs in Latin America and the Caribbean. *Earth's Future* **11**, e2022EF003063 (2023).

22. Graham, N. T. *et al.* Humans drive future water scarcity changes across all Shared Socioeconomic Pathways. *Environ. Res. Lett.* **15**, 014007 (2020).
23. Di Vittorio, A. V., Narayan, K. B., Patel, P., Calvin, K. & Vernon, C. R. Doubling protected land area may be inefficient at preserving the extent of undeveloped land and could cause substantial regional shifts in land use. *GCB Bioenergy* **15**, 185–207 (2023).
24. Solomon, S. *et al.* IPCC fourth assessment report (AR4). *Climate change* **374**, (2007).
25. *World Agriculture towards 2030/2050: The 2012 Revision*. (2012). doi:10.22004/ag.econ.288998.
26. Alexandratos, N. *et al.* World agriculture: towards 2030/2050. Interim report. Prospects for food, nutrition, agriculture and major commodity groups. (2006).
27. FAOSTAT. FAOSTAT. https://www.fao.org/faostat/en/#data (2022).
28. PBL. Welcome to IMAGE 3.2 Documentation - IMAGE. https://models.pbl.nl/image/index.php/Welcome_to_IMAGE_3.2_Documentation (2023).
29. Ferreira Filho, J. B. de S., Ribera, L. & Horridge, M. Deforestation Control and Agricultural Supply in Brazil. *American Journal of Agricultural Economics* **97**, 589–601 (2015).
30. Li, C. & Wu, J. Land use transformation and eco-environmental effects based on production-living-ecological spatial synergy: evidence from Shaanxi Province, China. *Environ Sci Pollut Res* (2022) doi:10.1007/s11356-022-18777-z.
31. Zhao, X., van der Mensbrugghe, D. Y., Keeney, R. M. & Tyner, W. E. Improving the way land use change is handled in economic models. *Economic Modelling* **84**, 13–26 (2020).
32. Taheripour, F., Zhao, X., Horridge, M., Farrokhi, F. & Tyner, W. Land Use in Computable General Equilibrium Models. *Journal of Global Economic Analysis* **5**, 63–109 (2020).
33. Fujimori, S., Hasegawa, T., Masui, T. & Takahashi, K. Land use representation in a global CGE model for long-term simulation: CET vs. logit functions. *Food Sec.* **6**, 685–699 (2014).
34. Di Vittorio, A. V., Vernon, C. R. & Shu, S. Moirai Version 3: A Data Processing System to Generate Recent Historical Land Inputs for Global Modeling Applications at Various Scales. **8**, 1 (2020).
35. Calvin, K. *et al.* GCAM v5.1: representing the linkages between energy, water, land, climate, and economic systems. *Geoscientific Model Development* **12**, 677–698 (2019).
36. Narayan, K., Vernon, C., Vittorio, A. D. & EvanMargiotta. JGCRI/moirai: Moirai v3.1.1. (2022) doi:10.5281/zenodo.6632745.
37. IUCN. World database on protected areas. https://www.protectedplanet.net (2018).
38. Zabel, F., Putzenlechner, B. & Mauser, W. Global Agricultural Land Resources – A High Resolution Suitability Evaluation and Its Perspectives until 2100 under Climate Change Conditions. *PLOS ONE* **9**, e107522 (2014).
39. Ahmed, S. A., Hertel, T. & Lubowski, R. Calibration of a Land Cover Supply Function Using Transition Probabilities. *GTAP Research Memorandum No. 14* http://www.gtap.agecon.purdue.edu/resources/res_display.asp?RecordID=2947 (2008).
40. Ramankutty, N., Hertel, T. & Lee, H.-L. Global Land Use and Land Cover Data for Integrated Assessment Modeling. *GTAP* http://www.gtap.agecon.purdue.edu/resources/res_display.asp?RecordID=1635 (2005).
41. Hashida, Y. & Lewis, D. J. The Intersection between Climate Adaptation, Mitigation, and Natural Resources: An Empirical Analysis of Forest Management. *Journal of the Association of Environmental and Resource Economists* **6**, 893–926 (2019).
42. Tahvonen, O. & Rautiainen, A. Economics of forest carbon storage and the additionality principle. *Resource and Energy Economics* **50**, 124–134 (2017).
43. Lintunen, J., Laturi, J. & Uusivuori, J. How should a forest carbon rent policy be implemented? *Forest Policy and Economics* **69**, 31–39 (2016).

44. Hengl, T. *et al.* SoilGrids250m: Global gridded soil information based on machine learning. *PLOS ONE* **12**, e0169748 (2017).
45. Spawn, S. A., Sullivan, C. C., Lark, T. J. & Gibbs, H. K. Harmonized global maps of above and belowground biomass carbon density in the year 2010. *Sci Data* **7**, 112 (2020).
46. Ledo, A. *et al.* Changes in soil organic carbon under perennial crops. *Global Change Biology* **26**, 4158–4168 (2020).
47. Zhao, X. *et al. Gcamfaostat: An R Prepare, Process, and Synthesize FAOSTAT Data for Global Agroeconomic and Multisector Dynamic Modeling*. https://jgcri.github.io/gcamfaostat (2024).
48. Zhao, X., Calvin, K. V., Wise, M. A. & Iyer, G. The role of global agricultural market integration in multiregional economic modeling: Using hindcast experiments to validate an Armington model. *Economic Analysis and Policy* **72**, 1–17 (2021).
49. Gregg, J. S. & Smith, S. J. Global and regional potential for bioenergy from agricultural and forestry residue biomass. *Mitig Adapt Strateg Glob Change* **15**, 241–262 (2010).
50. Hanssen, S. V. *et al.* Biomass residues as twenty-first century bioenergy feedstock—a comparison of eight integrated assessment models. *Climatic Change* **163**, 1569–1586 (2020).
51. Searle, S. & Malins, C. A reassessment of global bioenergy potential in 2050. *GCB Bioenergy* **7**, 328–336 (2015).
52. Smith, W. A., Wendt, L. M., Bonner, I. J. & Murphy, J. A. Effects of Storage Moisture Content on Corn Stover Biomass Stability, Composition, and Conversion Efficacy. *Frontiers in Bioengineering and Biotechnology* **8**, (2020).
53. Cafferty, K. G., Muth, D. J., Jacobson, J. J. & Bryden, K. M. Model Based Biomass System Design of Feedstock Supply Systems for Bioenergy Production. in (American Society of Mechanical Engineers Digital Collection, 2014). doi:10.1115/DETC2013-13559.
54. Alcalde, J. *et al.* Estimating geological CO2 storage security to deliver on climate mitigation. *Nat Commun* **9**, 2201 (2018).
55. Dooley, J. J. & Dahowski, R. T. Large-Scale U.S. Unconventional Fuels Production and the Role of Carbon Dioxide Capture and Storage Technologies in Reducing Their Greenhouse Gas Emissions. *Energy Procedia* **1**, 4225–4232 (2009).
56. Sampedro, J. *et al.* Dynamic linking of upstream energy and freight demands for bio and fossil energy pathways in the Global Change Analysis Model. *Applied Energy* **302**, 117580 (2021).
57. Chen, R. *et al.* Life cycle energy and greenhouse gas emission effects of biodiesel in the United States with induced land use change impacts. *Bioresource Technology* **251**, 249–258 (2018).
58. Lee, U., Kwon, H., Wu, M. & Wang, M. Retrospective analysis of the U.S. corn ethanol industry for 2005–2019: implications for greenhouse gas emission reductions. *Biofuels, Bioproducts and Biorefining* **15**, 1318–1331 (2021).
59. Wang, M., Han, J., Dunn, J. B., Cai, H. & Elgowainy, A. Well-to-wheels energy use and greenhouse gas emissions of ethanol from corn, sugarcane and cellulosic biomass for US use. *Environ. Res. Lett.* **7**, 045905 (2012).
60. Prussi, M. *et al.* CORSIA: The first internationally adopted approach to calculate life-cycle GHG emissions for aviation fuels. *Renewable and Sustainable Energy Reviews* **150**, 111398 (2021).
61. Strefler, J. *et al.* Alternative carbon price trajectories can avoid excessive carbon removal. *Nat Commun* **12**, 2264 (2021).
62. Bauer, N. *et al.* Bio-energy and CO2 emission reductions: an integrated land-use and energy sector perspective. *Climatic Change* **163**, 1675–1693 (2020).

63. Morris, J., Paltsev, S. & Reilly, J. Marginal Abatement Costs and Marginal Welfare Costs for Greenhouse Gas Emissions Reductions: Results from the EPPA Model. *Environ Model Assess* **17**, 325–336 (2012).
64. Fuhrman, J. *et al.* Food–energy–water implications of negative emissions technologies in a +1.5 °C future. *Nat. Clim. Chang.* **10**, 920–927 (2020).
65. Dixon, P., van Meijl, H., Rimmer, M., Shutes, L. & Tabeau, A. RED versus REDD: Biofuel policy versus forest conservation. *Economic Modelling* **52**, 366–374 (2016).
66. Villoria, N., Garrett, R., Gollnow, F. & Carlson, K. Leakage does not fully offset soy supply-chain efforts to reduce deforestation in Brazil. *Nat Commun* **13**, 5476 (2022).
67. Erbaugh, J. T. Impermanence and failure: The legacy of conservation-based payments in Sumatra, Indonesia. *Environ. Res. Lett.* (2022) doi:10.1088/1748-9326/ac6437.
68. Grassi, G. *et al.* Critical adjustment of land mitigation pathways for assessing countries' climate progress. *Nat. Clim. Chang.* **11**, 425–434 (2021).
69. Roe, S. *et al.* Land-based measures to mitigate climate change: Potential and feasibility by country. *Global Change Biology* **27**, 6025–6058 (2021).
70. Cook-Patton, S. C. *et al.* Mapping carbon accumulation potential from global natural forest regrowth. *Nature* **585**, 545–550 (2020).
71. Griscom, B. W. *et al.* Natural climate solutions. *PNAS* **114**, 11645–11650 (2017).
72. Austin, K. G. *et al.* The economic costs of planting, preserving, and managing the world's forests to mitigate climate change. *Nat Commun* **11**, 5946 (2020).
73. Xu, S. *et al.* Delayed use of bioenergy crops might threaten climate and food security. *Nature* **609**, 299–306 (2022).
74. Jägermeyr, J. *et al.* Climate impacts on global agriculture emerge earlier in new generation of climate and crop models. *Nat Food* **2**, 873–885 (2021).
75. Calvin, K. *et al.* Global market and economic welfare implications of changes in agricultural yields due to climate change. *Clim. Change Econ.* **11**, 2050005 (2020).
76. Chiquier, S., Patrizio, P., Bui, M., Sunny, N. & Dowell, N. M. A comparative analysis of the efficiency, timing, and permanence of CO 2 removal pathways. *Energy & Environmental Science* **15**, 4389–4403 (2022).
77. Echeverria, J. D. Regulating versus Paying Land Owners to Protect the Environment. *J. Land Resources & Envtl. L.* **26**, 1 (2005).
78. Joppa, L. N., Loarie, S. R. & Pimm, S. L. On the protection of "protected areas". *Proceedings of the National Academy of Sciences* **105**, 6673–6678 (2008).
79. Jones, K. R. *et al.* One-third of global protected land is under intense human pressure. *Science* **360**, 788–791 (2018).
80. Huang, X., Srikrishnan, V., Lamontagne, J., Keller, K. & Peng, W. Effects of global climate mitigation on regional air quality and health. *Nat Sustain* 1–13 (2023) doi:10.1038/s41893-023-01133-5.
81. Leclère, D. *et al.* Bending the curve of terrestrial biodiversity needs an integrated strategy. *Nature* **585**, 551–556 (2020).
82. Gouel, C. & Laborde, D. The crucial role of domestic and international market-mediated adaptation to climate change. *Journal of Environmental Economics and Management* **106**, 102408 (2021).
83. Yao, G., Hertel, T. W. & Taheripour, F. Economic drivers of telecoupling and terrestrial carbon fluxes in the global soybean complex. *Global Environmental Change* **50**, 190–200 (2018).
84. Windisch, M. G., Davin, E. L. & Seneviratne, S. I. Prioritizing forestation based on biogeochemical and local biogeophysical impacts. *Nat. Clim. Chang.* **11**, 867–871 (2021).

85. Matthews, H. D. *et al.* Opportunities and challenges in using remaining carbon budgets to guide climate policy. *Nat. Geosci.* **13**, 769–779 (2020).
86. Dorheim, K., Link, R., Hartin, C., Kravitz, B. & Snyder, A. Calibrating Simple Climate Models to Individual Earth System Models: Lessons Learned From Calibrating Hector. *Earth and Space Science* **7**, e2019EA000980 (2020).
87. Zhao, X. & Wise, M. *GCAM Agriculture and Land Use (AgLU) Data and Method Updates: Connecting Land Hectares to Food Calories*. https://jgcri.github.io/gcam-doc/cmp/CMP_360-AgLU_data_method_updates.pdf (2023).
